# Impact of insertion sequences on convergent evolution of *Shigella* species

**DOI:** 10.1371/journal.pgen.1008931

**Published:** 2020-07-09

**Authors:** Jane Hawkey, Jonathan M. Monk, Helen Billman-Jacobe, Bernhard Palsson, Kathryn E. Holt

**Affiliations:** 1 Department of Infectious Diseases, Central Clinical School, Monash University, Melbourne, Victoria, Australia; 2 Department of Bioengineering, University of California, San Diego, San Diego, California, United States of America; 3 Faculty of Veterinary and Agricultural Sciences, University of Melbourne, Parkville, Victoria, Australia; 4 The London School of Hygiene and Tropical Medicine, London, WC1E 7HT, United Kingdom; Uppsala University, SWEDEN

## Abstract

*Shigella* species are specialised lineages of *Escherichia coli* that have converged to become human-adapted and cause dysentery by invading human gut epithelial cells. Most studies of *Shigella* evolution have been restricted to comparisons of single representatives of each species; and population genomic studies of individual *Shigella* species have focused on genomic variation caused by single nucleotide variants and ignored the contribution of insertion sequences (IS) which are highly prevalent in *Shigella* genomes. Here, we investigate the distribution and evolutionary dynamics of IS within populations of *Shigella dysenteriae* Sd1, *Shigella sonnei* and *Shigella flexneri*. We find that five IS (IS*1*, IS*2*, IS*4*, IS*600* and IS*911*) have undergone expansion in all *Shigella* species, creating substantial strain-to-strain variation within each population and contributing to convergent patterns of functional gene loss within and between species. We find that IS expansion and genome degradation are most advanced in *S*. *dysenteriae* and least advanced in *S*. *sonnei*; and using genome-scale models of metabolism we show that *Shigella* species display convergent loss of core *E*. *coli* metabolic capabilities, with *S*. *sonnei* and *S*. *flexneri* following a similar trajectory of metabolic streamlining to that of *S*. *dysenteriae*. This study highlights the importance of IS to the evolution of *Shigella* and provides a framework for the investigation of IS dynamics and metabolic reduction in other bacterial species.

## Author summary

Originally classified as a genus due to shared disease features, *Shigella* species actually represent distinct lineages of *Escherichia coli* that have become human-restricted dysentery pathogens via convergent evolution. A hallmark of this host-adaptation is the accumulation of insertion sequences (IS), which interrupt genes and cause deletions or genome rearrangements. Due to technical limitations in identifying IS insertions from short-read data, our understanding of the contribution of IS to the evolution of *Shigella* and other host- restricted bacteria has relied on analyses of either single genome representatives, or small numbers of genomes, and there are no large-scale population genomic studies exploring IS dynamics within bacterial species. Here we explore IS variation within and between *Shigella* species, revealing their historical and ongoing contributions to evolutionary convergence and metabolic streamlining.

## Introduction

**Fig 1:**
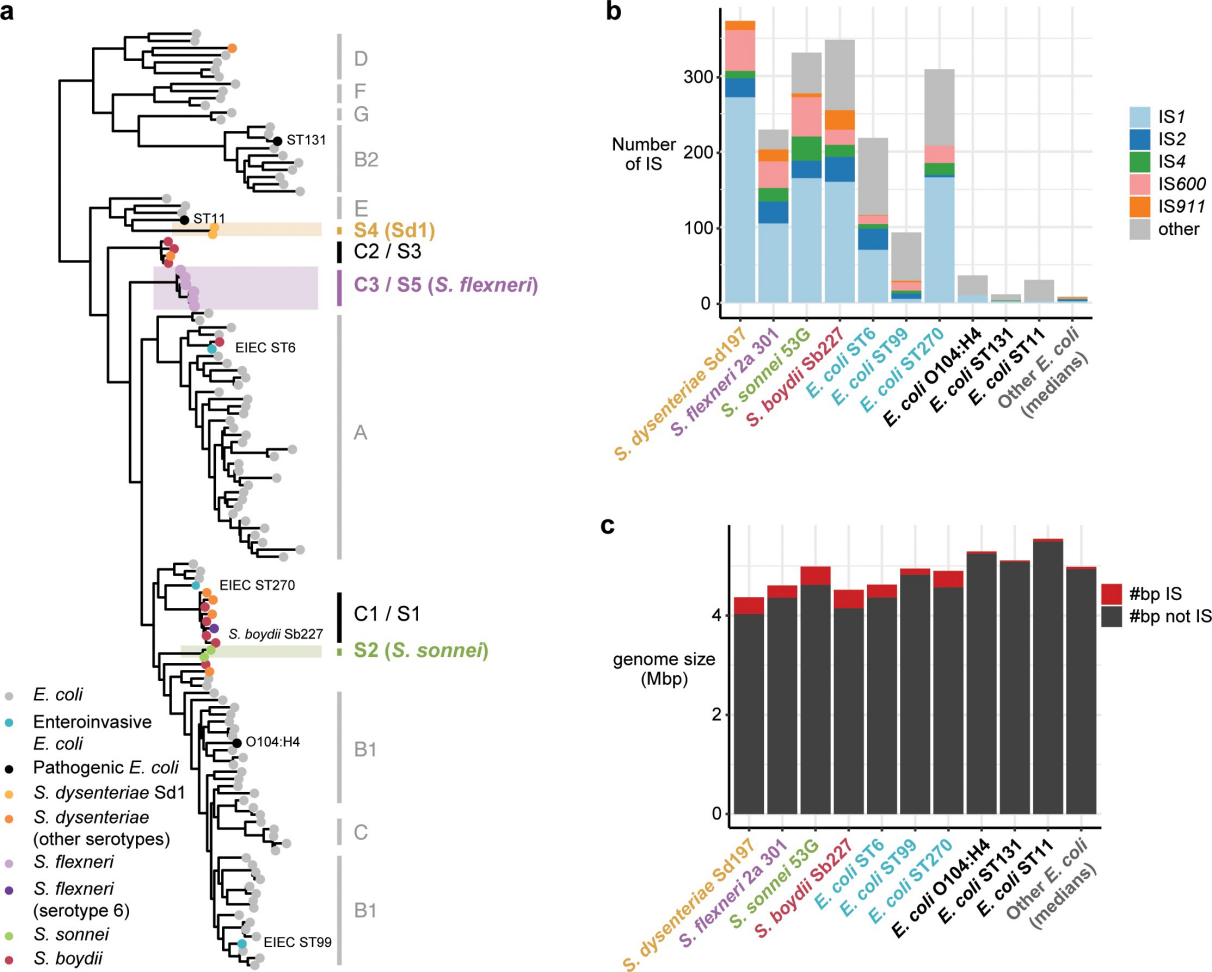
Summary of reference genomes used in this study. **(a)** Midpoint rooted maximum likelihood core-gene phylogeny of all *Shigella* and *E*. *coli* reference genomes used in this study (tips coloured as per legend) and a set of 100 non-redundant *E*. *coli* genomes (grey tips). *E*. *coli* phylogroups are labelled in grey, with *Shigella* clades labelled as per Yang et al 2007 (C designations) and Sahl et al 2015 (S designations). Grey boxes indicate the three *Shigella* lineages that are analysed in this study. **(b)** Bar plot showing total number of IS in each reference genome (names coloured as panel **a**), broken by IS type as per legend. Final bar shows median number of each IS found amongst the other *E*. *coli* (grey tips in panel **a**). **(c)** Bar plots showing total genome size for each reference genome, broken down by number of bases belonging to IS sequence (red) vs not IS sequence (black).

*Shigellae* are Gram-negative intracellular bacterial pathogens transmitted via the faecal-oral route and are the bacterial agents of dysentery [1,2]. Four species are currently defined on the basis of serotypes: *Shigella dysenteriae*, *Shigella flexneri*, *Shigella sonnei* and *Shigella boydii*. *S*. *flexneri* and *S*. *sonnei* are endemic in human populations globally and contribute most to the dysentery disease burden [1]; *S*. *dysenteriae* is associated with epidemic bacillary dysentery [3] and *S*. *boydii* is rare and mostly restricted to the Indian sub-continent [1]. All *Shigella* are restricted to the human host, with no known animal or environmental reservoirs. DNA sequence analyses show that the serologically defined *Shigella* ‘species’ are in fact paraphyletic members of species *Escherichia coli* (see **Fig 1A**), which have converged on similar human-adapted dysentery-associated phenotypes through parallel evolutionary processes [4–8]. These include gains of function via acquisition of the virulence plasmid pINV which carries genes for invasive infection including the *mix-spa* locus encoding a type 3 secretion system [9], and of genomic islands SHI-1, which carries several toxins [10], and SHI-2, which encodes the siderophore aerobactin as well as bactericidal and immune evasion genes.

Genome degradation–a common signature of host-restricted pathogens associated with passing through an evolutionary bottleneck and adapting to a narrow host range [11–14] –is also recognised as an important part of the convergent evolution of *Shigella* species [15]. Unlike most *E*. *coli*, *Shigella* species are non-motile due to inactivation of flagella and also lack fimbrial adhesins [15], which likely impedes host immune recognition [16,17]. Four metabolic pathways present in *E*. *coli* are known to be deleted or inactivated in all *Shigella* [18]: *nadA/nadB*, which are responsible for the nicotine acid pathway [19,20]; *cadA*, which encodes a lysine decarboxylase [15]; *speG* which converts spermidine into the non-reactive acetylspermidine [21]; and *ompT*, an outer membrane protease [15]. Restoring function for each of these pathways has been shown to interfere with the ability of *Shigella* to cause disease in humans [18].

The degradation of *Shigella* genomes is associated with a variety of mutational processes including deletions, nonsense mutations, and insertion sequences (IS)–small transposable elements that are ubiquitous in *Shigella* genomes and contribute to functional gene loss by disrupting coding sequences or facilitating genome rearrangements and deletions between homologous IS copies [15,22]. In other bacterial pathogens, IS have also been shown to influence gene expression [23]. Within *Shigella*, loss of the flagella *flhDC* and curli *csg* operons in each species has been mediated by IS [5,18], as well as the inactivation of the *cadA* gene in *S*. *sonnei* [18]. Yang et al recognised the high number of IS in individual *Shigella* genomes (one per species), including quantifying which IS were present and hypothesised that they were the likely causative agent of the observed large-scale genome rearrangements [15]. Other reviews of *Shigella* have simply noted that IS are overrepresented in *Shigella* genomes or detailed specific instances of IS-mediated gene deletion of important metabolic pathways [18]. To date no studies have examined in detail the impact IS have had on the evolutionary history of *Shigella* or compared their impact to that of other mutational processes.

Recent genomic studies of *S*. *flexneri*, *S*. *dysenteriae*, and *S*. *sonnei* have begun to elucidate the finer population structure of the major clades of these species (S5, S4 and S2, respectively, according to the nomenclature of Sahl *et al* [7]; see **Fig 1A**). The *S*. *flexneri* study focused on clade S5 (C3 according to the nomenclature of Yang *et al* [8]) which comprises serotypes 1, 2, 3, 4, 5, X and Y (**Fig 1A**). These serotypes accounted for 80% of *S*. *flexneri* identified in the paediatric Global Enteric Multicenter Study (GEMS) [24]. *S*. *flexneri* clade S5/C3 has seven deep branching phylogenetic lineages, which are separated from one another by mean 0.089% nucleotide divergence and 150–600 years of evolution, are each broadly geographically distributed, and cause endemic disease in developing countries [25]. The *S*. *dysenteriae* study focused on type 1 (Sd1), which is the dominant agent of epidemic bacillary dysentery and forms a single monophyletic clade (S4) in the *E*. *coli* tree (**Fig 1A**). Phylogenomic analysis showed that Sd1 isolated from dysentery outbreaks spanning the last century share a recent common ancestor in the 18^th^ century, which has diverged into four lineages (mean 0.017% divergence) that spread globally during the late 19^th^ century [26]. *S*. *sonnei* are monophyletic in the *E*. *coli* tree (clade S2, **Fig 1A**) and accounted for 24% of all *Shigella* cases in GEMS [24]. Phylogenomic analysis showed *S*. *sonnei* isolates from the last 80 years share a common ancestor in the late 17^th^ century and the population has since diverged into three major lineages that are ~0.023% divergent from one another [27]. Lineage III is currently the most prevalent and geographically widespread [27], and is replacing circulating lineages of *S*. *flexneri* in developing nations [28,29].

The population structure and natural history of *S*. *boydii* has not yet been clearly elucidated. Currently available *S*. *boydii* genomes are broadly distributed across multiple clades in the *E*. *coli* phylogeny (see **Fig 1A**), including clades S3/C2 (together with multiple non-Sd1 *S*. *dysenteriae* serotypes) and clade S1/C1 (together with *S*. *flexneri* serotype 6 and non-Sd1 *S*. *dysenteriae*). The largest comparative study of *S*. *boydii* genomes (n = 42 genomes) confirmed that isolates assigned to this ‘species’ by serology are paraphyletic [30]. The largest monophyletic *S*. *boydii* clade in that study (part of S1/C1) contained just n = 18 genomes, and there are no published studies investigating the evolution or population structure of particular *S*. *boydii* clades or of the mixed *Shigella* clades, S1/C1 or S3/C2, to which most *S*. *boydii* belong. This is likely because these clades are much rarer amongst human dysentery cases, comprising just 5% of *Shigella* isolated in GEMS [24].

The published population genomic studies of *S*. *sonnei*, *S*. *flexneri* S5 and *S*. *dysenteriae* Sd1 primarily focused on elucidating population structure defined by single nucleotide variants (SNVs), which are readily extracted from high-throughput short read sequencing data [25–27]; however they did not investigate IS dynamics within each population nor their contribution to convergent evolution between *Shigella* species or clades. Here we apply new IS detection and genome-scale metabolic modelling tools to investigate and compare IS dynamics within the three major *Shigella* clades *S*. *sonnei*, *S*. *flexneri* S5 and *S*. *dysenteriae* Sd1 (shaded in **Fig 1A**), contextualised within the broader *E*. *coli* species. We identify five IS that have undergone dramatic parallel expansions in *Shigella* but not other *E*. *coli*, and reconstruct their evolutionary history in the three major *Shigella* clades. Notably these clades are responsible for the majority of the global dysentery disease burden: *S*. *dysenteriae* Sd1 (responsible for majority of epidemic dysentery); *S*. *sonnei* and *S*. *flexneri* S5 (main contributors to the endemic disease burden, together responsible for >76% of *Shigella* cases in GEMS [24]). We examine the contribution of IS to past and ongoing functional diversification within and between the major *Shigella* clades, and elucidate convergent patterns of metabolic pathway loss.

## Results

### IS distribution in *Shigella* populations

Genome size, gene counts and IS counts for the various *Shigella* reference genomes and selected *E*. *coli* reference genomes are shown in **Fig 1B and 1C**. As previously reported [15], *Shigella* chromosomes are significantly smaller than other *E*. *coli* (mean 4.6 Mbp vs 4.9 Mbp; p = 0.03 using Wilcoxon test and counting one strain per lineage), with more IS (median 339 vs 32; p = 0.0007), which account for 5–8% of bases in the *Shigella* genomes compared to <1% in other *E*. *coli* (p = 0.0007; see **Fig 1C; S1 Table**). *S*. *dysenteriae* Sd1 has the smallest chromosome (4.3 Mbp), with the smallest coding capacity (4270 intact protein coding sequences (CDS)) and highest density of IS (9.26 per 100 kbp of sequence, 13–42 times that of other *E*. *coli*; see **S1 Table**).

**Fig 2:**
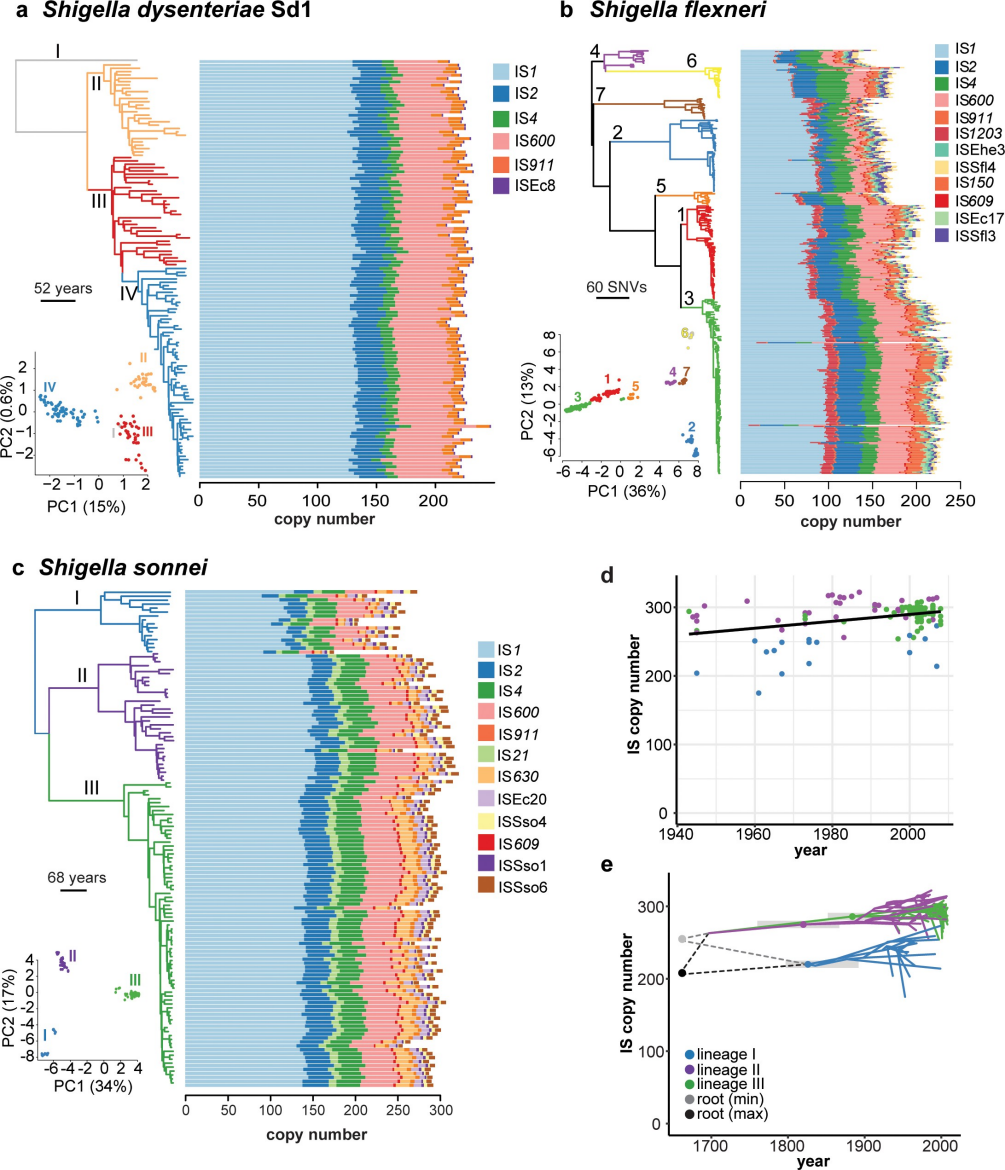
IS found in each *Shigella* species. **(a)** Time-calibrated Bayesian (BEAST) phylogeny of 125 *S*. *dysenteriae* genomes next to bar plots showing IS copy number in each genome. Inset, PCA of IS insertion site matrix, with points coloured by lineage as indicated by tree branch colours. **(b)** Same for 343 *S*. *flexneri* genomes but with a midpoint rooted maximum-likelihood phylogeny. **(c)** Same for 126 *S*. *sonnei* genomes (time-calibrated Bayesian phylogeny). **(d)** Scatterplot of IS copy number (inferred using ISMapper) on year of isolation, for 126 the *S*. *sonnei* genomes. Points are coloured by lineage, as per tree branch colours in (c). Fitted lines show linear regression of IS copy number against year for each lineage, with a single slope fit to all lineages. Dashed horizontal lines indicate the total IS copy number estimated in each lineage’s MRCA using ancestral state reconstruction; grey boxes show 95% HPD intervals for the date of each lineage MRCA estimated from the BEAST analysis. **(e)** Phenogram of *S*. *sonnei* time-calibrated tree from panel (c), mapped to y axis to indicate IS copy number inferred at each node on the tree based on ancestral state reconstruction. Branches are coloured by lineage as per legend. Dashed lines indicate two possible reconstructions for the lower and upper bound of IS burden at the root.

The *Shigella* reference chromosomes each harboured 229–348 IS insertions, including five IS common to all species (IS*1*, IS*2*, IS*4*, IS*600*, IS*911*) and up to eight additional IS per species (**S1 Table**). We used ISMapper [31] to identify chromosomal insertion sites for these IS in short-read data sets for the three major *Shigella* global genome collections (n = 125 *S*. *dysenteriae* serotype 1 (clade S4 in **Fig 1A,** hereafter referred to simply as *S*. *dysenteriae*) [26], n = 343 *S*. *flexneri* (clade S5 in **Fig 1A,** hereafter referred to simply as *S*. *flexneri*) [25], n = 126 *S*. *sonnei* [27]. ISMapper analysis detected a median of 286 IS insertions per chromosome (range 175–322) in *S*. *sonnei*, 194 (range 132–241) in *S*. *flexneri* and 197 (range 183–219) in *S*. *dysenteriae* (**Fig 2, S1 Table**). These numbers are lower (median 53–86%) than those identified in completed reference chromosomes (**Fig 1B**) [15]. This underestimation is expected because ISMapper detects insertion sites relative to an IS-free reference sequence for each species [31], and cannot detect insertions of IS within other IS (which does occur in the reference genomes) [15]. IS are also present in the virulence plasmid sequences of finished *Shigella* reference genomes [15] (**S2 Table**); however as the plasmid is frequently lost during culture [32] and was lacking from many of the short read data sets [27], IS variation in the virulence plasmid was not further examined in this study. There may be additional IS present in these genomes that were not found in the reference genome. However these IS are likely to be rare, as they are not conserved in the population, and therefore were not present during the bottleneck event that occurred when each species adapted to humans; thus are likely to have relatively minor impact on evolutionary patterns compared to the major five IS that have been expanding in these species clades since their emergence. Across all genomes, n = 609 unique IS insertion sites were identified in the *S*. *dysenteriae* chromosome, n = 1,778 in *S*. *flexneri* and n = 1,227 in *S*. *sonnei* (**S1–S3 Figs, S3–S5 Tables**).

Within each *Shigella* species, we observed substantial variation in the number (**Fig 2**) and location (**S1–S3 Figs, S3–S5 Tables**) of IS insertion sites detected, to the extent that phylogenetic lineages within each species could generally be distinguished from one another by IS insertion profiles alone (see insets, **Fig 2A–2C**). This indicates IS transposition has been a persistent feature of *Shigella* genomes during the diversification of each population from its most recent common ancestor (MRCA), represented by the root of each species tree in **Fig 2A–2C**. Strain-specific IS insertion sites, which reflect recent transposition events since the divergence of each isolate from its nearest relative in the sampled population, were identified in 67% of *S*. *dysenteriae* genomes, 60% *of S*. *flexneri* genomes and 75% of *S*. *sonnei* genomes, indicating IS continue to generate population diversity in each species.

### IS dynamics and population structure

**Fig 3:**
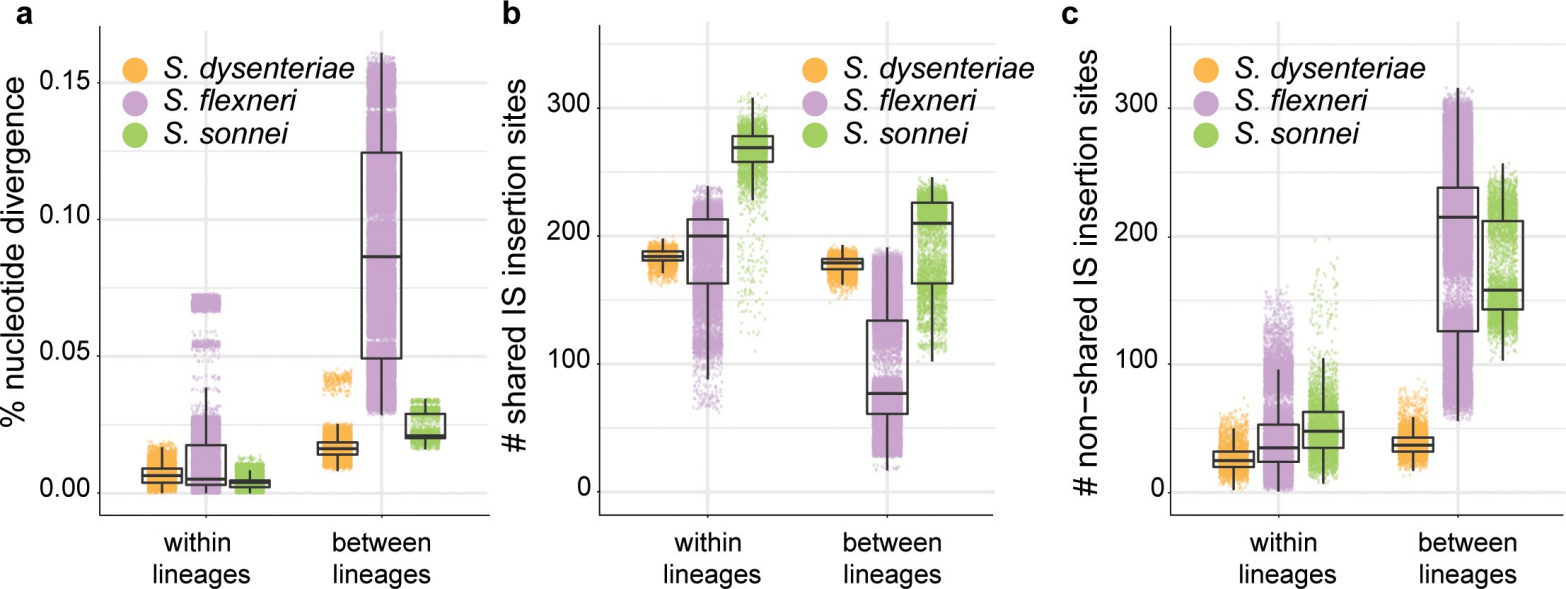
Comparison of nucleotide and IS-profile similarities across *Shigella* species. **a,** Pairwise nucleotide divergence for genomes in each *Shigella* population, estimated from mapping-based SNV counts. **b-c,** Pairwise comparisons of shared (**b**) and non-shared (**c**) IS insertion sites for genomes in each *Shigella* population, based on ISMapper analysis.

The three phylogenetic lineages of *S*. *sonnei* each showed highly differentiated IS profiles (inset, **Fig 2C**), with pairs of isolates from different lineages sharing only 53% of their IS insertion sites (compared to mean 84% of IS sites shared between pairs from the same lineage, **Fig 3B**). However within each lineage, the temporal dynamics of IS accumulation were quite similar. Linear regression of IS insertion count on year of isolation for the observed *S*. *sonnei* genomes estimated a mean contemporary IS accumulation rate of 0.34 IS per year (**Fig 2D**). The correlation between IS and date of isolation was significant (p = 0.001 using a date randomisation test to account for phylogenetic non-independence, see **S4 Fig**). To more directly model the recent evolutionary history of IS expansion in *S*. *sonnei*, we used maximum parsimony ancestral state reconstruction to infer the presence/absence of each IS insertion at internal nodes of the dated phylogeny (**Fig 2E**), and interpreted transitions between inferred states at linked nodes as IS gain/loss events (see **Methods**). The total number of gains across the tree was significantly correlated with the number of strain-specific insertions for each IS (correlation coefficient = 0.88, R^2^ = 0.76, p = 0.0001; **S5A Fig**), indicating strong agreement between both measures of recent IS activity. The overall patterns of inferred gain/loss events were biologically plausible, with few instances of biologically unlikely scenarios such as re-insertion of the same IS at the same site following a loss event (see **S1 Text** for more details). The ancestral state reconstruction analysis showed strikingly parallel IS-through-time trajectories for *S*. *sonnei* lineages II and III (**Fig 2E**). Lineage I genomes carried fewer IS insertion sites than lineages II and III (median 243 (IQR 217–251) unique insertion sites per chromosome vs 299 (286–312) and 294 (289–298) respectively; see **Fig 2C**). However the ancestral state reconstruction analysis indicated a very similar IS accumulation rate in all three lineages, albeit starting from a lower copy number in the lineage I MRCA (**Fig 2E**). Based on further investigation of the IS that distinguish lineage I from II and III, we propose this substantial gap in IS load most likely arose through a rapid expansion of IS*1* on the branch leading to the MRCA of lineages II and III, accumulating ~45 new IS*1* insertions over ~35 years, or 1.3 IS per year; see Methods**, S1 Text, S6 Table**.

Despite being distinguished by nucleotide divergence levels similar to those of *S*. *sonnei* lineages (**Fig 3A**), *S*. *dysenteriae* lineages were not differentiated in terms of the number of IS insertion sites identified by ISMapper (**Fig 2A**) and shared most of the same IS sites (mean 82% shared both within and between lineages; see **Fig 3B and 3C** and **S1 Fig**). Ancestral state reconstruction and lack of a strong association between IS insertion counts and year of isolation (R^2^ = 0.07, p = 0.012) further supports that IS have been relatively stable in the *S*. *dysenteriae* Sd1 genome for the century since the extant lineages diverged (**S6 Fig**), although lineages could still be distinguished based on IS profiles (see inset in **Fig 2A**).

*S*. *flexneri* lineages were 4–5 times more divergent from one another at the nucleotide level than were lineages of *S*. *sonnei* and *S*. *dysenteriae* (**Fig 3A**), and accordingly IS insertion counts and sites varied much more extensively between *S*. *flexneri* lineages as expected given the much greater amount of evolutionary time separating them (**Fig 2B, Fig 3B and 3C, S2 Fig**). Notably, pairs of isolates from different *S*. *flexneri* lineages still shared on average a third of their IS insertion sites (**Fig 3B**), indicating that a substantial IS expansion had occurred in the MRCA of *S*. *flexneri* prior to their diversification hundreds of years ago.

### Parallel expansions of common IS

**Fig 4:**
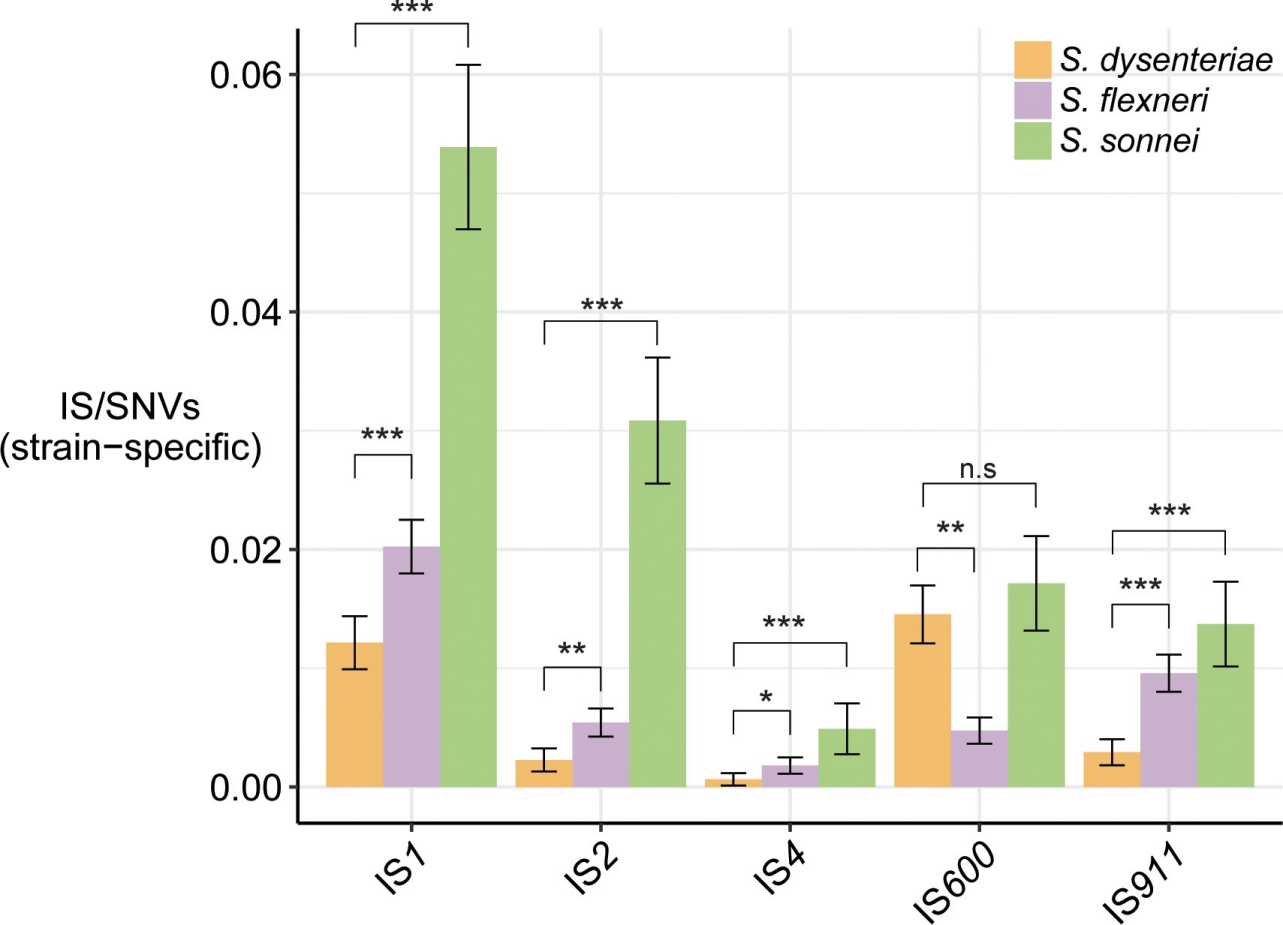
Activity of five common IS in each *Shigella* species. Bars indicate the ratio of strain-specific IS sites (estimated by ISMapper) to strain-specific SNVs, for each of the five common IS in each *Shigella* species population dataset. Error bars indicate 95% confidence intervals for the mean ratio across all terminal branches. Significant differences between species are indicated by asterisks, * indicates p < 0.05, *** indicates p < 10^−4^.

**Fig 5:**
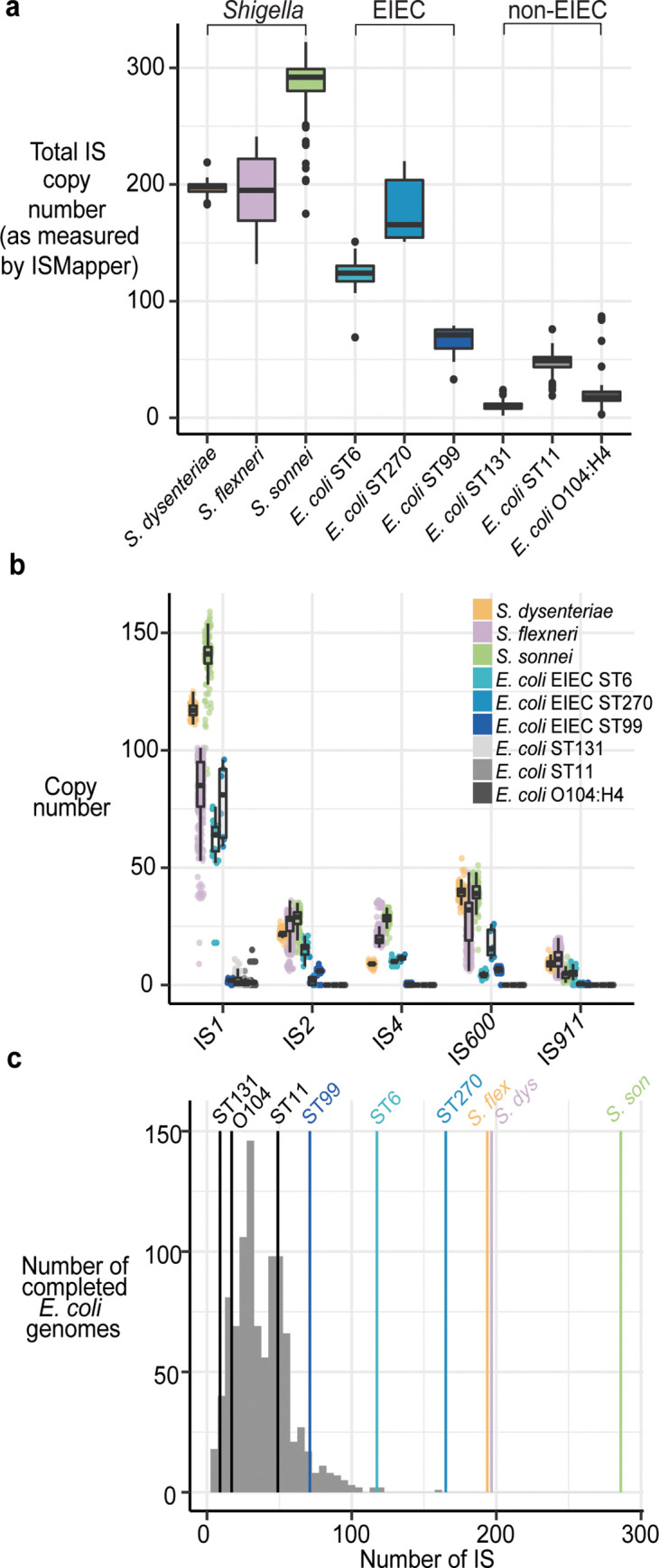
Comparison of IS burden in *Shigella* species and *E*. *coli*. **a,** Boxplots showing the distribution of number of IS in each *Shigella* species and the six pathogenic *E*. *coli* lineages, using ISMapper. **b,** Boxplots showing the distribution of number of unique insertion sites for each of the five common IS in each *Shigella* species and the six pathogenic *E*. *coli* lineages, estimated using ISMapper. **c,** Histogram of the total number of IS (as estimated by BLAST) for the non-redundant set of *E*. *coli* shown in **Fig 1A**. Coloured lines indicate median number of IS found in each *Shigella* species and the six pathogenic *E*. *coli* lineages (as measured by ISMapper).

The five common IS (IS*1*, IS*2*, IS*4*, IS*600*, IS*911*) accounted for most IS insertions detected in the *Shigella* reference genomes (including *S*. *boydii* Sb227 (clade C1/S1 in **Fig 1A**), see **Fig 1B** and **S1 Table**) and short-read population surveys (**Fig 2**), together comprising 99% of the IS burden in *S*. *dysenteriae*, 86% in *S*. *flexneri* and 85% in *S*. *sonnei*. IS*1* contributed most to the number of IS insertion sites in all three species (median 42–59% of insertions across all genomes within each species as detected by ISMapper, see **Fig 5B**; 46–73% of insertions identified in reference genomes, see **Fig 1B** and **S1 Table**). To assess the recent activity of these common IS in the various *Shigella* populations, we counted the number of strain-specific IS insertions and normalised these counts against the number of strain-specific SNVs (which is closely correlated with evolutionary time (R^2^ = 0.83) due to a strong molecular clock; see reference [33]). According to this measure, *S*. *sonnei* had significantly greater recent activity for each IS (p < 0.0003 in all cases; **Fig 4**). *S*. *dysenteriae* showed the lowest levels of recent activity for each IS except IS*600*, whose activity in *S*. *dysenteriae* was almost as high as in *S*. *sonnei* (**Fig 4**). The same five IS are present in the broader *E*. *coli* population, but at ~14-fold lower copy number per chromosome (median 15 in the non-redundant set of *E*. *coli* chromosomes; see **Fig 1** and **S7 Table**). These results confirm that the same five IS have undergone parallel expansion in all four *Shigella* species, and are still active in the *S*. *sonnei*, *S*. *flexneri* and *S*. *dysenteriae* populations under study.

To understand the expansions we observed in *Shigella* in the context of other pathogenic *E*. *coli* lineages, we examined six well-known human pathogenic lineages: (i) 99 genomes of the globally disseminated ST131, associated with drug resistant extra-intestinal infections [34–38]; (ii) 36 genomes of the O104:H4 Shiga toxin-producing enteroaggregative *E*. *coli* ST678, associated with a massive foodborne outbreak in Germany [39]; (iii) 199 genomes of O157:H7 enterohemorrhagic *E*. *coli* ST11, associated with frequent foodborne outbreaks globally [40]; and three different lineages of enteroinvasive *E*. *coli* (EIEC) from Public Health England: (iv) ST6 (n = 28), (v) ST99 (n = 24), and (vi) ST270 (n = 8) [41] (genome accessions listed in **S8 Table**). All genomes from the three EIEC lineages were confirmed to carry a variant of the virulence plasmid, the hallmark of the EIEC pathotype.

The median IS insertion site counts in these lineages (estimated using ISMapper) were 9, 17 and 49 (non-EIEC), and 118, 71 and 164 (EIEC); with the exception of EIEC ST270 (n = 164), these are substantially lower than total IS copies estimated with the same method in the *Shigella* populations (median 286, 194, 197; see **Fig 5A**). Notably all three EIEC pathotypes had higher IS copy numbers than the three non-EIEC pathotypes, providing more evidence of IS expansion in these lineages. We also compared IS copy numbers identified from completed *E*. *coli* chromosome sequences available in PATRIC, which confirmed that the three non-EIEC pathotypes had IS copy numbers within the typical range of IS counts for *E*. *coli* generally (median 32, IQR 22–57), whereas EIEC ST6, ST270 and *Shigella* chromosomes were outliers **Fig 5C**).

IS*1* was present in all of the pathogenic *E*. *coli* lineages but was expanded only in EIEC lineages ST6 and ST270 (median 0–2 in other lineages, see **Fig 5B**). IS*2* was found in all EIEC lineages (median 16 in ST6, 6 in ST99, 2 in ST270) and in a single copy in ST131. IS*4*, IS*600* and IS*911* were found in EIEC lineages but absent from non-EIEC lineages (**S1 Table**). Interestingly, the pathogenic *E*. *coli* lineages showed some signs of expansion of different IS not present in *Shigella*: IS*Ec12* and IS*Ec23* in ST131 (median n = 5), IS*Ec8* and IS*1203* in ST11 (median n = 14 and n = 18, respectively), IS*Ec23* in O104:H4 (median n = 5), and IS*621* in EIEC ST99 (median n = 24).

We hypothesised that the parallel IS expansions observed in *Shigella* chromosomes might be associated with introduction of IS variants via pINV. The presence of the same five IS in the chromosomes of pINV-carrying EIEC lineages would be consistent with this. However IS*1* is native to *E*. *coli* [42], which almost universally carry IS*1* in the chromosome, and phylogenetic analyses of IS*1* sequences suggest that each *Shigella* species has undergone independent proliferation of its resident *E*. *coli* chromosomal IS*1* variant (**S7A Fig**). The other four IS were common but not universal in *E*. *coli* genomes (present in 50–69% of the completed genomes). *Shigella* genomes shared a subpopulation of IS*2* and IS*4* variants distinct from those found in *E*. *coli* chromosomes (**S7B and S7C Fig**), consistent with transfer of IS*2* and IS*4* between *Shigella* species possibly via pINV, whose IS*2* and IS*4* sequences were intermingled with chromosomal sequences in the phylogenies. Notably the IS*2* and IS*4* alleles found in EIEC clustered with the *Shigella* alleles, consistent with introduction via pINV. IS*600* and IS*911* showed similar patterns but with more intermingling between *Shigella* and other *E*. *coli*, suggesting more widespread transfer mechanisms other than pINV (**S7D and S7E Fig**); consistent with this, IS*911* is lacking from pINV of *S*. *dysenteriae* and *S*. *sonnei* and IS*600* is lacking from pINV of *S*. *boydii* Sb227 (**S2 Table**).

### IS and genome degradation

All *Shigella* reference genomes had ≥199 pseudogenes (CDS inactivated by IS or other mutations), comprising ≥5% of CDS in each genome (**S1 Table**). Across the whole collection of *S*. *dysenteriae* (the species with the most-reduced coding capacity), 672 (15%) CDS were inactivated in at least one genome (by either an IS, a nonsense mutation, or a frameshift causing indel); in *S*. *sonnei* and *S*. *flexneri*, the numbers were 719 (14%) and 1,545 (30%) CDS, respectively. These natural gene-knockouts (pseudogenes) create strain-to-strain variation in coding capacity within each species, some of which shows evidence of fixation within lineages (median 103, 91 and 116 pseudogenes shared between within-lineage pairs of *S*. *dysenteriae*, *S*. *sonnei* or *S*. *flexneri*, respectively; **S8A Fig**), and some of which varies within lineages (median 37, 40 and 53 non-shared pseudogenes between pairs, respectively; **S8B Fig**).

**Fig 6:**
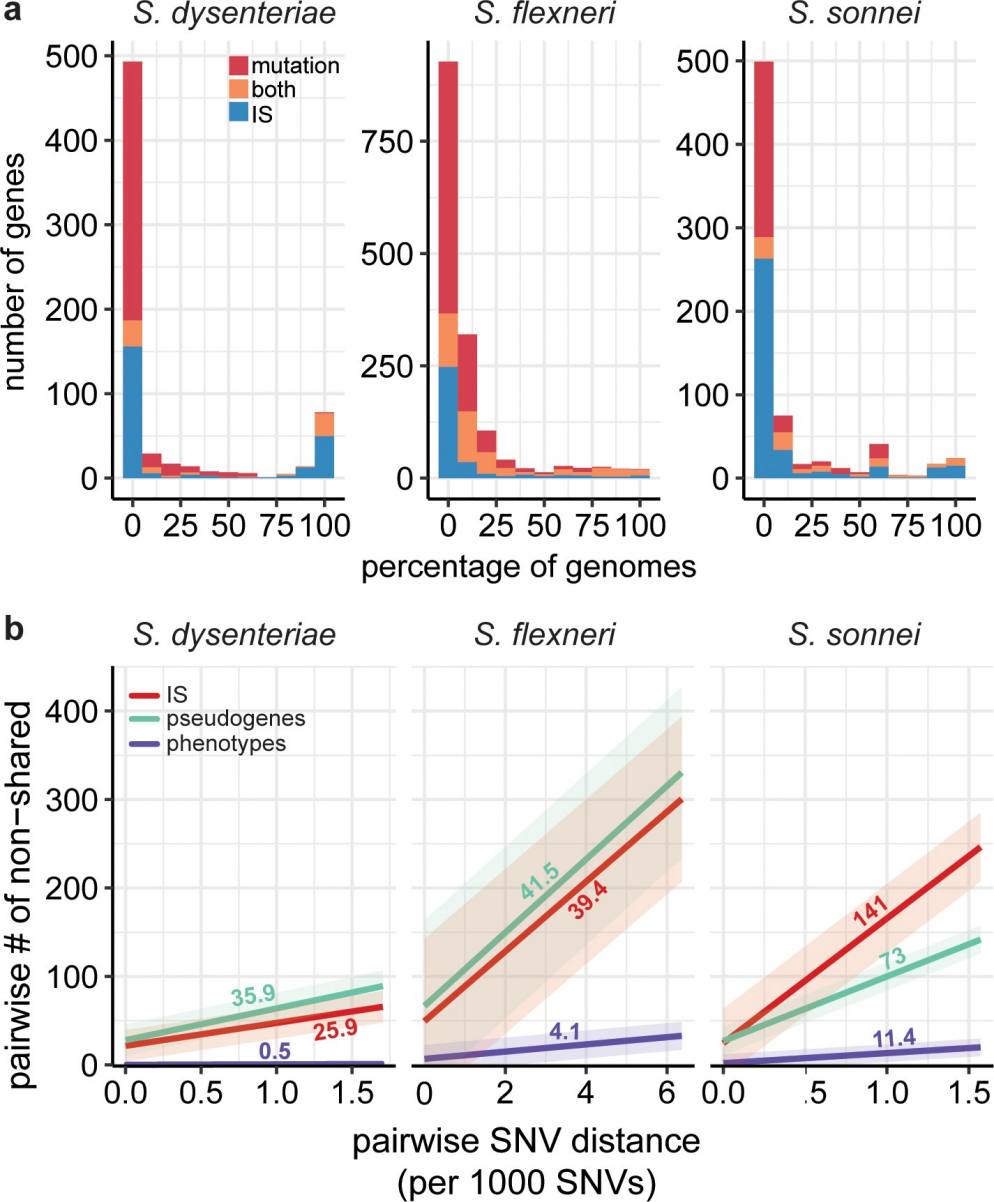
Patterns of IS, pseudogene and metabolic diversification within each *Shigella* species. **a,** Histogram summarises the population prevalence of gene interruptions detected in each *Shigella* species, coloured to indicate the mechanism/s of interruption as per inset legend. **b,** Linear regressions of pairwise counts of non-shared IS (red), pseudogenes (green) and metabolic phenotypes (purple) against pairwise SNV distance, for each *Shigella* species. Fitted lines are labelled with their slope. The full data to which each line was fit can be found in **S10 Fig**. All correlations were significant (p<0.001) using Mantel test to compare the pairwise distance matrices.

The proportion of pseudogenes that were directly attributable to IS insertion was 50% in *S*. *sonnei*, 22% in *S*. *flexneri* and 35% in *S*. *dysenteriae* (**S9A Fig**). A further 12%, 24% and 11%, respectively, of interrupted genes harboured both IS and other inactivating mutations, thus could reflect initial inactivation by one mechanism followed by further degradation by another (**S9A Fig**). Notably, in all species the vast majority of conserved pseudogenes (i.e. those inactivated in ≥95% of genomes) were interrupted by IS (24/24 in *S*. *sonnei*, 19/20 in *S*. *flexneri*, 77/78 in *S*. *dysenteriae*). In *S*. *dysenteriae* and *S*. *sonnei* most of these lacked any other inactivating mutations, suggesting the IS insertion was the inactivating event (**Fig 6A**). Within each species, much of the strain-to-strain variation in pseudogene content was IS-driven, both within and between lineages (median 35–60% of pairwise differences; see blue and grey boxes in **S9B–S9D Fig**). To quantify and compare the diversification of IS and pseudogene profiles over time within species, we modelled pairwise counts of non-shared IS insertions and non-shared pseudogenes as a linear function of pairwise SNVs (**Fig 6B**). Strong linear relationships were evident in all cases (p<0.001 using Mantel test, see **S10 Fig**). The results indicate that diversification of both IS and pseudogene profiles is occurring most rapidly in *S*. *sonnei* (mean 141 IS and 73 pseudogenes per 1,000 SNVs), whereas ongoing diversification of both types has slowed in *S*. *dysenteriae* (25.9 IS and 35.9 pseudogenes per 1,000 SNVs) (**Fig 4B**). Note that the reported substitution rates for these species are very similar (6.0x10^-7^ per site per year for *S*. *sonnei* [27], 8.7x10^-7^ for *S*. *dysenteriae* [26], and between 6.4x10^-7^ (lineage 1) and 9.5x10^-7^ (lineage 2) for *S*. *flexneri* [25]), hence these IS and pseudogene rates normalised to SNV counts are also comparable as clock rates.

Taken together, our results indicate that *S*. *sonnei*, *S*. *flexneri* and *S*. *dysenteriae* have undergone similar patterns of IS expansion and genome degradation; and that *S*. *dysenteriae* is the furthest along this evolutionary trajectory, with the greatest number of IS copies and the most reduced genome but also the lowest current IS activity or copy number expansion, which is consistent with the end-point of IS saturation as predicted by published models of IS expansion [43]. Next we consider the functional impacts of IS and genome degradation, and the evidence that these mechanisms contribute to convergence towards functionally-similar streamlined genomes that are adapted to the human-restricted niche associated with *Shigella*. As *S*. *dysenteriae* is the furthest along the evolutionary trajectory away from other *E*. *coli*, we compare functional loss in the other *Shigella* species with that observed in *S*. *dysenteriae*.

### IS and convergent gene loss

The ratio of IS insertions per base for genic vs intergenic regions was well below one in all three species (0.20 in *S*. *flexneri*, 0.29 in *S*. *sonnei*, 0.11 in *S*. *dysenteriae*), suggesting an overall pattern of purifying selection against insertions within CDS for each species. There was also evidence of parallel loss of the same genes in *S*. *sonnei* or *S*. *flexneri* compared to the most-reduced species *S*. *dysenteriae* (**S9 Table**), indicative of convergent evolution. We compared the number of pseudogenes in *S*. *sonnei* and *S*. *flexneri* that were either fixed pseudogenes (inactive in ≥90% of *S*. *dysenteriae* genomes), or completely missing from *S*. *dysenteriae* and assessed significance using a permutation test (see **Methods**). In *S*. *sonnei*, 61% (440/719) of the pseudogenes identified in any genome were also either fixed pseudogenes (n = 12/719, 2%, p = 0.013) or missing (n = 428/719, 59%, p<0.001) in *S*. *dysenteriae* (see **S11 Fig**). Of the 428 that were missing in *S*. *dysenteriae*, 334 (78%) were conserved in ≥90% of *E*. *coli* genomes, suggesting they were likely present in an ancestor of *S*. *dysenteriae* but have been deleted during genome degradation. Similarly, 54% (n = 832/1545) of *S*. *flexneri* pseudogenes were either inactivated (n = 23/1545, 2%, p = 0.001) or absent (n = 809/1545, 52%, p<0.001) in *S*. *dysenteriae* (see **S11 Fig**). Again, 576 (71%) of the 809 *S*. *flexneri* pseudogenes that were missing in *S*. *dysenteriae* were conserved in ≥90% of *E*. *coli*, consistent with their deletion from an *S*. *dysenteriae* ancestor. A similar pattern was observed when considering the most common *S*. *flexneri* lineages 1 and 3 separately (**S11 Fig**). Taken together, these results indicate that the degree of parallel functional gene loss in *S*. *sonnei* and *S*. *flexneri* compared to *S*. *dysenteriae* exceeds that due to random chance and thus is indicative of selection.

There was no significant functional enrichment amongst the genes lost in parallel between species (see **Methods**); however in each species the largest functional class assigned to pseudogenes was carbohydrate metabolism (**S12 Fig**), and in each species, 37–43% of pseudogenes with known function were metabolism related.

### IS and convergent metabolic reduction

**Fig 7:**
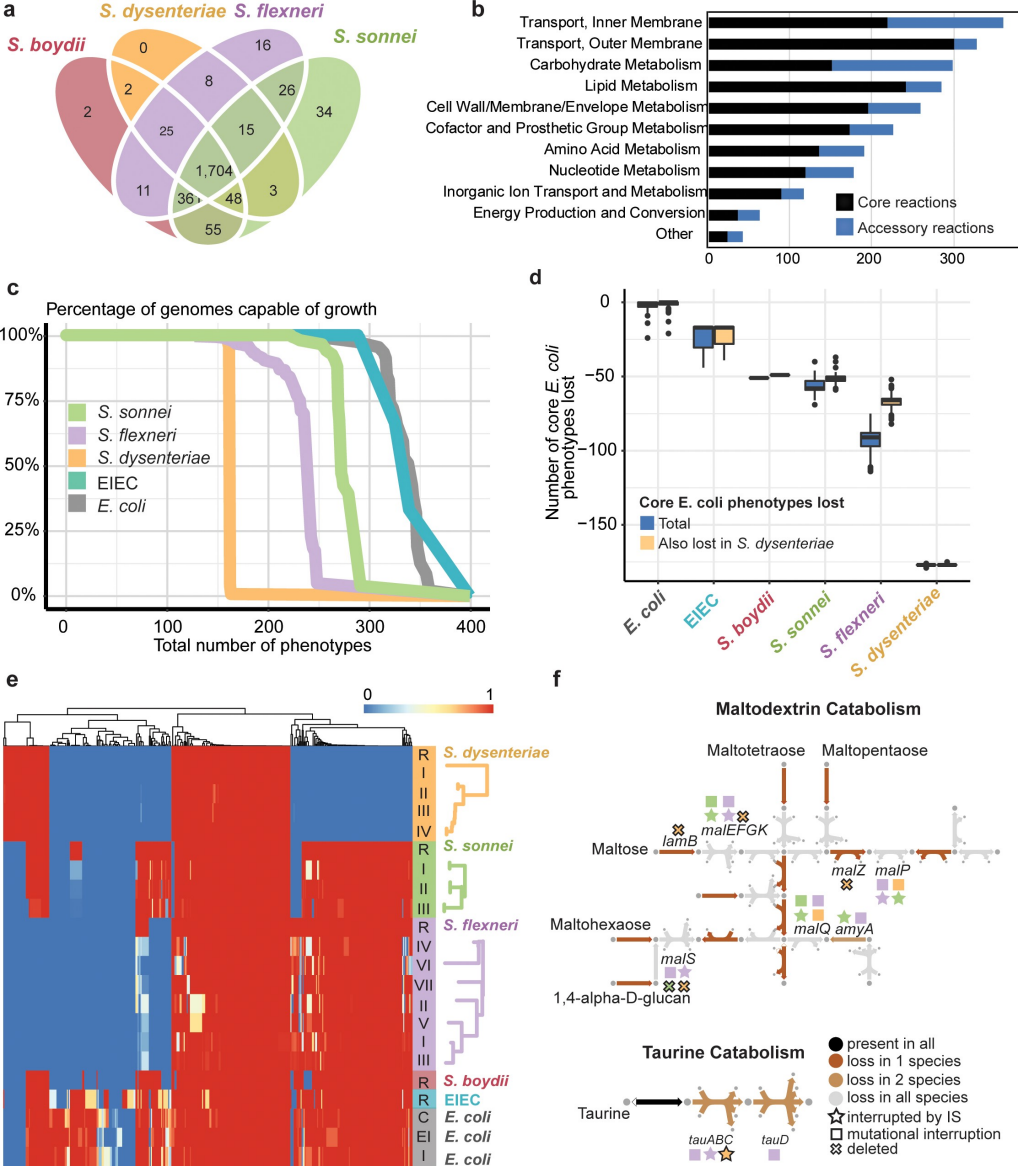
Genome-scale modelling illustrates convergent evolution of *Shigella* species. **a,** Shared and strain-specific metabolic reactions present in GEMS constructed for hypothetical IS-free reference sequences of four selected *Shigella* species. **b,** Shared and strain-specific reactions are distributed across metabolic subsystems with the most strain-specific reactions found in inner membrane transport and carbohydrate metabolism. **c,** Percentage of *Shigella*, EIEC and *E*. *coli* isolates predicted to be capable of growth on 386 different growth supporting nutrients. x-axis is a list of substrates order by their percentage growth across all species. **d,** Blue boxplots show the number of metabolic phenotypes (growth capabilities) lost in each *E*. *coli*, EIEC and *Shigella* strain, compared to the core metabolic capability of *E*. *coli* (n = 316 substrates). Yellow boxplots indicate the overlap between these lost phenotypes and the set of 178 core *E*. *coli* phenotypes lost by *S*. *dysenteriae*. **e,** Heatmap of model-predicted growth capabilities for subsets of *Shigella* strains, including IS-free reference genomes (R) and lineages within each species (lineages defined and ordered by tree structure as illustrated on left and in **Fig 2**); three EIEC reference genomes (from **Fig 1A**, all grouped together); and 47 *E*. *coli* genomes, grouped by disease type (C–commensal; EI–extra-intestinal; I–intestinal). Proportions of isolates in each group capable of growth on each nutrient source are coloured according to legend. **f,** Summary of convergent degradation in taurine (sulfur) and maltodextrin (carbon) metabolism pathways. Arrows indicate reactions present in the intact *E*. *coli* pathways and are coloured by their frequency of loss in the three main *Shigella* species as per inset legend. Gene inactivation events resulting in loss of reactions are indicated with symbols above or below the arrow; shapes indicate the genetic mechanism (IS, mutation or deletion, see inset legend), colours indicate the species (as per panel c), black-outlined shapes indicate mutations that are fixed within the species.

We used genome-scale models of metabolism (GEMs) [44,45] to simulate growth capabilities for the various *Shigella* populations *in silico* [46]. GEMs for the IS-free reference genomes of all four *Shigella* species were built with reference to a recent *E*. *coli* GEM [47] (see **Methods**, **S10 Table & S11 Table**). A total of 1,704 reactions were shared by all four *Shigella* reference GEMs and a further 604 reactions were found in at least one (**Fig 7A and 7B**). From these we built strain-specific GEMs for all *S*. *sonnei*, *S*. *flexneri*, and *S*. *dysenteriae* genomes, by removing from the species reference model reactions that were predicted to be interrupted in that genome (~20% of all pseudogenes were components of GEMs, see **S9A Fig**; 32–47% of these harbour IS). We used the resulting GEMs to predict the carbon, nitrogen, phosphorous and sulphur substrates that could support growth of each isolate (n = 386 substrates tested, referred to hereafter as predicted growth capabilities; see **S13–S17 Figs**). Metabolic maps showing conservation of each reaction pathway can be found in **S18–S20 Figs**.

This analysis confirmed *S*. *dysenteriae* to be the most metabolically limited of all four *Shigella* species, with less than two-thirds the predicted growth capabilities of the others (median 161 per strain) and little evidence of variation between strains (**S13 Fig**): all of the *S*. *dysenteriae* isolates were predicted to be capable of growth on the 161 substrates in the ancestral model (**Fig 7C**). *S*. *sonnei* had the greatest number of predicted growth capabilities (median 270 per isolate) and some strain variation (median 12 pairwise differences), whereas *S*. *flexneri* strains showed an intermediate number of predicted growth capabilities (median 230 per strain) with some variation between strains (median 20 pairwise differences; see **Fig 7C, S13 Fig**). Notably, pairwise differences in predicted growth phenotypes of strains of *S*. *sonnei* and *S*. *flexneri* accumulated in a linear fashion compared to SNVs, at mean rates of 11.4 and 4.1 per 1,000 SNVs, respectively (**Fig 6B, S10 Fig**); in contrast *S*. *dysenteriae* appears to have settled in a static reduced metabolic state, with occasional transient (non-fixed) further loss of phenotypes (**Fig 6B**, **Fig 7C and 7E**).

To explore convergence of metabolic phenotypes in *Shigella* species, we compared the *Shigella* GEMs with those reported previously for 47 diverse *E*. *coli* strains [44] as well as GEMs we built for the three EIEC reference genomes (**S12 Table**). The 47 *E*. *coli* GEMs shared a set of 316 core predicted growth capabilities (common to 90% of the strains), accounting for median 95% of capabilities per strain. Extensive loss of these core *E*. *coli* capabilities was apparent in each *Shigella* species, ranging from median 51 and 58 (16% and 18%) in *S*. *boydii* Sb227 and *S*. *sonnei*, to median 91 (29%) in *S*. *flexneri* and 177 (56%) in *S*. *dysenteriae* (blue in **Fig 7D**). Notably, the majority of the core *E*. *coli* metabolic capabilities lacking from *S*. *boydii* Sb227, *S*. *sonnei* or *S*. *flexneri* overlapped with those lacking in the most-reduced species *S*. *dysenteriae* (median 96%, 78%, 60%, respectively), consistent with convergent metabolic reduction across *Shigella* species (**Fig 7D and 7E**). To assess the significance of this apparent convergence towards the reduced metabolic capacity of *S*. *dysenteriae*, we permuted null distributions of core *E*. *coli* phenotype loss vs observed phenotype loss in *S*. *dysenteriae* (**S21 Fig**). For *S*. *sonnei*, *S*. *boydii* Sb227, *S*. *flexneri* lineage 1 and *S*. *flexneri* lineage 3, the core phenotypes lost overlapped with those lost in *S*. *dysenteriae* much more than expected under a model of random loss of core *E*. *coli* phenotypes (p<0.001 using permutation test). The three EIEC lineages also showed some loss of core *E*. *coli* capabilities (median 14, 4%); 89% of these were also lost from *S*. *dysenteriae* (significant overlap, p = <0.001 using permutation test) and 99% from either *S*. *flexneri* or *S*. *sonnei*, consistent with these pINV-carrying lineages being at an earlier point on a similar evolutionary trajectory to *Shigella* (**Fig 7D and 7E**).

This convergent loss of metabolic pathways was attributable to independent genetic lesions in each *Shigella* species, including a mix of IS and other mutations occurring in the same or different genes, often with degradation of multiple genes in the same pathway (examples of convergent loss of taurine as a sulfur source, and of maltodextrins as a carbon source, are shown in **Fig 7F**).

To address the impact of IS on pathway loss in the three *Shigella* species populations, we identified all inactivating events (either IS or mutational) affecting each metabolic reaction in each species. For each species, we identified the most frequent mutation event affecting each reaction, reasoning that the highest frequency event is the most conserved and thus the earliest inactivating event. We further classified the earliest inactivating event affecting each reaction as either ancient (conserved in >80% of genomes in that species), intermediate (20–80%), or recent (<20%) (**S22 Fig**). In *S*. *sonnei and S*. *dysenteriae*, all ancient inactivating events (>80% of genomes) within metabolic reactions were IS insertions (**S22 Fig**). In *S*. *flexneri*, 30% of ancient inactivating events in metabolic reactions were IS, but notably all completely conserved mutations (n = 3) were IS insertions. This supports our hypothesis that IS have significantly contributed to pathway loss in each species.

Only 109 metabolic phenotypes were common to all four *Shigella* species (present in *S*. *boydii* Sb227 and >90% of genomes from each of the three target species; see **S13 Table**). These phenotypes are all part of the 316 core *E*. *coli* capabilities defined above, and may constitute the minimum metabolic requirements for *Shigella* (**S13–S17 Figs**). This core set of *Shigella* growth capabilities includes 19 carbon, 30 nitrogen, 3 phosphorous and 6 sulfur sources. It accounted for mean 67% of growth capabilities identified in each *S*. *dysenteriae* strain, but only mean 47% of those in *S*. *flexneri* strains and mean 40% in *S*. *sonnei*, suggesting the latter species could continue on the trajectory of metabolic loss for some time.

## Discussion

Overall our data show that IS have had a substantial impact on the evolutionary history of the three major *Shigella* species clades and continue to shape the ongoing diversification and evolutionary trajectories of *S*. *sonnei* and *S*. *flexneri*. Despite the fact that the genome collection for each species group was relatively small (~120 for *S*. *sonnei* and *S*. *dysenteriae*, 343 for *S*. *flexneri*), we believe that these genomes are representative of each population. All three of the genome collections were selected using a similar method–each are a representative snapshot from historical collections that captured diversity across both time and space. The collections represent 65 (*S*. *sonnei*), 96 (*S*. *dysenteriae*) and 98 (*S*. *flexneri*) years of isolation and have genome representatives from at least four continents. In the case of *S*. *dysenteriae*, additional effort was made to ensure that there were representatives of all recorded serotype 1 outbreaks for which isolates could be obtained [26].

All three major *Shigella* species clades examined in this study were found to have significantly higher IS copy numbers and reduced genomes compared to their *E*. *coli* relatives, consistent with previous reports [15]. Notably our data indicate highly parallel expansions of the same five IS types at similar relative levels in these three *Shigella* species (**Figs 2 and 5**) and *S*. *boydii* Sb227 (**Fig 1**); in contrast we found no evidence of a similar IS expansion in most other *E*. *coli*, including known pathogenic lineages. The exceptions were the EIEC lineages, which carried the same five IS and showed expansion of some of these, although to a lesser degree than in *Shigella* (IS*1*, IS*2*, IS*4*, IS*911* in ST66; IS*2*, and IS*600* in ST99; IS*1*, IS*4*, IS*600* in ST270; see **Fig 5B**). EIEC are characterised by the presence of the *Shigella* invasion plasmid pINV, which provides them a similar ability to invade the human intestinal epithelium to cause severe diarrhea. All *E*. *coli* carry IS*1*, and our data suggests that each Shigella and EIEC lineage underwent expansion of their resident IS*1* alleles; however IS*2*, IS*4*, IS*600* and IS*911* are much rarer in the *E*. *coli* population and our data are consistent with the hypothesis that pINV is a potential source for the introduction of these IS into *Shigella* and EIEC lineages.

The expansion of IS within *Shigella* has accompanied large scale genome reduction and convergent evolution towards a similar phenotype profile, in line with expectations about genetic bottlenecks related to host adaptation of bacterial pathogens in general and *Shigella* species in particular [15,18]. However, this study is unique in identifying the dynamics of IS expansion and genome degradation within *Shigella* species as they continue to diversify and evolve under selection (**Figs 2, 3, 6 and 7**). Particularly striking were the multiple lines of evidence indicating that IS activity is contributing to ongoing diversification of *S*. *sonnei* and *S*. *flexneri*, and that each shows convergence towards a streamlined genome that is similar to the highly-reduced *S*. *dysenteriae* (**Fig 7**), via both parallel evolution (through the inactivation of genes homologous to those lost from *S*. *dysenteriae*) and convergent evolution (whereby the same functional pathways are disrupted in each species but through different mechanisms). The accuracy of the GEMS used here to predict growth capabilities have been previously estimated at 80%. Unfortunately the isolates included in the genome collections under study (chosen for their diverse representative sampling, as discussed above) were not available to us hence we could not confirm individual metabolic phenotype predictions in individual strains. However, as our analyses focus on comparisons between phylogenetic groups of genomes modelled using the same underlying *E*. *coli* reactome model, inaccuracies in that underlying model cannot explain the observed similarities between *Shigella* clades relative to other *E*. *coli*. Furthermore, our conclusions do not rest on any single growth capability but rather the overall patterns of predicted phenotypes compared within and between clades, and confirming predictions for a small number of substrates would have little impact on the overall conclusions. Notably, the convergent metabolic losses identified here provide a starting point to guide future experimental work focussed on functional questions, such as whether convergent metabolic slimlining has any functional impact on host adaptation and/or pathogenicity in *Shigella*.

Notably, our analysis predicts a remarkable degree of strain-to-strain functional variation within the *S*. *sonnei* and *S*. *flexneri* species clades, which could have implications for experimental studies of *Shigella* pathogenicity, host interactions and antimicrobial resistance that typically rely on a limited number of lab strains [48]. For example, the *S*. *sonnei* lab strain 53G (lineage II) differed from lineage III isolates (which are responsible for the majority of *S*. *sonnei* case isolates sequenced worldwide in the past decade [27,29,49,50]) by median 24 predicted substrate growth capabilities (IQR, 23–25). In general, lineage II *S*. *sonnei* isolates differ from lineage III isolates by median 97 protein-coding genes (IQR 93–101). For *S*. *flexneri*, the common lab strains are serotype 5a strain M90T (lineage 5 [9]) and serotype 2a strains 301 or 2457T (lineage 3 [51]; note strain 301 was used in the present study as a reference genome). *S*. *flexneri* serotype 2a/lineage 3 are common amongst clinical isolates [24,52], however recent genomes of lineage 3 clinical isolates are quite diverged from 301 –in our analysis lineage 3 strains differed pairwise by median 45 protein-coding genes (IQR 37–55) and 59 predicted substrate growth capabilities (IQR 37–55). *S*. *flexneri* serotype 5a/lineage 5 is rare (not detected at all in GEMS [24]), and we estimate lineage 5 strains such as M90T differ pairwise from the clinically dominant lineage 3 by median 139 protein-coding genes (IQR 134–144) and 62 (IQR 56–63) substrates.

The available evidence suggests that much of the convergent loss of metabolic function is due to IS activity in the evolutionary histories of each the various *Shigella* species (**Figs 6 and 7F**). Firstly, in all *Shigella* species, nearly all conserved pseudogenes (i.e. genes that have been inactivated for some time but not yet deleted from the genome) were disrupted by IS (**Fig 6A**). Secondly, fixed deletions accounted for 33–51% of degradation in the *Shigella* reference GEMS compared to *E*. *coli* GEMS. Whilst it is difficult to attribute past deletion of specific genes to IS activity, it is likely that a significant proportion of overall gene loss in the IS-laden *Shigella* genomes has been IS-mediated. Thirdly, IS disruption accounted for a large proportion (32–47%) of all within-species variation in metabolic genes included in the *Shigella* GEMS, indicating that IS continue to play a central role in ongoing *Shigella* evolution.

IS are known to have played a key role in host adaptation and genome degradation in many other pathogens, including *Bordatella pertussis* [14],*Yersinia pestis* [46], *Mycobacterium leprae* [13] and *Mycobacterium ulcerans* [53]. There have been previous studies that examine the impact of IS on the evolutionary trajectories of bacterial pathogens, including *Mycobacterium tuberculosis* [54], *Acinetobacter baumannii* [55,56], *Klebsiella pneumoniae* [56] and *Burkholderia cenocepacia* [57]. However, these studies relied on a small number of genome representatives, captured shorter evolutionary timescales, did not use genome-scale metabolic modelling to systematically explore the functional impact of IS activity, and did not attempt systematic comparisons between multiple species or lineages. The present study provides a novel framework to examine with greater scrutiny the impact and dynamics of IS in other bacterial pathogens on a large scale, and to assess evidence for their role in convergent evolution. In particular we have shown how ISMapper can be applied to derive novel insights into IS variation and evolutionary dynamics using existing high-throughput short-read data, and that genome-scale metabolic modelling can be harnessed to help unravel convergent evolution at the pathway level. Notably, a recent study examining the impact of IS on the transcriptome of *B*. *pertussis* showed that many IS insertions modified the expression of neighbouring genes, creating strain-specific differences in gene regulation [23]. This approach could be combined with ours to establish an even more detailed understanding of the role of IS in pathogen evolution.

## Methods

### Detection of IS in *Shigella* and *E*. *coli* reference genomes

IS were detected in each of the reference genomes included in **Fig 1** using ISSaga [58]. ISSaga screens the genome for known homologs of all IS sequences contained in the ISFinder [59] database. IS which had at least 80% nucleotide identity to an IS in the ISFinder database and that were present in at least one complete copy were included. Reference sequences for each of the detected IS were downloaded from the ISFinder database, and screened against each reference genome using BLAST+ v2.6.0 [60]. Nucleotide hits with ≥95% identity and ≥99% coverage were counted in the IS copy number tally for each reference chromosome (**S1 Table**) and plasmid (**S2 Table**).

In order to calculate the IS burden in the general *E*. *coli* population, all *E*. *coli* genomes marked as ‘completed’ in PATRIC [61] (as at January 8, 2020, n = 1943) were downloaded. This set of genomes was then de-replicated using the Assembly Dereplicator tool (https://github.com/rrwick/Assembly-Dereplicator) with a threshold of 0.005. From the set of de-replicated genomes, 100 genomes were randomly selected for further analysis (**S14 Table**). BLAST+ v2.2.30 was used to screen each *E*. *coli* genome for IS, including the five common *Shigella*-expanded IS (IS*1*, IS*2*, IS*4*, IS*600* and IS*911*). Nucleotide BLAST+ hits with ≥95% identity and ≥90% coverage were counted in each genome’s tally for each IS (**S7 Table**).

### Creation of IS-free *Shigella* reference genomes

To allow for more precise detection of IS insertion sites within genes, and provide a basis for constructing GEMS, IS-free versions of the chromosome sequences of *S*. *sonnei* 53G (accession NC_016822), *S*. *dysenteriae* Sd197 (accession NC_007606) and *S*. *flexneri* 2a strain 301 (accession AE005674) were created as follows. IS detected in the reference genomes using ISSaga and BLAST+ as described above were annotated, and manually inspected using the Artemis genome browser [62] to ensure that the IS sequence was complete and any target site duplications were included in the annotated feature. Each of the annotated features (i.e. IS plus target site duplication if present) was then deleted from the reference chromosome sequence. Annotations of CDS and gene features in the complete reference genome were then transferred to the IS-free reference chromosome sequence using RATT [63], with the strain transfer parameter. The IS-free reference chromosome sequences are deposited in FigShare (doi: 10.26188/5c7daac90d298).

### Completion of *E*. *coli* ST270 reference genome 8-3-Ti

As no complete reference genome for *E*. *coli* ST270 was available, we used a combination of short and long read sequencing to complete the genome of isolate 8-3-Ti (provided by Prof David Gordon, Australian National University). Genomic DNA was prepared from bacterial pellets using GenFind v3 reagents (Beckman Coulter). Illumina sequencing libraries were made with Nextera Flex reagents and the Illumina CD indexes as per manufacturer’s instructions, with one major deviation from described protocol–reactions were scaled down to 25% of recommended usage. Illumina libraries were sequenced on the NovaSeq platform using the 6000 S1 Reagent Kit (300 cycles). Resulting Illumina reads had a mean length of 149 bp, with an estimated depth of 107x. A long-read sequencing library for Oxford Nanopore Technologies (ONT) was prepared using the ligation library kit (LSK-109) with native barcoding expansion packs (EXP-NBD104 and NBD114), with sequencing performed as previously described [64]. Reads were basecalled using Guppy v3.4.4. N50 read length was 27,590 bp, with an estimated depth of 35x. A hybrid assembly was constructed using both the ONT and Illumina reads with Unicycler v0.4.8 (default settings) [65]. The resulting assembly had a total genome size of 5,141,082 bp and consisted of three replicons–the chromosome (4,899,710 bp), and two plasmids (233,800 bp (pINV) and 7,413 bp). The complete genome sequence was deposited in GenBank under accessions CP050865, CP050866 and CP050867.

### Detection of IS in *Shigella* and *E*. *coli* populations from short read data

The *S*. *sonnei* data comprised 132 isolates from the Holt *et*. *al* global study [27], sequenced on the Illumina Genome Analyzer GAII, generating paired end reads. Sequenced genomes had mean read length 59 bp and mean read depth 83x (range 68x - 91x); six genomes with low mean depth (<10x) were excluded from the analysis. The remaining 126 genomes were screened for the 12 IS identified in *S*. *sonnei* 53G by ISSaga (IS*1*, IS*2*, IS*4*, IS*21*, IS*600*, IS*609*, IS*630*, IS*911*, ISEc20, ISSso1, ISSso4, ISSso6) using ISMapper v1 [31] to identify the insertion sites of each IS relative to the IS-free *S*. *sonnei* 53G reference sequence (using typing mode and default parameters). Briefly, ISMapper maps paired short reads to each IS query sequence, and identifies unmapped partners of reads that mapped to the IS. The former demarcate DNA sequences that flank IS insertion sites, and are mapped to the reference genome sequence to determine the precise location and orientation of IS insertions captured in the short read data [31].

The *S*. *dysenteriae* data comprised 125 genomes from the Njamkepo *et*. *al* global study [26], with Illumina paired reads of 100–146 bp (mean read length 115 bp) and mean read depth 193x (range 312x - 2889x). Insertion sites for the six IS detected in the *S*. *dysenteriae* Sd197 complete reference genome (IS*1*, IS*2*, IS*4*, IS*600*, IS*911* and ISEc8) were identified in all 125 genomes using ISMapper v1 with the same settings described for *S*. *sonnei* but using the IS-free *S*. *dysenteriae* Sd197 reference sequence.

The *S*. *flexneri* data consisted of 343 genomes from the Connor *et*. *al* global study [25], sequenced via Illumina HiSeq with 100 bp paired end reads with mean read depth 102x (range 19x - 419x). Insertion sites for the twelve IS detected in the *S*. *flexneri* reference genomes (IS*1*, IS*2*, IS*4*, IS*600*, IS*609*, IS*911*, IS*1203*, IS*150*, ISEc17, ISEhe3, ISSlf3 and ISSfl4) were identified in each of the 343 genomes using ISMapper v1 with the same settings described for *S*. *sonnei* but using the IS-free *S*. *flexneri* 301 reference sequence.

To investigate IS expansion in other pathogenic lineages of *E*. *coli* (**Fig 5**), six different clonal lineages representing different pathotypes and STs of *E*. *coli* were analysed. ST131 uropathogenic *E*. *coli* (UPEC) (n = 99) and reference strain EC958 [58] (accession HG941718) were collated from multiple separate studies [34–38]. ST11 enterohemorrhagic *E*. *coli* (EHEC) genomes (n = 199) from the public GenomeTrackr were identified based on MLST data from Ingle *et*. *al* [40]. The ST11 reference genome was *E*. *coli* O157:H5 strain EDL933 [59] (accession NZ_CP008957). Representatives of the German outbreak clone O104:H4 (n = 36) [39] with reference *E*. *coli* strain C227-11 [60] (accession NC_018658) were obtained from NCBI. Three different enteroinvasive (EIEC) lineages, ST6 (n = 28), ST99 (n = 24) and ST270 (n = 8) were obtained from a single study [41]. ST6 genomes were compared to reference strain *E*. *coli* NCTC 9031 (https://www.sanger.ac.uk/resources/downloads/bacteria/nctc/); ST99 genomes to reference *E*. *coli* CFSAN029787 (accession CP011416); ST270 genomes to reference strain *E*. *coli* 8-3-Ti3 (completed for this study, accession CP050865). Accessions for all *E*. *coli* short-read data are listed in **S8 Table**. Read statistics were: ST131, mean length 100 bp, mean depth 60x (range 34x - 202x); ST11, mean length 170 bp, mean depth 80x (range 16x - 245x); O104:H4, mean length 98 bp, mean depth 57x (range 8x - 214x); ST6, mean length 100 bp, mean depth 37x (range 28x – 75x); ST99, mean length 100 bp, mean depth 55x (range 25x – 84x); ST270, mean length 100 bp, mean depth 40x (range 25x – 59x). IS were identified in each *E*. *coli* reference genome using ISSaga as described above. This revealed five IS in ST131, nine IS in ST11, thirteen IS in O104:H4, four in ST6, thirteen in ST99, and twelve in ST270 (see **S1 Table** for IS). For each *E*. *coli* lineage, the IS detected in their reference, and the five IS common to *Shigella*, were used as queries with ISMapper to identify the IS insertion sites in each genome of that lineage, using the same parameters as described for *Shigella*. (We note that the original reporting of IS copy number in these genomes differs from our analysis, due to differences in methods. Whilst Cowley *et al* [41] used ISMapper, they compared all three EIEC lineages to the *S*. *sonnei* Ss046 reference genome, which is likely to yield less accurate results than our analysis using lineage-specific references for each ST. Additionally, Cowley *et al* did not state which IS elements they examined, making a direct comparison impossible.)

### Detection of SNVs and pseudogenes in *Shigella*

All 125 *S*. *dysenteriae*, 343 *S*. *flexneri* and 126 *S*. *sonnei* genomes were mapped to their respective IS-free reference genomes using the RedDog pipeline v01.9b (www.github.com/katholt/RedDog) to detect SNVs and indels. Briefly, RedDog uses Bowtie2 v2.2.3 [66] with the sensitive-local parameter and a maximum insert size of 2000 bp to map all read sets to the reference genome. High quality SNV sites (homozygous calls supported by ≥10 reads, phred score ≥30, in at least one genome) were identified using SAMtools v0.0.19 [67], and high quality alleles at each SNV site determined across all genomes by extracting the consensus base from each genome using SAMtools pileup (low quality base calls–defined as phred quality ≤20, read depth ≤5, or a heterozygous base call– were set to the gap character to indicate an unknown allele).

We defined pseudogenes as genes that contained a nonsense mutation or a frameshift causing indel. To identify genes with nonsense mutations, the SNV consequences file from RedDog was used, which annotates each SNV with its coding effect (i.e. whether it is intergenic or genic; and for genic SNVs what the effect on the encoded protein is), based on the annotation of the coding features in the reference genome (in this case, the IS-free reference genomes). From this SNV consequences file, only SNVs generating nonsense mutations were extracted and kept for downstream gene inactivation analysis. Indel positions were extracted from the VCF files output by RedDog, and these indel positions were compared to the annotations in IS-free reference genomes to determine which indels were within genes. Only indel positions causing frameshifts within genes were kept for downstream pseudogene analysis.

### Phylogenetic inference

To construct phylogenies for each *Shigella* species clade, the alignments of SNV alleles produced by the RedDog analyses (described above) were each filtered to exclude SNVs falling in repeat regions or phage (detected using PHAST [68]). The resulting alignments were 10,798 SNVs in length for *S*. *dysenteriae*; 40,073 SNVs for *S*. *flexneri*; and 6,843 SNVs for *S*. *sonnei*. These alignments were used to construct either dated (*S*. *sonnei* and *S*. *dysenteriae*) or maximum likelihood phylogenies (*S*. *flexneri*).

The *S*. *flexneri* maximum likelihood phylogeny (**Fig 2B**, **S2 Fig**) was generated using RAxML v8.2.8 [69], with a GTR+G substitution model and ascertainment bias correction. Dated phylogenies (**Fig 2, S1 & S3 Figs**) were inferred for *S*. *sonnei* and *S*. *dysenteriae*, as these species represent clonal lineages with strong molecular clock signal across their respective species trees. For *S*. *dysenteriae*, we used the dated phylogeny generated previously in Njamkepo *et*. *al* [26]. For *S*. *sonnei*, since six genomes used in the original study were excluded from the present study due to having insufficient read depth for reliable ISMapper analysis, we inferred a new dated phylogeny from the SNV alignment of the 126 included high-depth genomes using BEAST v1.6 [70] and the same model as described in Holt *et al*. Ten chains of 100 million iterations were combined, burn-in removed, and summarised into a MCC tree.

To generate the phylogeny of all *Shigella* reference genomes, *E*. *coli* reference genomes and the non-redundant set of completed *E*. *coli* genomes (**S14 Table**) in **Fig 1A**, we used panaroo v1.2 [71] to generate a core genome alignment of the genomes (using a frequency threshold of 95% to define 2,290 core genes). IQtree v2.0 [72] was used to generate a maximum likelihood phylogeny from this core genome alignment, using a GTR+F+I+G4 substitution model. *E*. *coli* clades were annotated with their Clermont phylogroup using ClermonTyper [73].

To generate phylogenies for each of the five common IS (**S7 Fig**), the *Shigella* and *E*. *coli* reference genomes (listed in **S1 Table**) and the non-redundant set of completed *E*. *coli* genomes (**S14 Table**) were screened for the five common IS using BLAST+ v2.2.30 [60]. BLAST hits with >95% identity and >90% coverage were extracted. Identical nucleotide sequences for each IS were removed, and an alignment was generated with MUSCLE v3.8.1 [74]. ML phylogenies were generated for each alignment using IQTree 1.6.12 [75] using the best-fit substitution model as selected by IQTree.

### Ancestral reconstruction of IS insertion sites in *S*. *sonnei* and *S*. *dysenteriae*

The status (presence/absence) of each IS insertion site at each internal node of the dated phylogenies was inferred using maximum parsimony ancestral state reconstruction, implemented in the *ancestral*.*pars* function in the R package phangorn v2.1.1 [76]. For each IS site, the number of events inferred across the tree (either gain or loss) was then calculated as follows. For each node A where the IS insertion was inferred to be absent, but inferred as present on its parent node B, a loss event was recorded for the branch leading to node A. For each node A where the IS insertion was inferred to be present, but inferred as absent on its parent node B, a gain event was recorded for the branch leading to node A. If there was no change in the inferred IS state between node A and its parent node, then no event was recorded. These results were collated to determine the total number of gain and loss events occurring on each branch (excluding the branches directly descendant from the tree roots as these are non-separable due to lack of outgroups), across all IS insertions.

### Testing for convergent gene loss compared with *S*. *dysenteriae*

Pseudogene content in *S*. *sonnei* or *S*. *flexneri* (all strains, or the subsets of strains from lineages 1 or 3) were compared with that of *S*. *dysenteriae*. We examined whether there were either (a) a significant number of pseudogenes (inactivated in at least one strain of that species) that were fixed pseudogenes in *S*. *dysenteriae* (pseudogenes in ≥90% of genomes); or (b) whether there were a significant number of pseudogenes in each species that were either fixed pseudogenes, or completely missing, in *S*. *dysenteriae*.

First, all genes were extracted from the IS-free reference genomes for each species, and RSD [77] was used to identify homologous genes (see **S9 Table**).

For each target species, we randomly sampled *N* genes (where *N* is the total number of pseudogenes detected in ≥1 strain) and calculated (a) the number of these genes that overlapped with the observed fixed pseudogene complement in *S*. *dysenteriae*, and (b) the number of these genes that overlapped with the observed fixed pseudogene complement in *S*. *dysenteriae* or were absent from the *S*. *dysenteriae* reference genome. We performed this random sampling 1000 times to generate two null distributions for each target species, and compared the observed values to these distribution (**S11 Fig**).

For genes missing in *S*. *dysenteriae* that were present in either *S*. *sonnei* or *S*. *flexneri*, these gene protein sequences were extracted and their frequency in the non-redundant set of *E*. *coli* (from **Fig 1A**) was determined using bi-directional BLAST hits.

### Testing for functional enrichment of interrupted genes

Functional assignments for *Shigella* genes in each reference genome were extracted from RAST [78] annotations of each reference genome, which annotates each CDS using the SEED database. SEED is a curated database of protein families, called FIGfams, which organises genes into functional categories, subcategories and subsystems [79]. Chi-square tests were performed on each SEED category to test whether the proportion of genes within that category were enriched for inactivation across multiple *Shigella* species; p-values were corrected for multiple testing using FDR.

### Assessing correlation between pairwise differences

To assess correlation between pairwise SNV counts vs pairwise distances in terms of IS, pseudogenes or metabolic phenotypes, we conducted Mantel tests using the *mantel*.*test* function in R package ape (v5.3), with 1000 permutations to assess significance.

### Metabolic modelling

Genome-scale models of metabolism were built for each of the *Shigella* species based on the IS-free reference genomes, and for the three EIEC reference genomes based on their full chromosomal sequences. The iML1515 GEM of *E*. *coli* K-12 MG1655 [47] was used as a basis for reconstruction of the *Shigella* and EIEC reference GEMs. Bi-directional best BLAST hits (BBH) were used to detect orthologs between *E*. *coli* K-12 MG1655 and each of the genomes. A BBH with greater than 80% identity was used to assign orthologs between each organism. Genes and their corresponding reactions that were not detected above this threshold were removed from the resulting models. The resulting reference models are available in Figshare (doi:10.6084/m9.figshare.8800673.v1).

Strain-specific *Shigella* GEMs were created by removing genes and their corresponding reactions from the corresponding reference model, using the gene interruption matrices (incorporating IS interruptions and mutational interruptions) for each species.

Each constraints-based model consists of a stoichiometric matrix (**S**) with *m* rows and *n* columns, where *m* is the number of distinct metabolites and *n* is the number of reactions. Each of the *n* reactions has an upper and lower bound on the flux it can carry. Reversible reactions have an upper bound of 1000 mmol gDW^-1^ h^-1^ and a lower bound of -1000 mmol gDW^-1^ h^-1^, while irreversible reactions have a lower bound of zero. Flux Based Analysis (FBA) [46] can be used to identify optimal steady-state flux distributions of constraint-based models. Linear programming is used to find a solution to the equation **Sv** = 0 that optimizes an objective **c**^T^*v, given the set of upper and lower bound constraints. **v** is a vector of reaction fluxes of length *n*. Typically, **c** is a vector of 0s of length *n* with a 1 at the position of the reaction flux to be maximized or minimized. For all growth simulations, the core biomass reaction is set as the objective to be maximized.

### Prediction of different carbon, nitrogen, phosphorus, and sulfur sources

The possible growth-supporting carbon, nitrogen, phosphorus, and sulfur sources for each model were identified using FBA. First, all exchange reactions for extracellular metabolites containing the four elements were identified from the metabolite formulas. Every extracellular compound containing carbon was considered a potential carbon source. Next, to determine possible growth supporting carbon sources, the lower bound of the glucose exchange reaction was constrained to zero. Then the lower bound of each carbon exchange reaction was set, one at a time, to -10 mmol gDW^-1^ h^-1^, and growth was maximized by FBA using the core biomass reaction. The target substrate was considered growth supporting if the predicted growth rate was above zero. While identifying carbon sources, the default nitrogen, phosphorus, and sulfur sources were ammonium (nh4), inorganic phosphate (pi), and inorganic sulfate (so4). Prediction of growth supporting sources for these other three elements was performed in the same manner as growth on carbon, with glucose as the default carbon source.

### Testing for convergent phenotype loss of core *E*. *coli* metabolic phenotypes

Core *E*. *coli* metabolic phenotypes were defined as those predicted in ≥95% of the *E*. *coli* GEMS (excluding *Shigella* and EIEC). To assess significance of overlap between core phenotypes lost from each *Shigella*/EIEC clade with those lost from the most-reduced species *S*. *dysenteriae*, we first calculated the number of core phenotypes lost in each isolate and calculated the median number for each clade, N_clade_ (**Fig 7D**). We then simulated random phenotype loss by sampling without replacement, for each clade, N_clade_ core phenotypes and then calculating the number that overlap with the observed set of core phenotypes lost from *S*. *dysenteriae*. Random sampling was performed 1000 times for each clade, to generate a null distribution against which assess the observed overlapping phenotype loss was compared (**S21 Fig**).

## Supporting information

S1 Fig Heatmap of all IS sites detected in *S*. *dysenteriae* using ISMapper. **a,** Tree is time-calibrated tree as per **Fig 1A**. Columns represent unique IS insertion sites, grouped by IS family and type and coloured by IS as per **Fig 2**. Note that within each IS type, columns are clustered according to the IS insertion site matrix and do not reflect location in the genome. **b,** Tree as in **(a)**, with IS insertion sites shown in order of location along the genome, coloured by IS as per **(a)**.
(PDF)

S2 Fig Heatmap of all IS sites detected in *S*. *flexneri* using ISMapper. **a,** Tree is maximum-likelihood tree as per **Fig 1B**. Columns represent unique IS insertion sites, grouped by IS family and type and coloured by IS as per **Fig 2**. Note that within each IS type, columns are clustered according to the IS insertion site matrix and do not reflect location in the genome. **b,** Tree as in **(a)**, with IS insertion sites shown in order of location along the genome, coloured by IS as per **(a)**.
(PDF)

S3 Fig Heatmap of all IS sites detected in *S*. *sonnei* using ISMapper. Tree is time-calibrated tree as per **Fig 2C.** Columns represent unique IS insertion sites, grouped by IS family and type and coloured by IS as per **Fig 2**. Note that within each IS type, columns are clustered according to the IS insertion site matrix and do not reflect location in the genome. **b,** Tree as in **(a)**, with IS insertion sites shown in order of location along the genome, coloured by IS as per **(a)**.
(PDF)

S4 Fig Permutation test of the linear regression of IS copy number vs year in *S*. *sonnei*. **a,** Slope values for 1000 permutations (grey) with the real slope indicated by the red line. Alpha value for the observed slope is indicated on each plot. **b,** as panel **(a)**, but showing adjusted r squared values for the 1000 permutations. **c,** as panel **(a)**, but showing p values for the 1000 permutations.
(PDF)

S5 Fig Relationship between strain-specific insertions and inferred gains/losses for each IS in each *Shigella* species. Gains and losses summarise the total number of IS insertion/deletion events for each IS type inferred from maximum parsimony ancestral state reconstruction of each IS site (shown in heatmaps in S2–S4 Figs) on each species tree, as described in Methods. As *S*. *flexneri* are lineages are highly divergent, this analysis was conducted separately for the 2 subtrees representing the 2 largest *S*. *flexneri* lineages (1 and 3).
(PDF)

S6 Fig Evolutionary history of IS in *S*. *dysenteriae*. **a,** Scatter plot of IS copy number in each genome (estimated using ISMapper) on year of isolation, points are coloured by lineage. Fitted lines show linear regression of IS copy number against year for each lineage, fitted separately for each lineage. **b,** Phenogram of *S*. *dysenteriae* time-calibrated tree from Fig 1A, mapped to y axis to indicate IS copy number inferred at each node on the tree based on ancestral state reconstruction. Branches are coloured by lineage as per legend.
(PDF)

S7 Fig Maximum-likelihood trees of IS sequences belonging to the five common IS found in *Shigella* and *E*. *coli*. All trees are midpoint rooted. Scale bars show number of substitutions per site. Arrows and labels in panel **a** indicate clade locations of known IS*1* variants listed on ISFinder.
(PDF)

S8 Fig Comparisons of shared and non-shared pseudogenes in each species. **a-b**, Pairwise counts of shared (**a**) or non-shared (**b**) pseudogenes for genomes in each *Shigella* population, divided into comparisons of genomes within the same lineage, or between lineages.
(PDF)

S9 Fig Pseudogene distributions in *Shigella* species. **a,** Number of genes inactivated in at least one genome in each *Shigella* species. Bar segments are coloured by mechanism of inactivation, as per inset legend, and percentages indicate proportion for each bar segment. **b-d,** Number of non-shared pseudogenes in each species, broken down by genetic mechanism of interruption, coloured as per inset legend.
(PDF)

S10 Fig Relationship between pairwise number of non-shared IS, pseudogenes and phenotypes vs pairwise SNV distance, for three *Shigella* species. Scatter plots indicate raw values for all strain pairs, coloured to indicate whether pairs represent within-lineage (blue) or between-lineage comparisons. Linear regression lines and statistics are printed on each plot; slope is calculated from linear regression, p-value from Mantel test comparing the pairwise distance matrices.
(PDF)

S11 Fig Null distribution of pseudogenes in each *Shigella* species as compared to observed fixed or fixed and absent pseudogenes in *S*. *dysenteriae*. **a,** Null distributions (grey) of the number of random genes that overlap with observed fixed pseudogenes in *S*. *dysenteriae*, with the observed overlap shown as a red dot. **b,** Table summarising the observed overlap values, including the percentile and p-value of the real value (red dot in panel **(a)**) as compared to the null distribution. **c,** Null distributions (grey) of the number of random genes that overlap with observed fixed or missing pseudogenes in *S*. *dysenteriae*, with the observed overlap shown as a red dot. **d**, Table summarising the observed overlap values, including the percentile and p-value of the real value (red dot in panel **(a)**) as compared to the null distribution.
(PDF)

S12 Fig Number of inactivated genes by RAST annotation category, for each *Shigella* species. For *S*. *flexneri* and *S*. *sonnei*, bars with darker shading shows the number of genes in that RAST category which have homologs that are also interrupted in *S*. *dysenteriae*.
(PDF)

S13 Fig Strain-specific inferences of metabolic phenotypes (growth on 370 substrates) for each *Shigella*, EIEC and *E*. *coli* genome using GEMS. Phenotypes (columns) and genomes (rows) are ordered via hierarchical clustering of the data matrix, cluster dendrograms are shown. Rows are annotated to indicate which species and lineage each genome belongs to, according to inset legend. Heatmap cells are coloured by the substrate class as per inset legend, with the lightest colour indicating that the phenotype is absent in that genome. Corresponding data is provided in **S10 Table**.
(PDF)

S14 Fig Strain-specific inferences of predicted growth on 213 carbon phenotypes for each *Shigella*, EIEC and *E*. *coli* genome using GEMS. Phenotypes (columns) and genomes (rows) are ordered via hierarchical clustering of the data matrix, cluster dendrograms are shown. Rows are annotated to indicate which species and lineage each genome belongs to, according to inset legend.
(PDF)

S15 Fig Strain-specific inferences of predicted growth on 105 nitrogen substrates for each *Shigella*, EIEC and *E*. *coli* genome using GEMS. Phenotypes (columns) and genomes (rows) are ordered via hierarchical clustering of the data matrix, cluster dendrograms are shown. Rows are annotated to indicate which species and lineage each genome belongs to, according to inset legend.
(PDF)

S16 Fig Strain-specific inferences of predicted growth on 55 phosphorous substrates for each *Shigella*, EIEC and *E*. *coli* genome using GEMS. Phenotypes (columns) and genomes (rows) are ordered via hierarchical clustering of the data matrix, cluster dendrograms are shown. Rows are annotated to indicate which species and lineage each genome belongs to, according to inset legend.
(PDF)

S17 Fig Strain-specific inferences of predicted growth on 13 sulfur substrates for each *Shigella*, EIEC and *E*. *coli* genome using GEMS. Phenotypes (columns) and genomes (rows) are ordered via hierarchical clustering of the data matrix, cluster dendrograms are shown. Rows are annotated to indicate which species and lineage each genome belongs to, according to inset legend.
(PDF)

S18 Fig Metabolic map of alternate carbon sources for *Shigella dysenteriae*. Arrows are coloured by the percentage of genomes within the species that can use that reaction (as per inset legend), based on GEMS predictions for n = 125 strains; grey indicates that the reaction is absent from all genomes. Each reaction is labelled with its name and the percentage of genomes where the reaction is present. Orange circles indicate intermediate metabolites.
(PDF)

S19 Fig Metabolic map of alternate carbon sources for *Shigella flexneri*. Arrows are coloured by the percentage of genomes within the species that can use that reaction (as per inset legend), based on GEMS predictions for n = 343 strains; grey indicates that the reaction is absent from all genomes. Each reaction is labelled with its name and the percentage of genomes where the reaction is present. Orange circles indicate intermediate metabolites.
(PDF)

S20 Fig Metabolic map of alternate carbon sources for *Shigella sonnei*. Arrows are coloured by the percentage of genomes within the species that can use that reaction (as per inset legend), based on GEMS predictions for n = 126 strains; grey indicates that the reaction is absent from all genomes. Each reaction is labelled with its name and the percentage of genomes where the reaction is present. Orange circles indicate intermediate metabolites.
(PDF)

S21 Fig Null distribution of core *E*. *coli* phenotype loss in each *Shigella* species as compared to *S*. *dysenteriae*. **a,** Null distributions (grey) of the number of random phenotypes that overlap with core *E*. *coli* phenotypes, with observed overlap shown as a red dot. **b,** Table summarising the observed overlap values, including the percentile and p-value of the real value (red dot in panel **(a)**) as compared to the null distribution.
(PDF)

S22 Fig Breakdown of initial inactivation events for all metabolic reactions in *Shigella* GEMs. **a/c/e,** Total number of initial inactivation events by inactivation type (IS, mutation or tied for either). Each type has been broken down by the hypothesised age of the event–ancient events are conserved in ≥ 80% of genomes, intermediate events are present in >20% and <80% of genomes, recent events occur in ≤ 20% of genomes. **b/d/f,** Percentage of genomes carrying each initial inactivation event, broken down by mechanism of inactivation (as per legend) in each *Shigella* species.
(PDF)

S1 Text Ancestral state reconstruction in *S*. *sonnei*. (DOCX)

S1 Table Features of selected reference genomes. (XLSX)

S2 Table Features of virulence plasmids found in *Shigella* reference genomes, and other plasmids found in the *E*. *coli* reference genomes. No plasmid sequence data was found for *E*. *coli* O157:H7 str. EDL993.
(XLSX)

S3 Table ISMapper detected positions in all *S*. *sonnei* genomes, using the IS-free version of 53G as a reference. Zero indicates position absent, the numeral one indicates position present. X and y coordinates give genome location of hit, relative to the IS-free 53G reference genome.
(XLSX)

S4 Table ISMapper detected positions in all *S*. *dysenteriae* genomes, using the IS-free version of Sd197 as a reference. Zero indicates position absent, the numeral one indicates position present. X and y coordinates give genome location of hit, relative to the IS-free Sd197 reference genome.
(XLSX)

S5 Table ISMapper detected positions in all *S*. *flexneri* genomes, using the IS-free version of 301 as a reference. Zero indicates position absent, the numeral one indicates position present. X and y coordinates give genome location of hit, relative to the IS-free 301 reference genome.
(XLSX)

S6 Table Counts and proportions of IS insertion sites inferred by ancestral state reconstruction to be present in the MRCAs of *S*. *sonnei* lineages. (XLSX)

S7 Table IS counts for the five common *Shigella* IS identified in the non-redundant set of completed *E*. *coli* chromosomes. (XLSX)

S8 Table Accessions for read sets from the six pathogenic *E*. *coli* lineages analysed in Fig 5. (XLSX)

S9 Table Details of genes annotated in the three main *Shigella* reference genomes. IDs for homologs in each reference genome are provided, ‘-‘ indicates no homolog is present. Prevalence columns indicate the proportion of genomes in each species population datasets that lack an intact copy of the gene. Functional annotations from RAST, based on the SEED database, are also provided.
(XLSX)

S10 Table Details of reaction model for each *Shigella* reference genome. (CSV)

S11 Table Presence (1) or absence (0) of each reaction within each *Shigella* reference genome and the *E*. *coli* reference GEM. (CSV)

S12 Table Summary of growth phenotypes in (a) each reference GEM and (b) across all strain-specific GEMs stratified by *Shigella* species and the six pathogenic *E*. *coli* lineages. Phenotype categories referred to in the text (core, accessory, rare) are also annotated.
(XLSX)

S13 Table Presence or absence of each growth phenotype within each *Shigella*, EIEC and *E*. *coli* genome, inferred from GEMS. Column 2 indicates the species and lineage (for *Shigella*) or pathotype (for *E*. *coli*).
(XLSX)

S14 Table Details of non-redundant *E*. *coli* and non-reference *Shigella* genomes included in phylogeny in Fig 1A. (XLSX)
