# Supplementary figures and images for: Impact of insertion sequences on convergent evolution of Shigella species
Source: PLoS Genet. 2020 Jul 9;16(7):e1008931. doi: 10.1371/journal.pgen.1008931 (PMC7373316; doi:10.1371/journal.pgen.1008931)

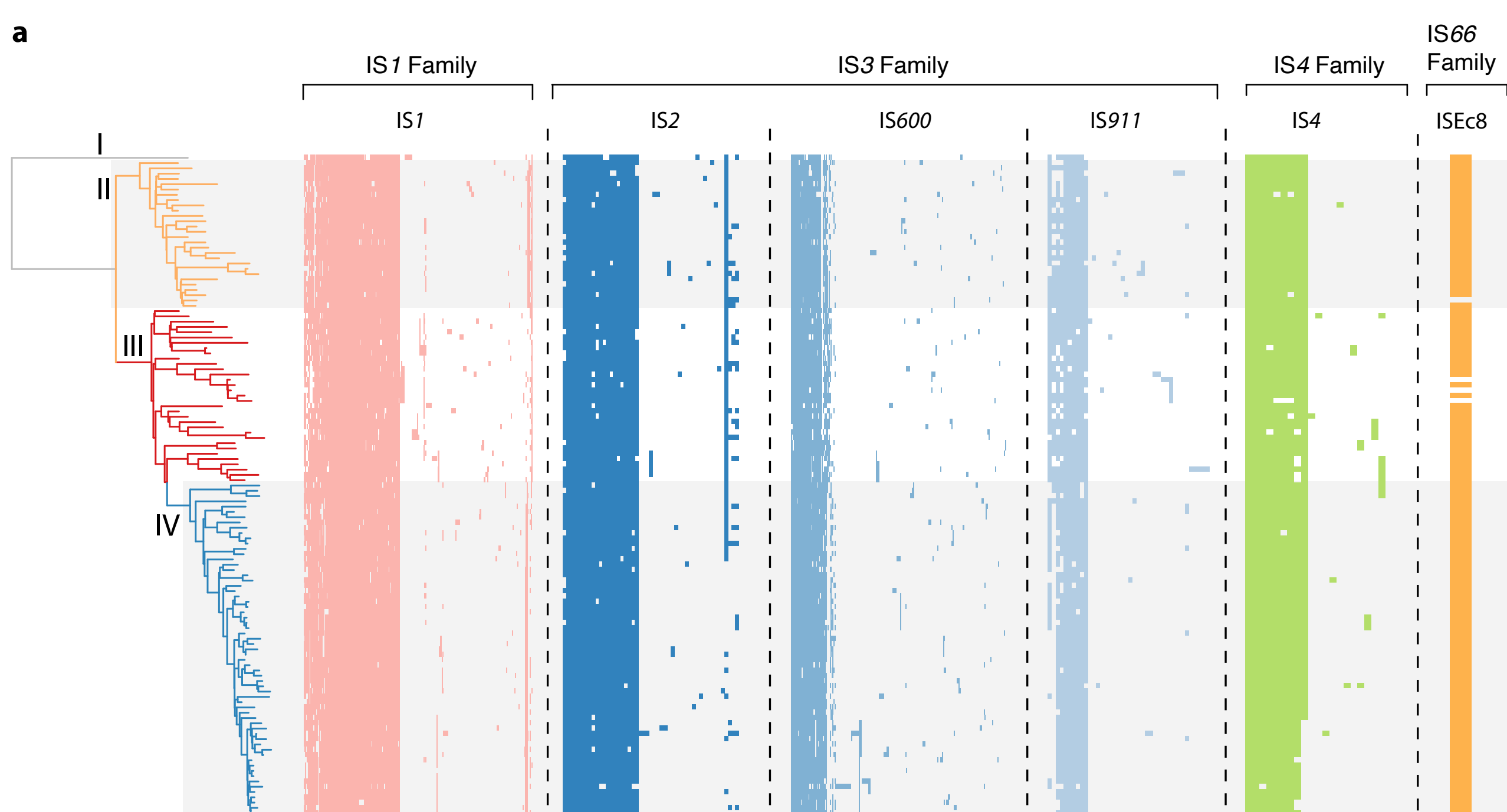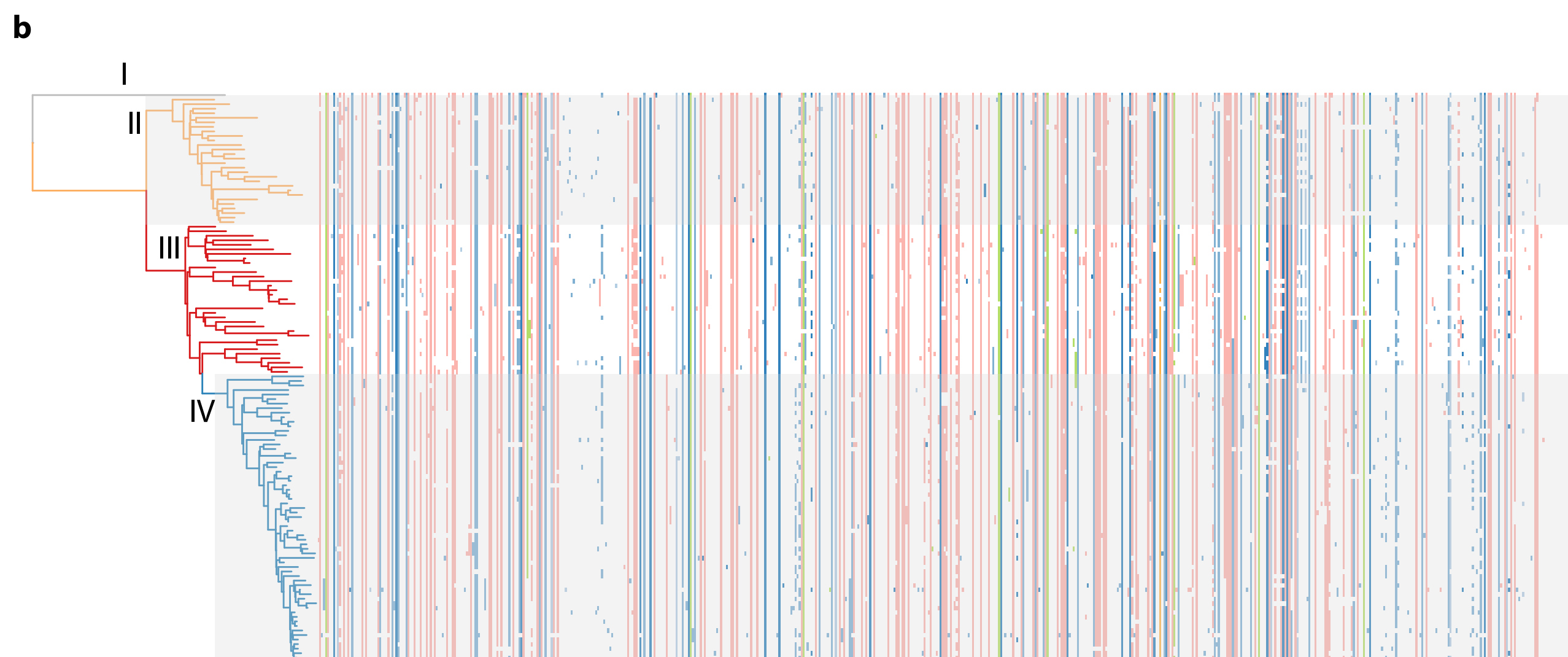

Supplement: S1 Fig — a, Tree is time-calibrated tree as per Fig 1A. Columns represent unique IS insertion sites, grouped by IS family and type and coloured by IS as per Fig 2. Note that within each IS type, columns are clustered according to the IS insertion site matrix and do not reflect location in the genome. b, Tree as in (a), with IS insertion sites shown in order of location along the genome, coloured by IS as per (a). (PDF) [file pgen.1008931.s001.pdf]

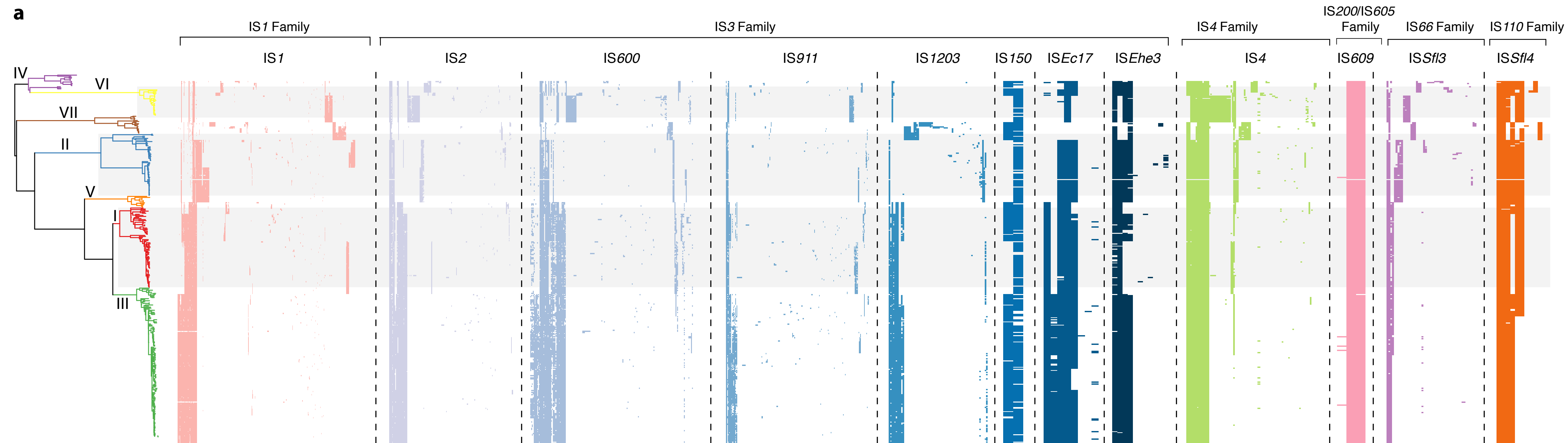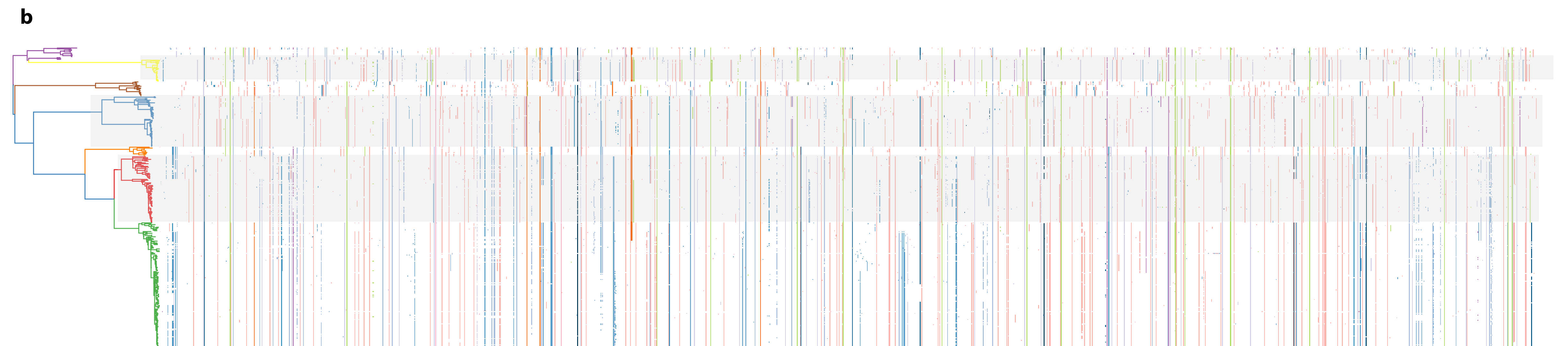

Supplement: S2 Fig — a, Tree is maximum-likelihood tree as per Fig 1B. Columns represent unique IS insertion sites, grouped by IS family and type and coloured by IS as per Fig 2. Note that within each IS type, columns are clustered according to the IS insertion site matrix and do not reflect location in the genome. b, Tree as in (a), with IS insertion sites shown in order of location along the genome, coloured by IS as per (a). (PDF) [file pgen.1008931.s002.pdf]

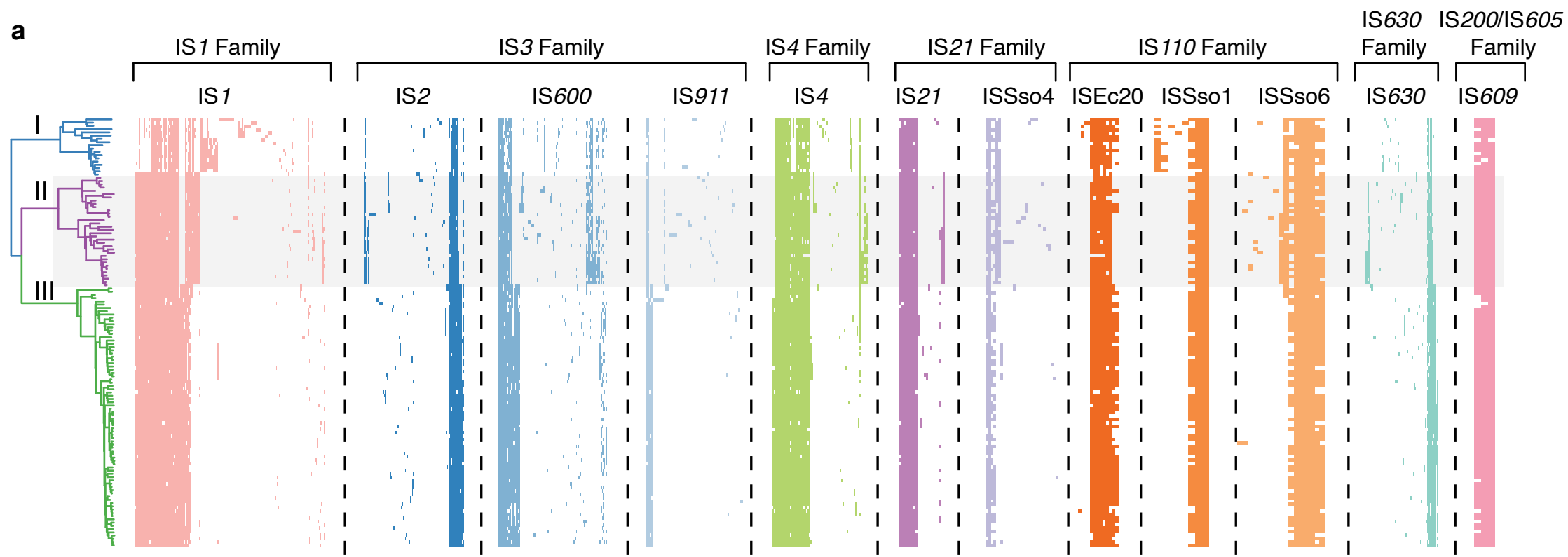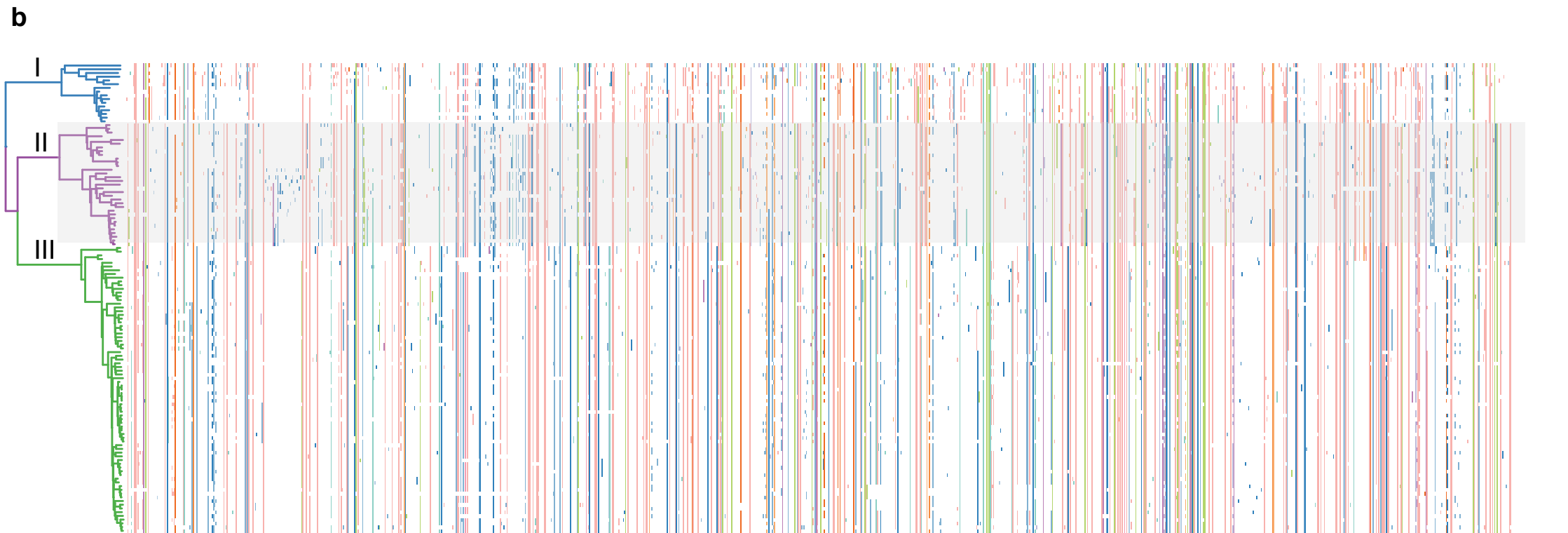

Supplement: S3 Fig — Tree is time-calibrated tree as per Fig 2C. Columns represent unique IS insertion sites, grouped by IS family and type and coloured by IS as per Fig 2. Note that within each IS type, columns are clustered according to the IS insertion site matrix and do not reflect location in the genome. b, Tree as in (a), with IS insertion sites shown in order of location along the genome, coloured by IS as per (a). (PDF) [file pgen.1008931.s003.pdf]

**a**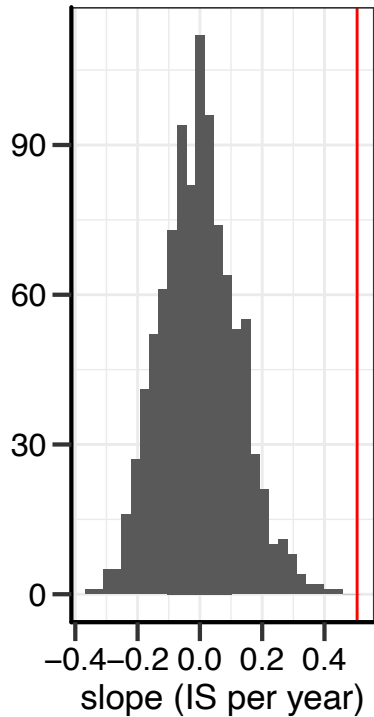**b**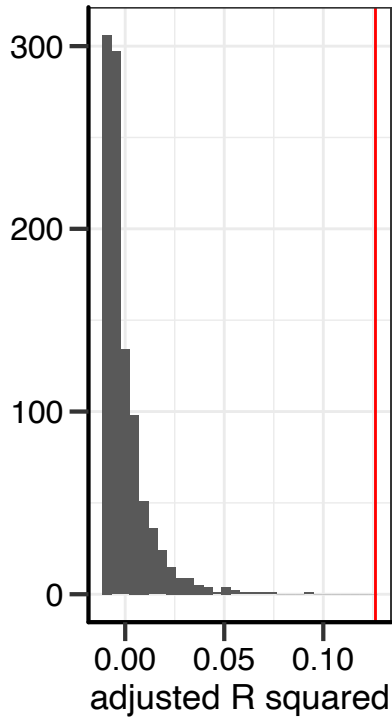**c**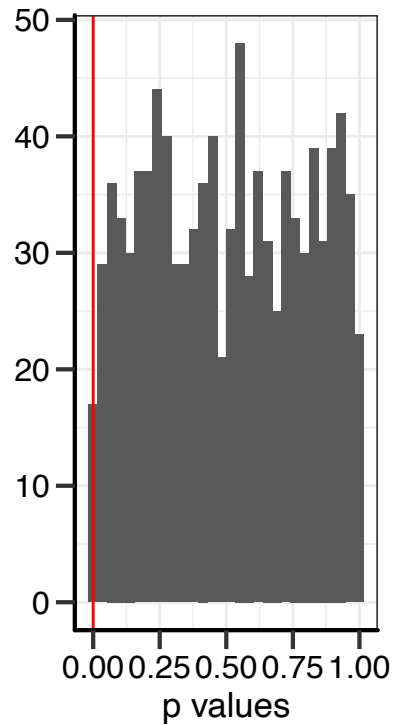

Supplement: S4 Fig — a, Slope values for 1000 permutations (grey) with the real slope indicated by the red line. Alpha value for the observed slope is indicated on each plot. b, as panel (a), but showing adjusted r squared values for the 1000 permutations. c, as panel (a), but showing p values for the 1000 permutations. (PDF) [file pgen.1008931.s004.pdf]

*S. sonnei*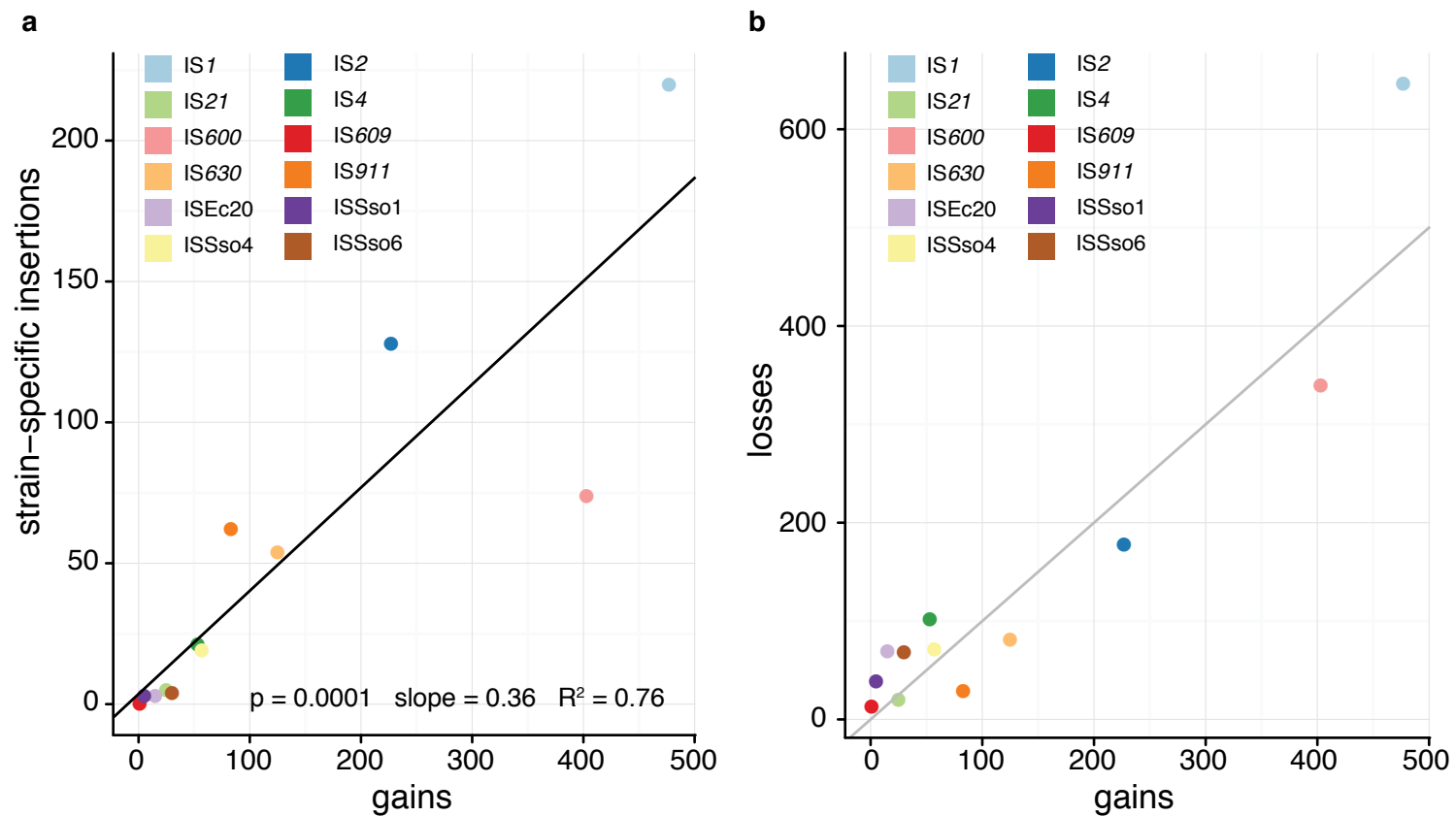*S. flexneri* lineage 1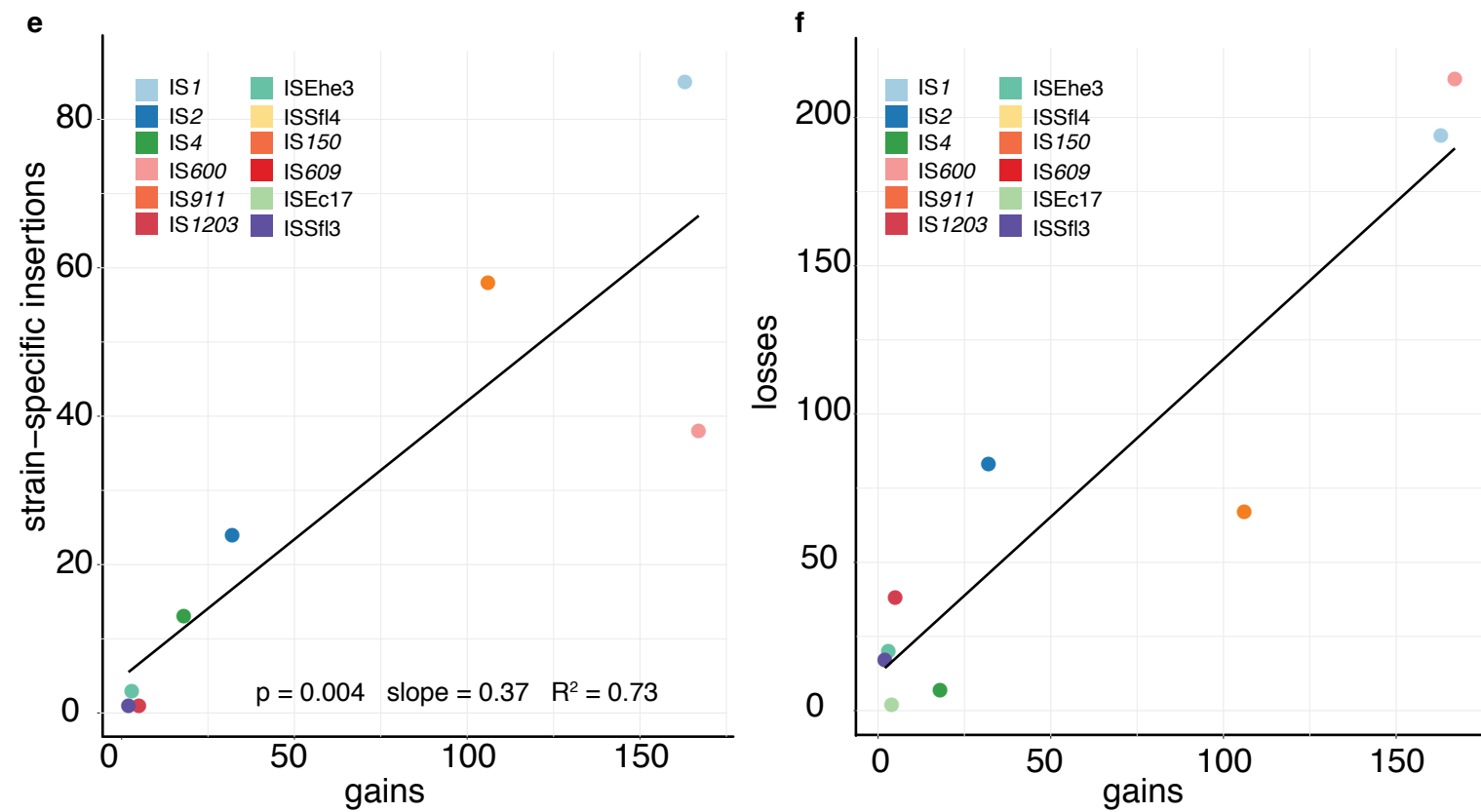*S. dysenteriae*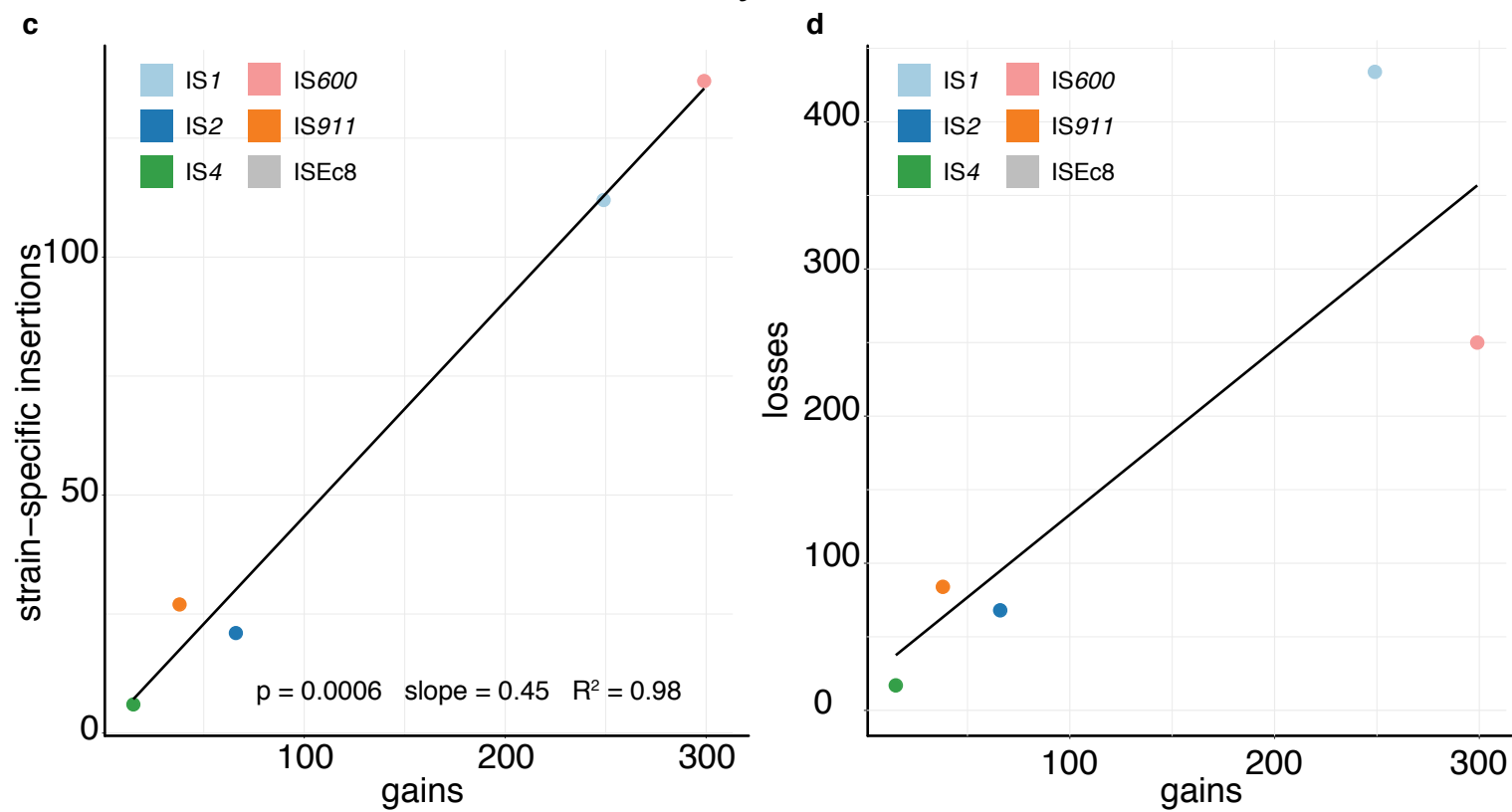*S. flexneri* lineage 3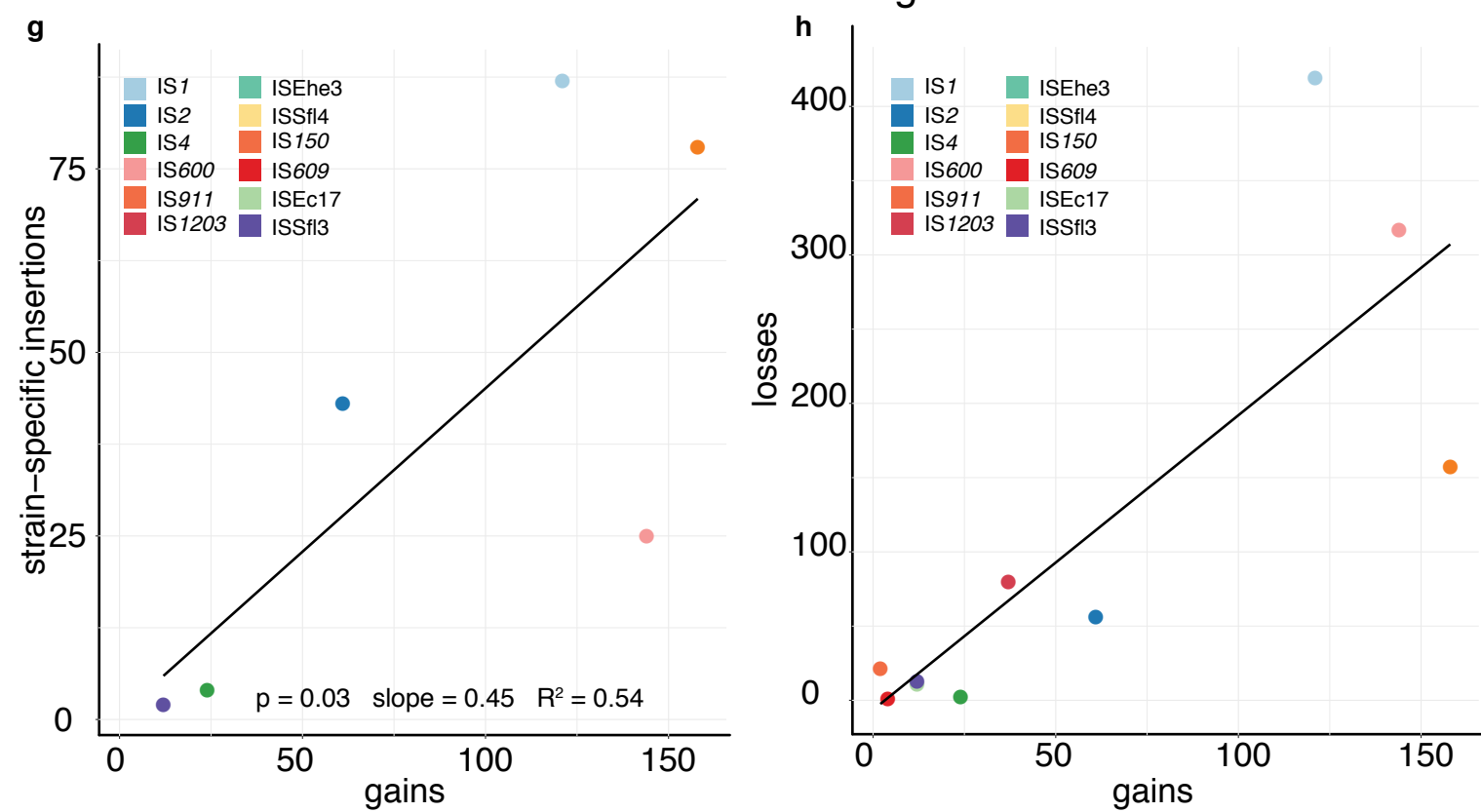

Supplement: S5 Fig — Gains and losses summarise the total number of IS insertion/deletion events for each IS type inferred from maximum parsimony ancestral state reconstruction of each IS site (shown in heatmaps in S2–S4 Figs) on each species tree, as described in Methods. As S. flexneri are lineages are highly divergent, this analysis was conducted separately for the 2 subtrees representing the 2 largest S. flexneri lineages (1 and 3). (PDF) [file pgen.1008931.s005.pdf]

a

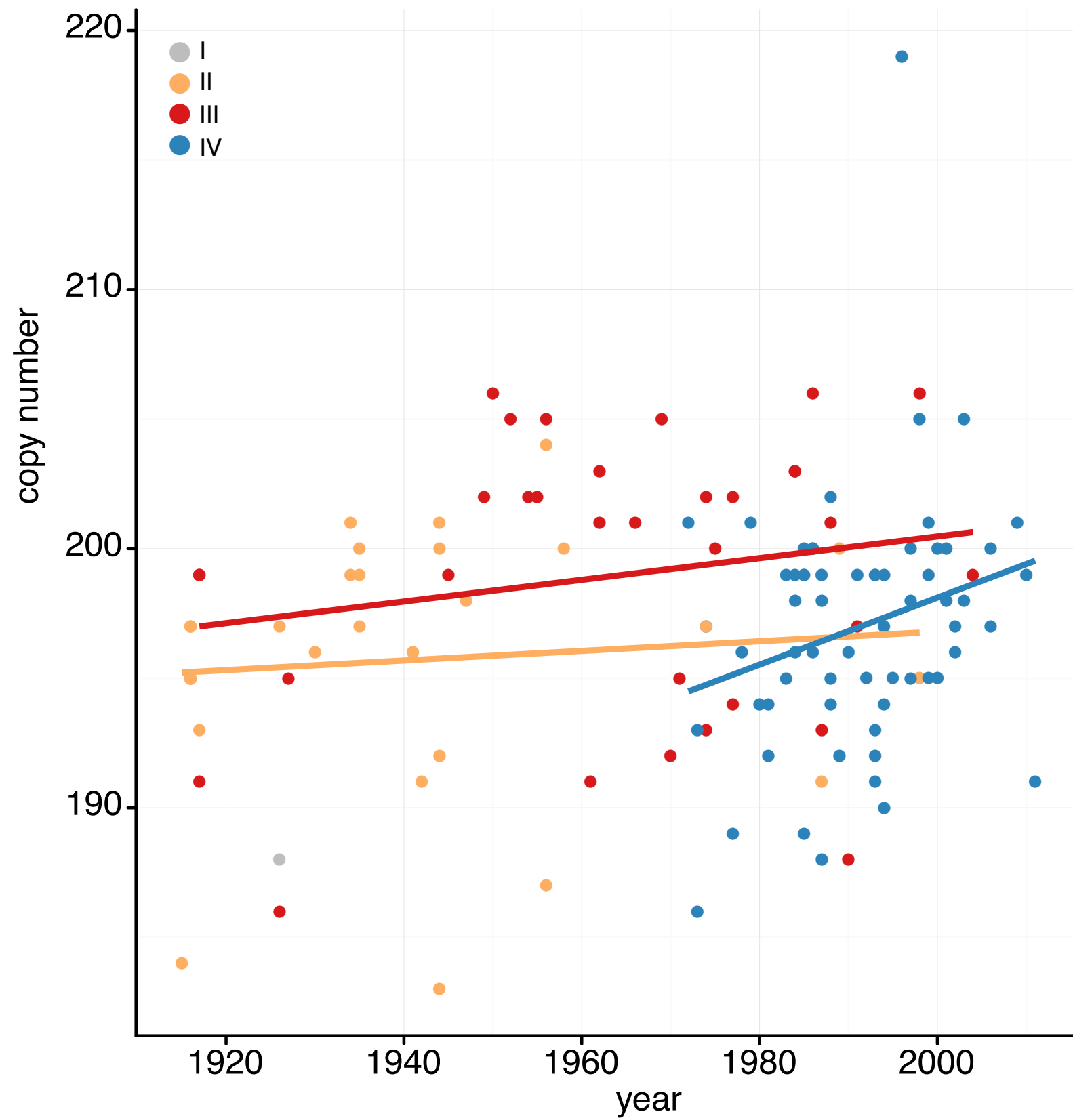

b

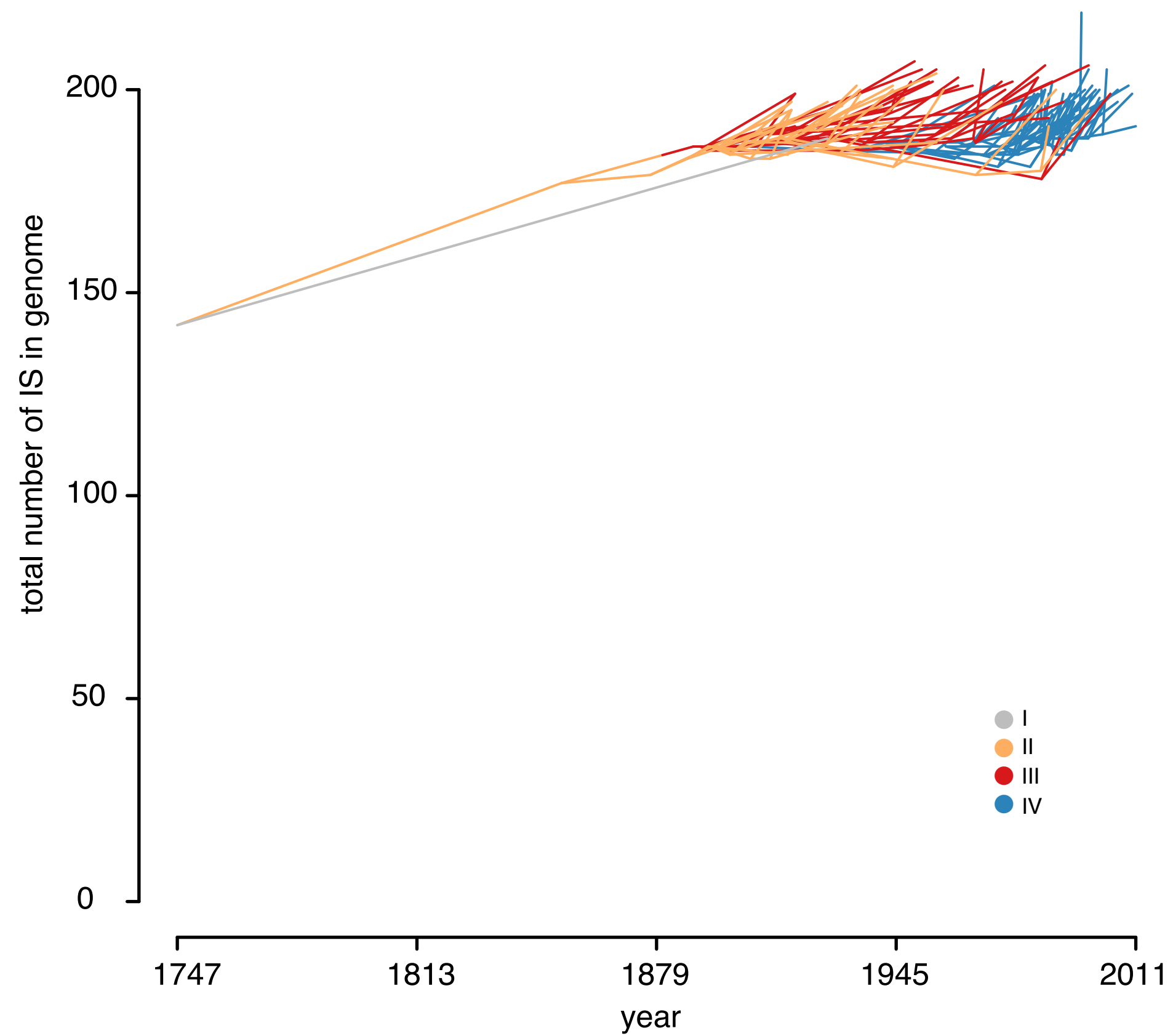

Supplement: S6 Fig — a, Scatter plot of IS copy number in each genome (estimated using ISMapper) on year of isolation, points are coloured by lineage. Fitted lines show linear regression of IS copy number against year for each lineage, fitted separately for each lineage. b, Phenogram of S. dysenteriae time-calibrated tree from Fig 1A, mapped to y axis to indicate IS copy number inferred at each node on the tree based on ancestral state reconstruction. Branches are coloured by lineage as per legend. (PDF) [file pgen.1008931.s006.pdf]

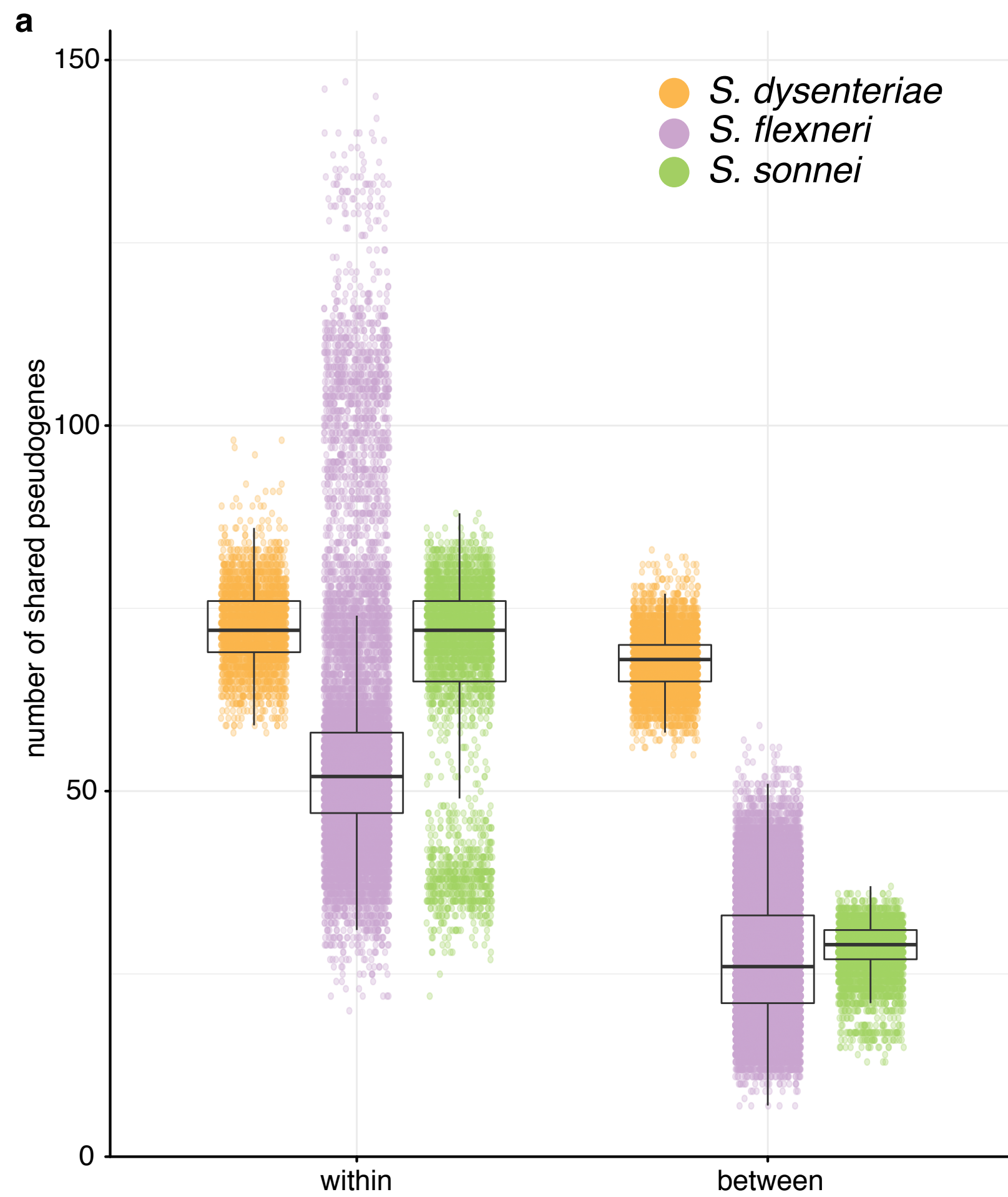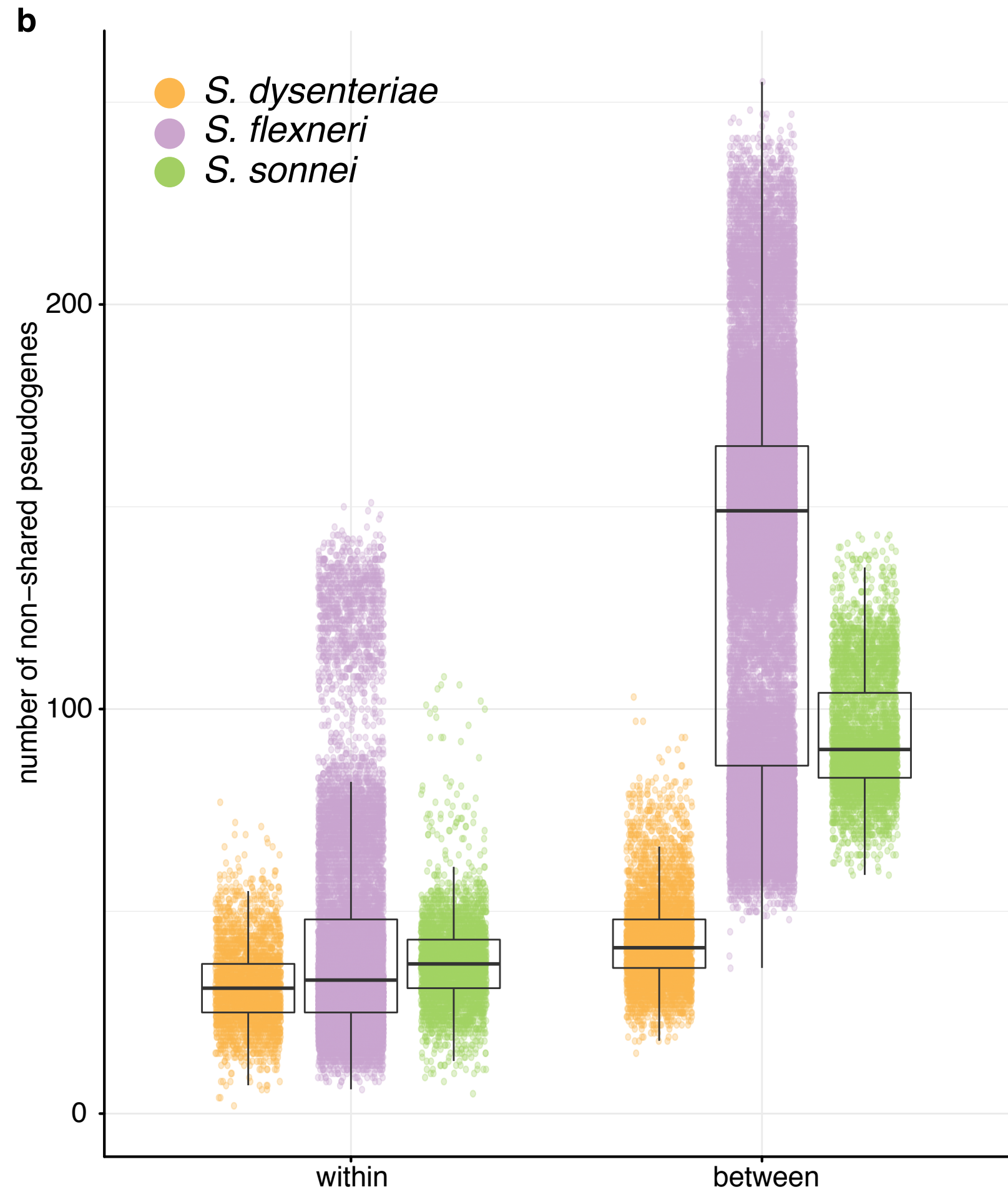

Supplement: S8 Fig — a-b, Pairwise counts of shared (a) or non-shared (b) pseudogenes for genomes in each Shigella population, divided into comparisons of genomes within the same lineage, or between lineages. (PDF) [file pgen.1008931.s008.pdf]

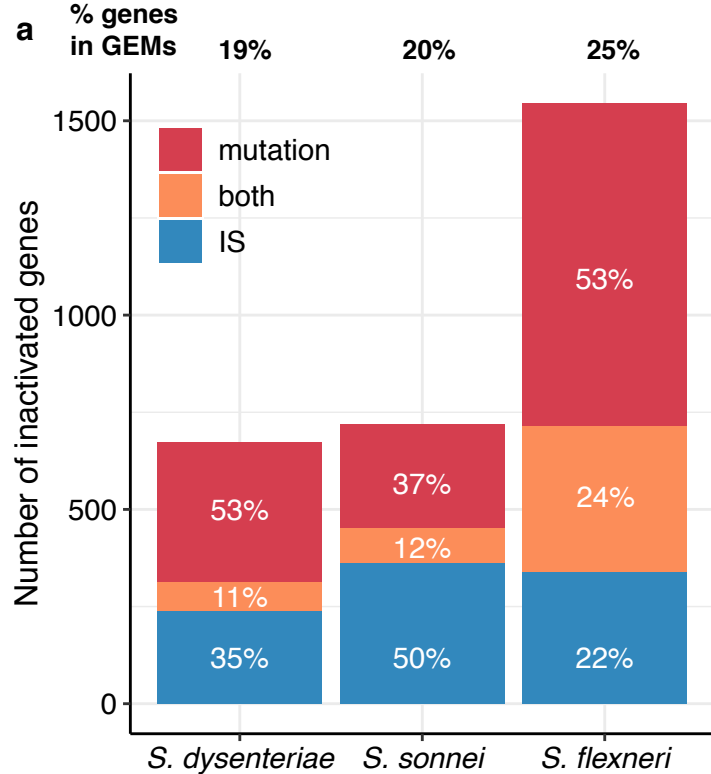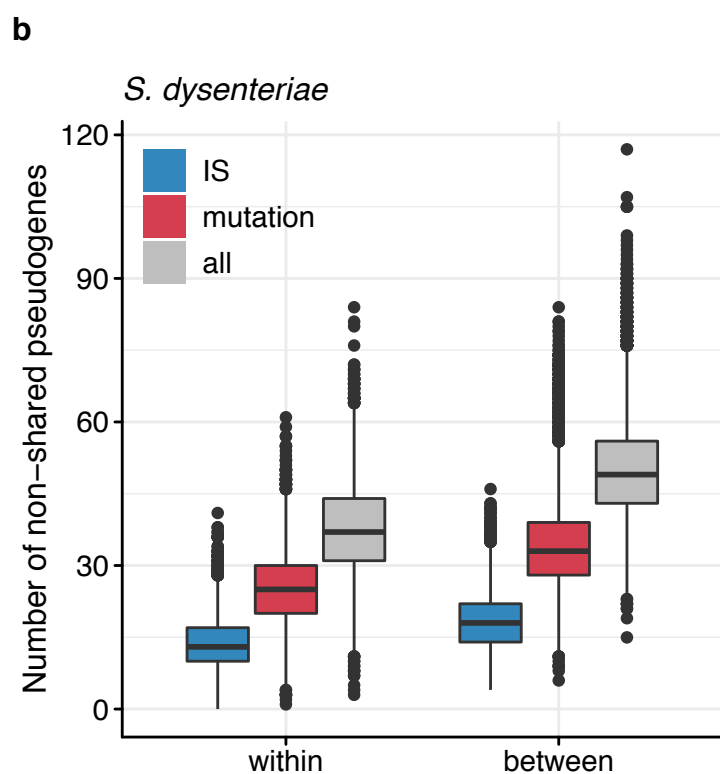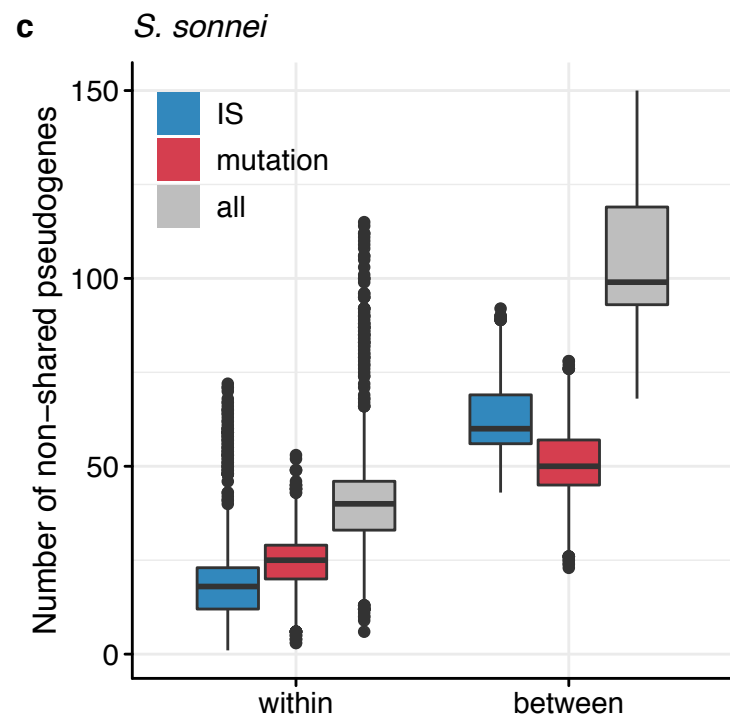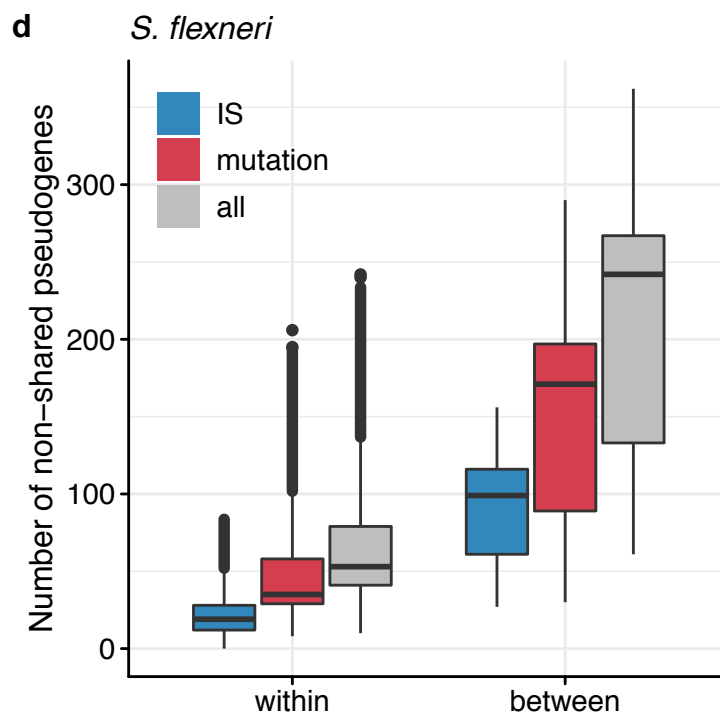

Supplement: S9 Fig — a, Number of genes inactivated in at least one genome in each Shigella species. Bar segments are coloured by mechanism of inactivation, as per inset legend, and percentages indicate proportion for each bar segment. b-d, Number of non-shared pseudogenes in each species, broken down by genetic mechanism of interruption, coloured as per inset legend. (PDF) [file pgen.1008931.s009.pdf]

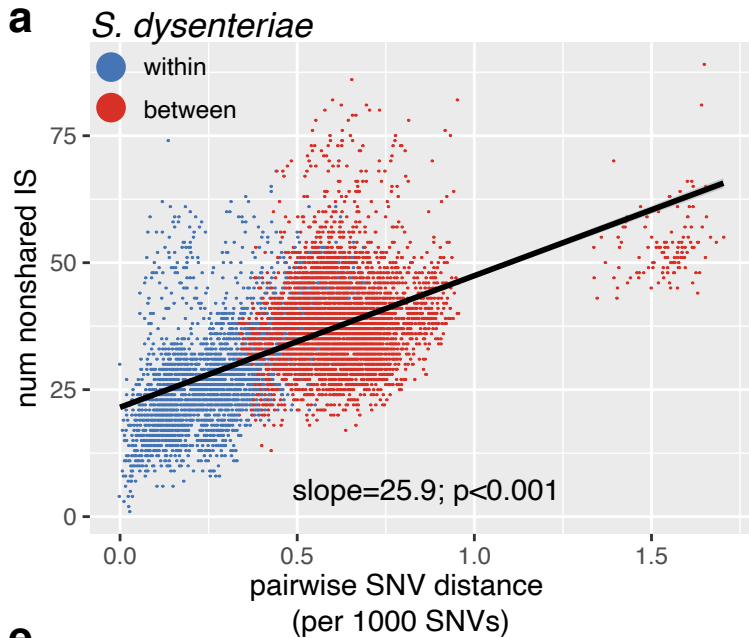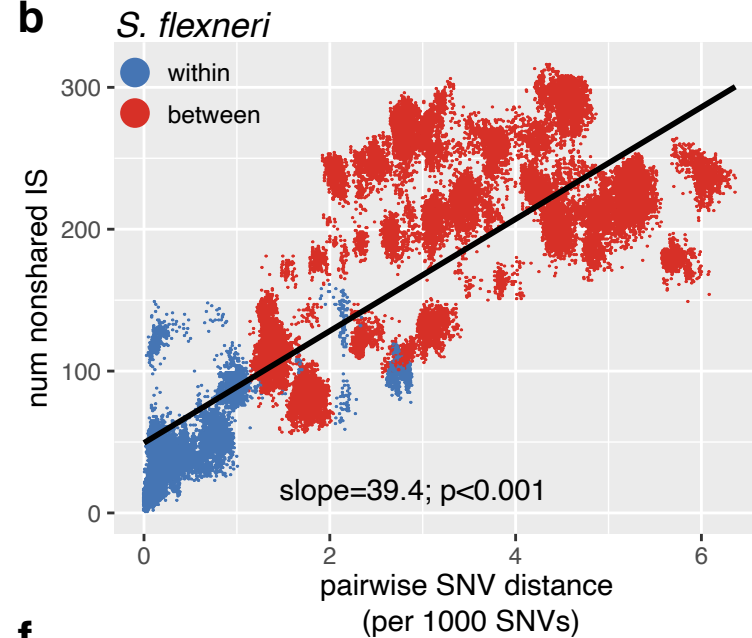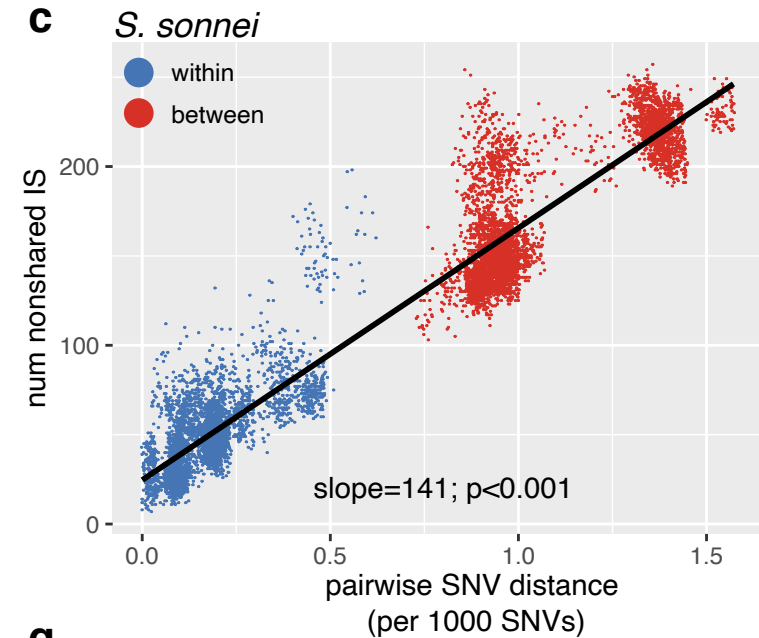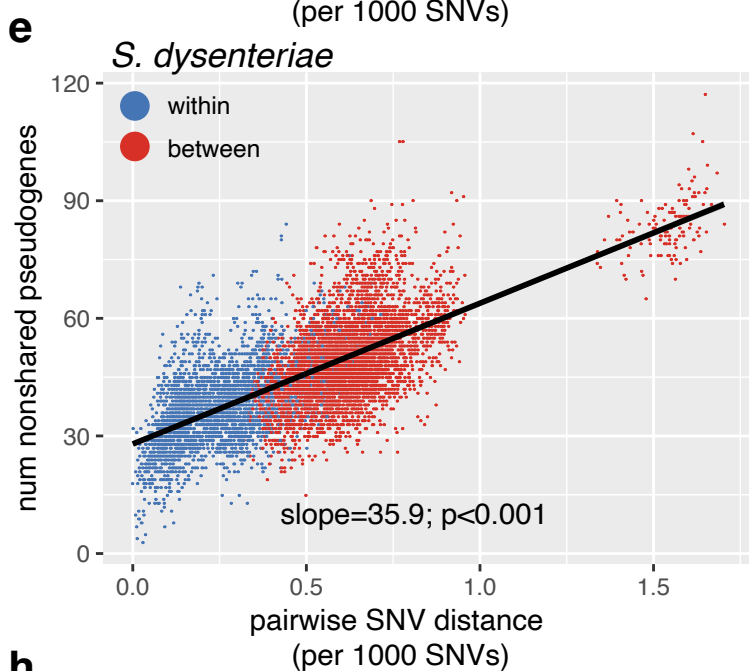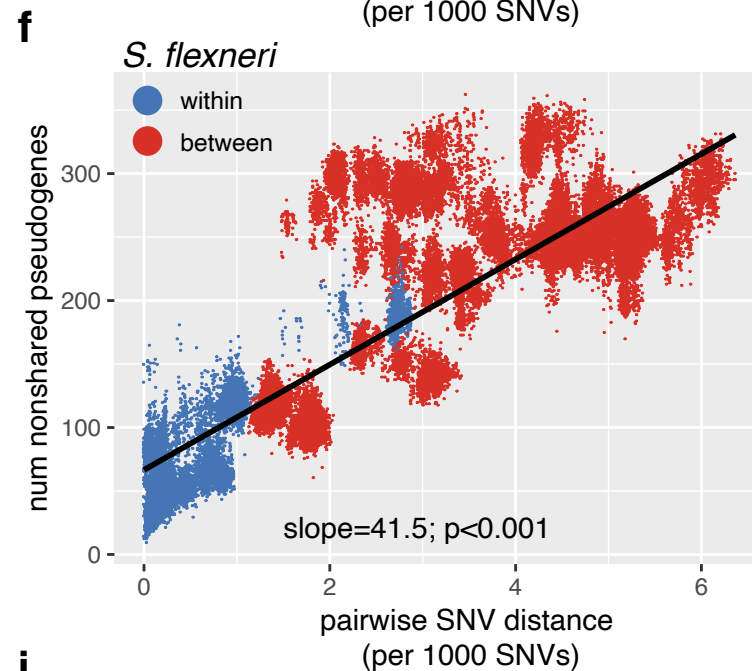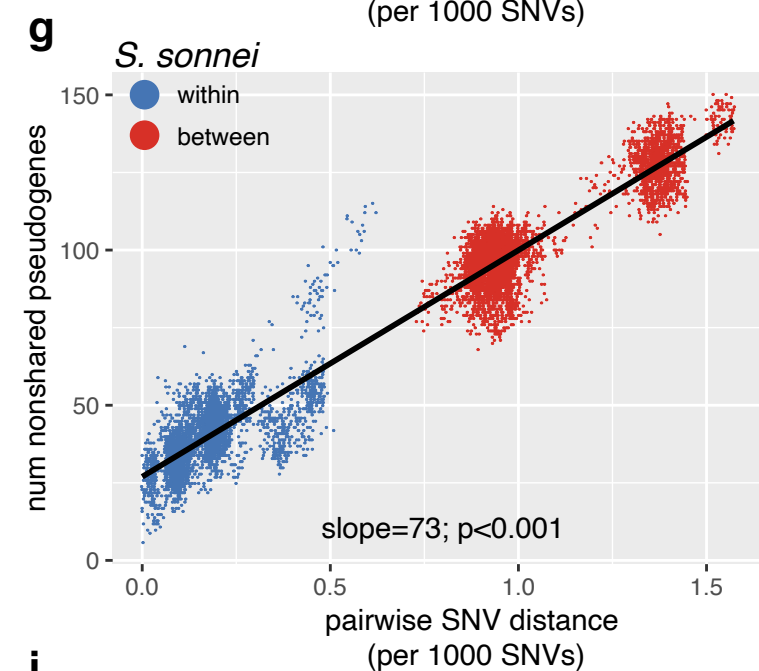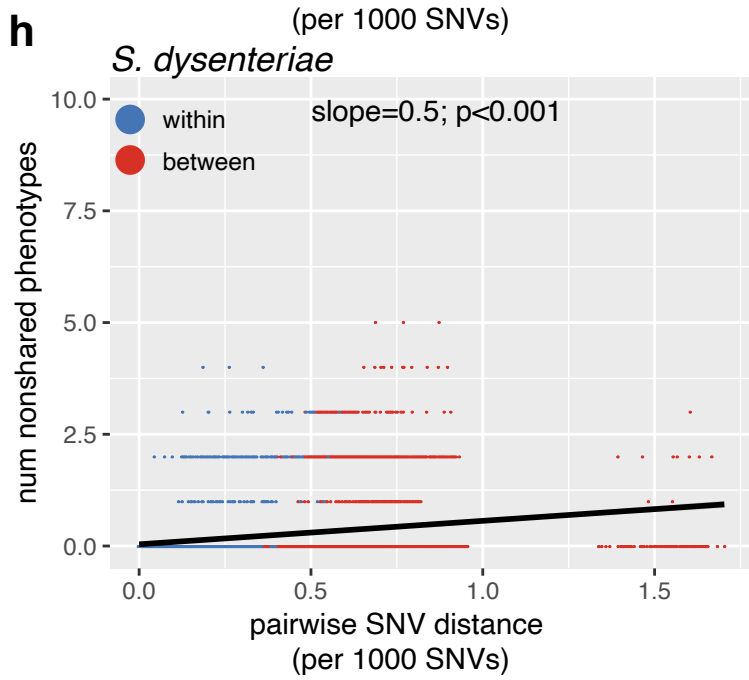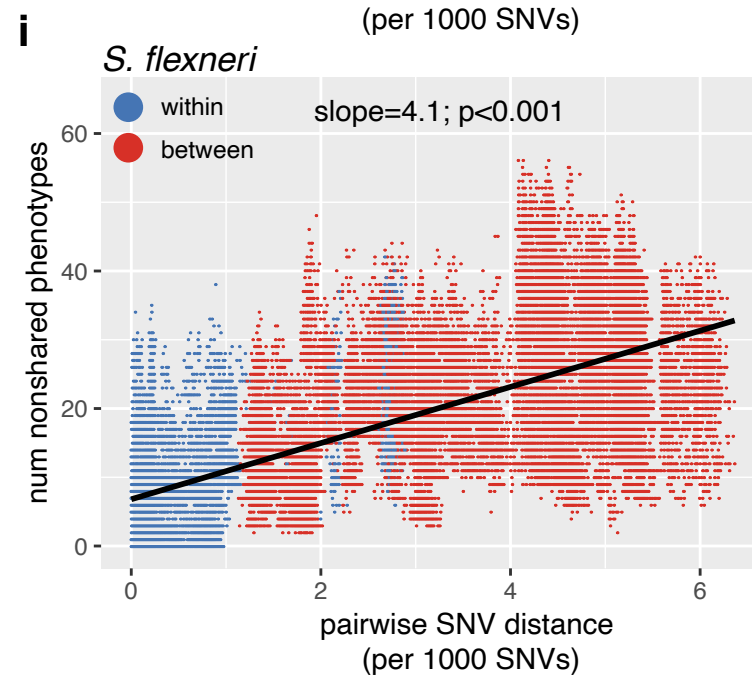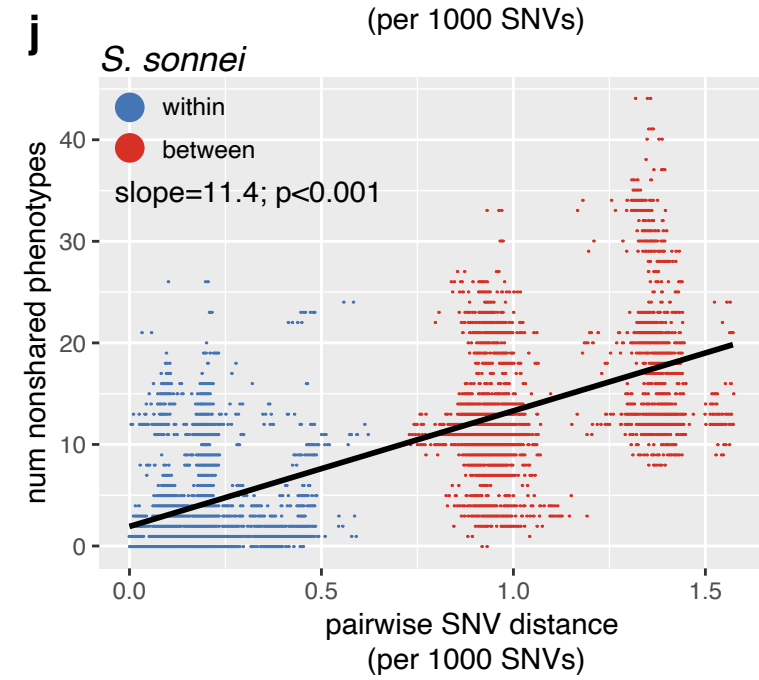

Supplement: S10 Fig — Scatter plots indicate raw values for all strain pairs, coloured to indicate whether pairs represent within-lineage (blue) or between-lineage comparisons. Linear regression lines and statistics are printed on each plot; slope is calculated from linear regression, p-value from Mantel test comparing the pairwise distance matrices. (PDF) [file pgen.1008931.s010.pdf]

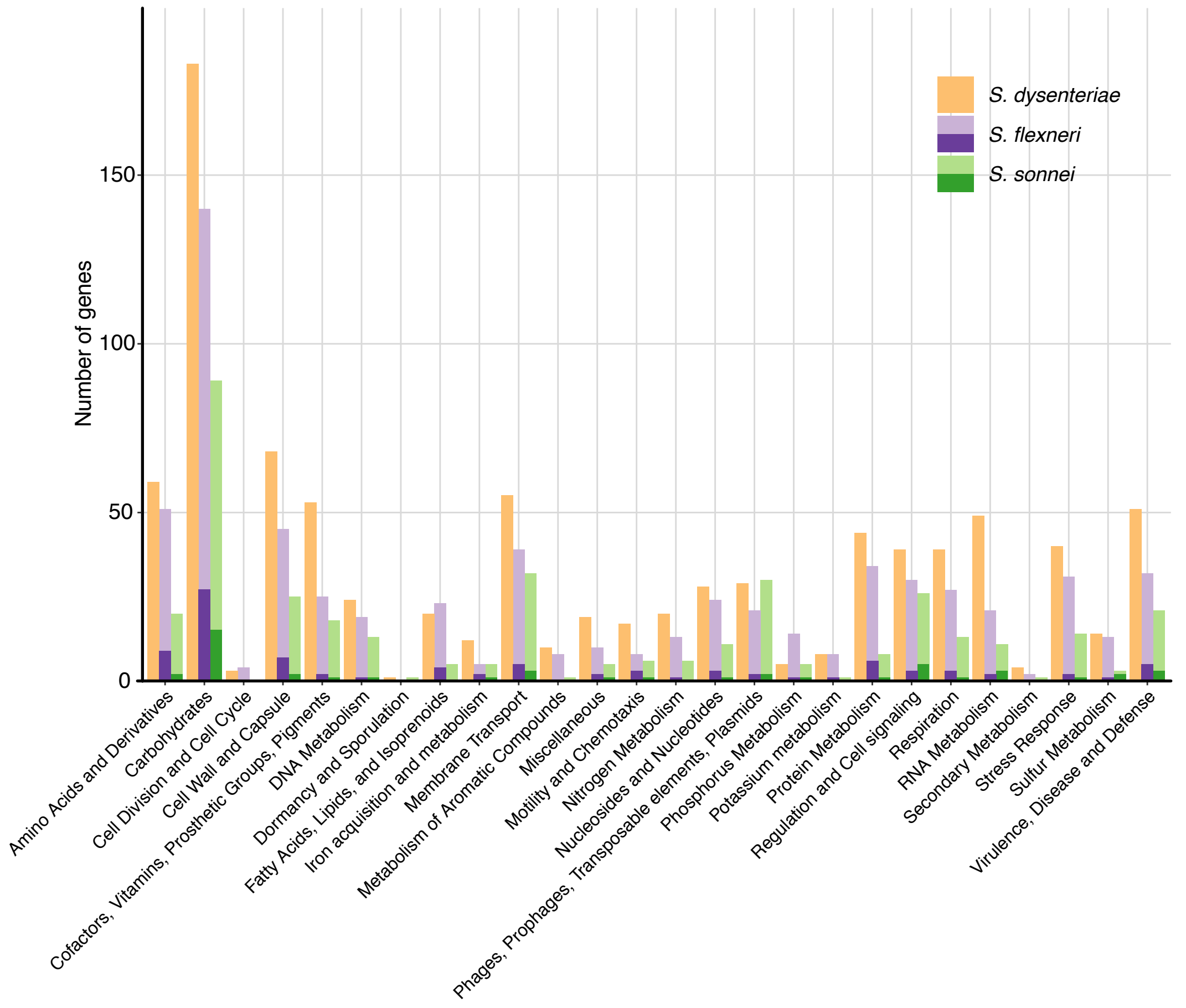

Supplement: S12 Fig — For S. flexneri and S. sonnei, bars with darker shading shows the number of genes in that RAST category which have homologs that are also interrupted in S. dysenteriae. (PDF) [file pgen.1008931.s012.pdf]

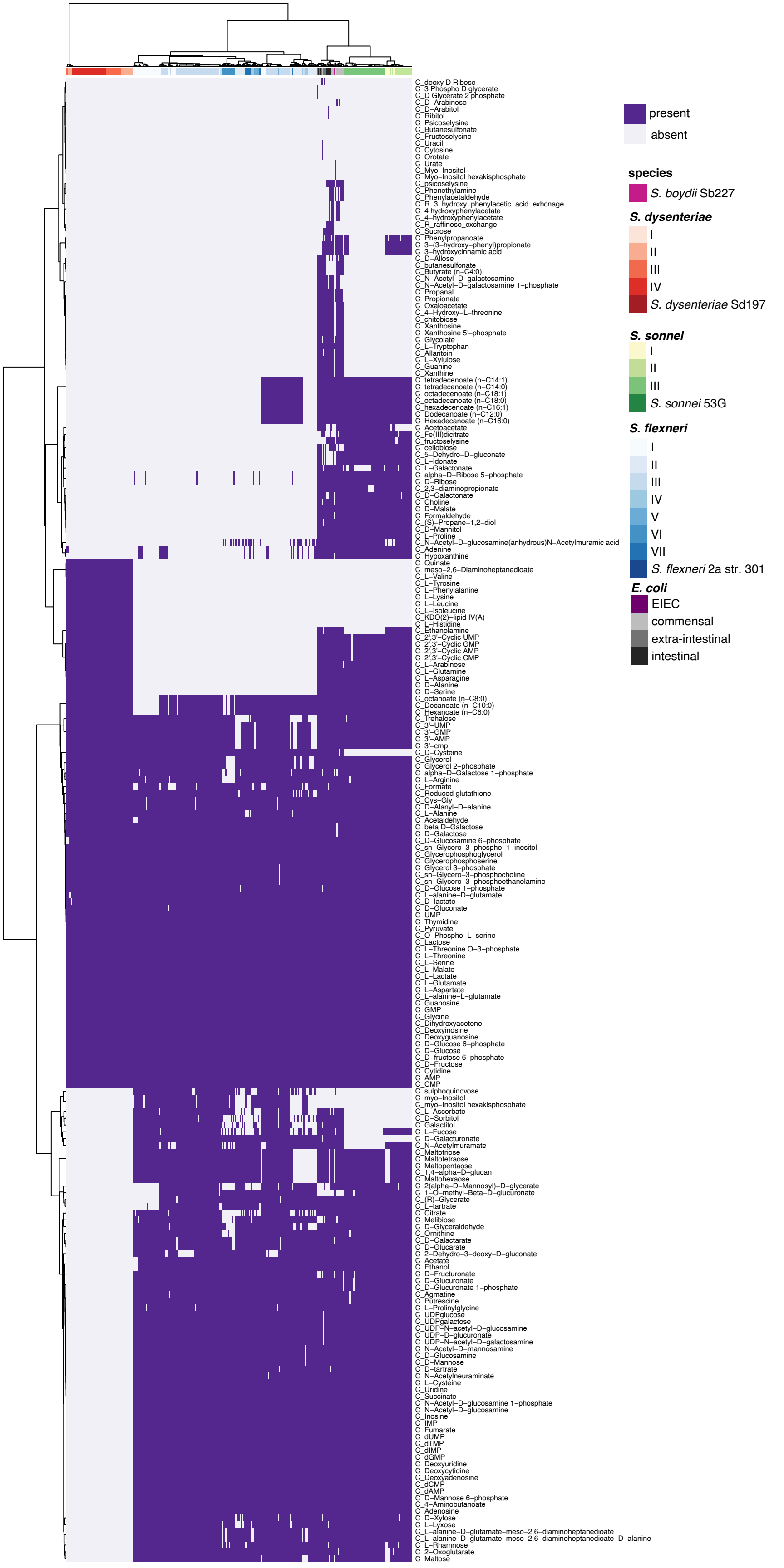

Supplement: S14 Fig — Phenotypes (columns) and genomes (rows) are ordered via hierarchical clustering of the data matrix, cluster dendrograms are shown. Rows are annotated to indicate which species and lineage each genome belongs to, according to inset legend. (PDF) [file pgen.1008931.s014.pdf]

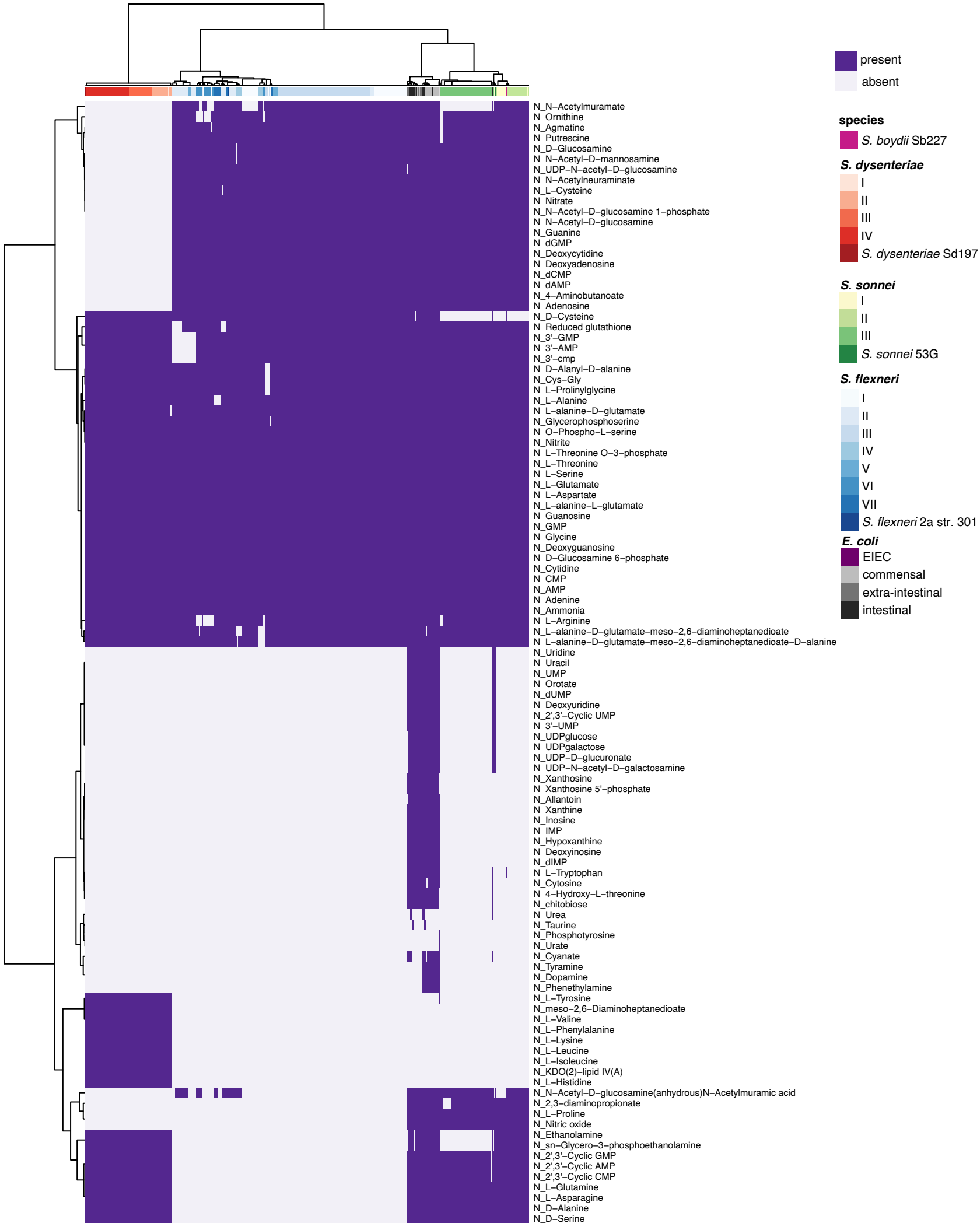

Supplement: S15 Fig — Phenotypes (columns) and genomes (rows) are ordered via hierarchical clustering of the data matrix, cluster dendrograms are shown. Rows are annotated to indicate which species and lineage each genome belongs to, according to inset legend. (PDF) [file pgen.1008931.s015.pdf]

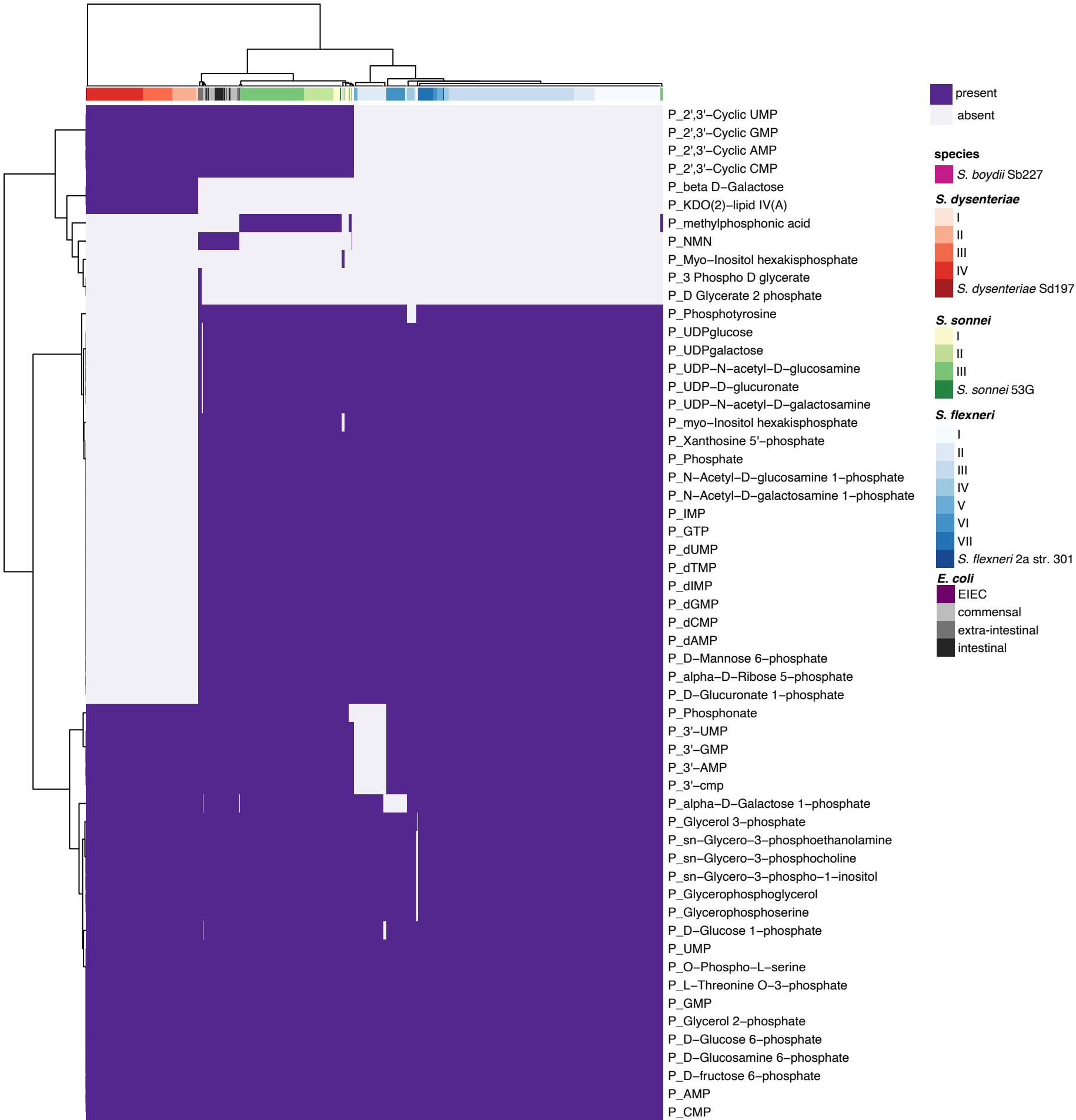

Supplement: S16 Fig — Phenotypes (columns) and genomes (rows) are ordered via hierarchical clustering of the data matrix, cluster dendrograms are shown. Rows are annotated to indicate which species and lineage each genome belongs to, according to inset legend. (PDF) [file pgen.1008931.s016.pdf]

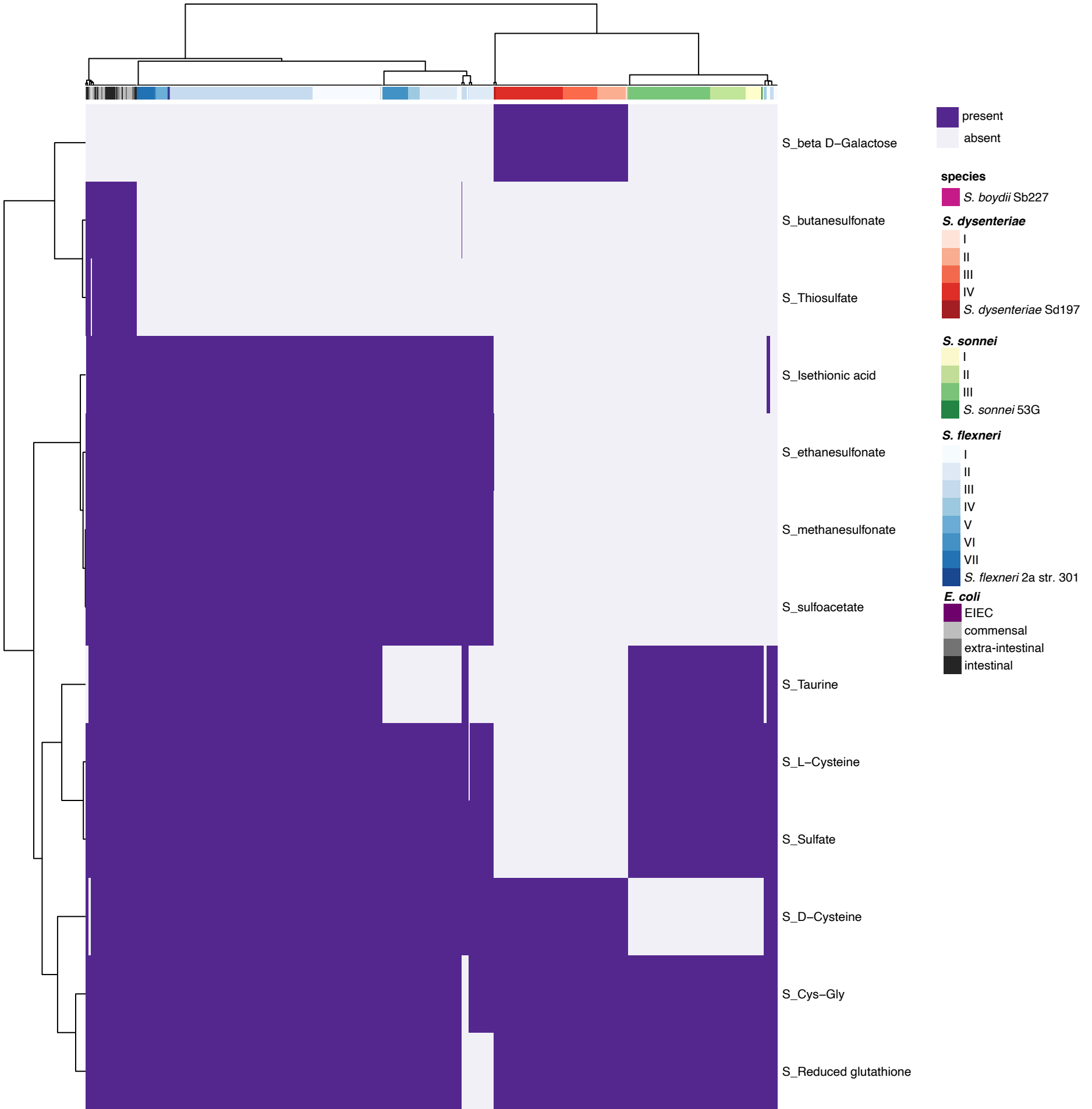

Supplement: S17 Fig — Phenotypes (columns) and genomes (rows) are ordered via hierarchical clustering of the data matrix, cluster dendrograms are shown. Rows are annotated to indicate which species and lineage each genome belongs to, according to inset legend. (PDF) [file pgen.1008931.s017.pdf]

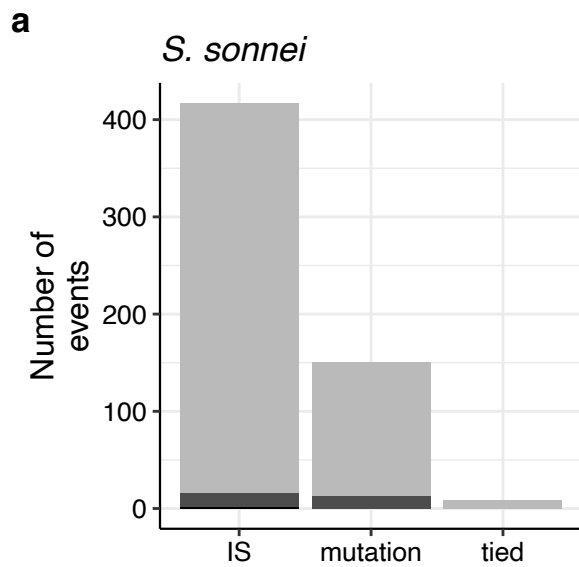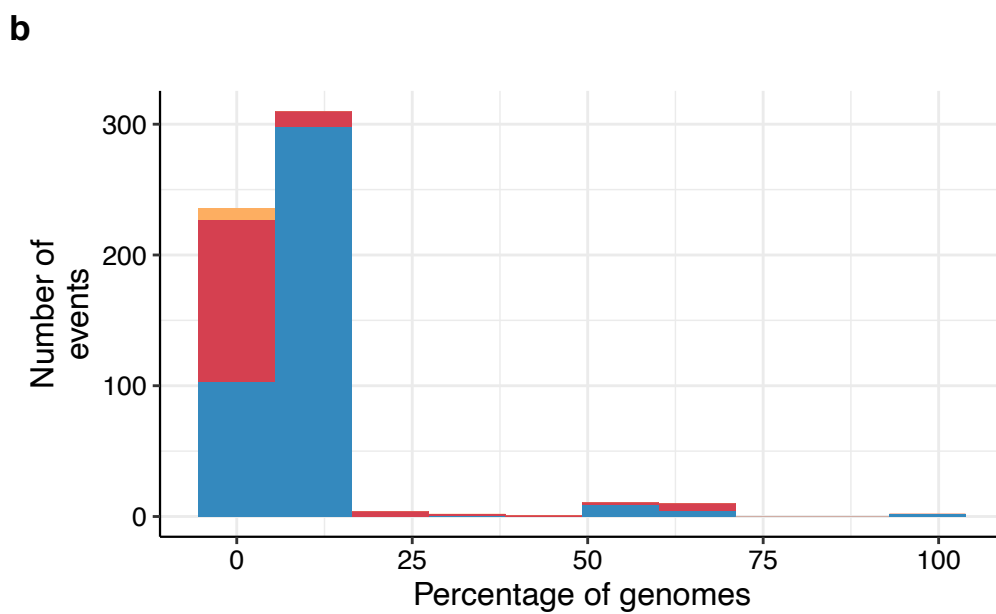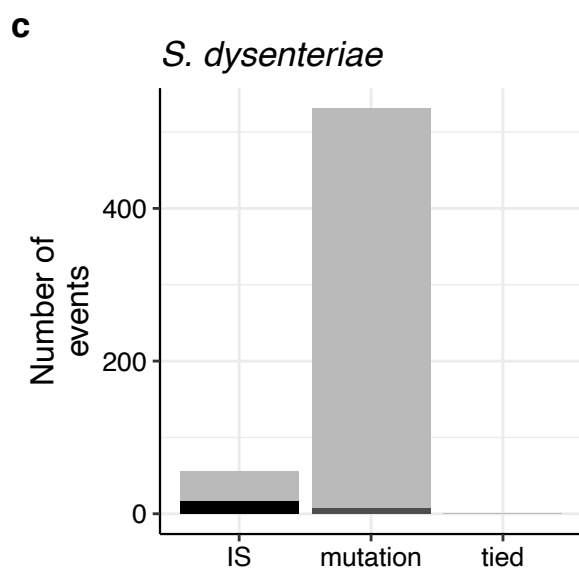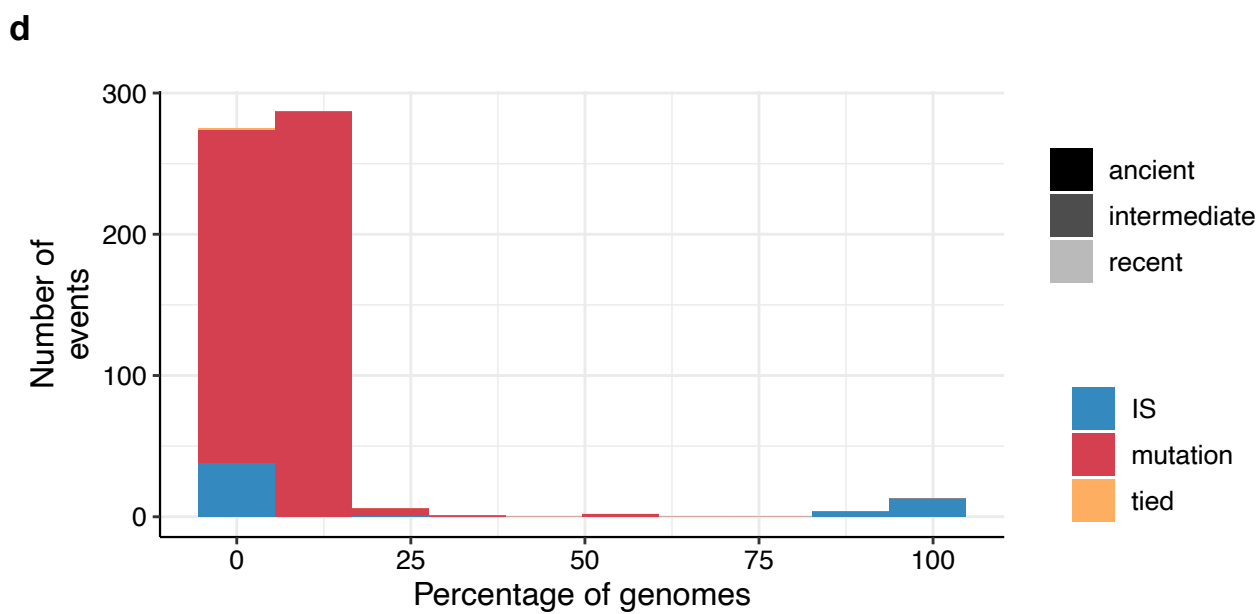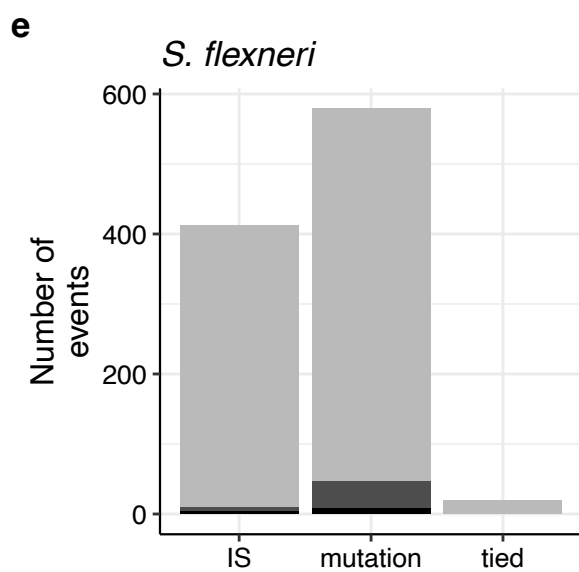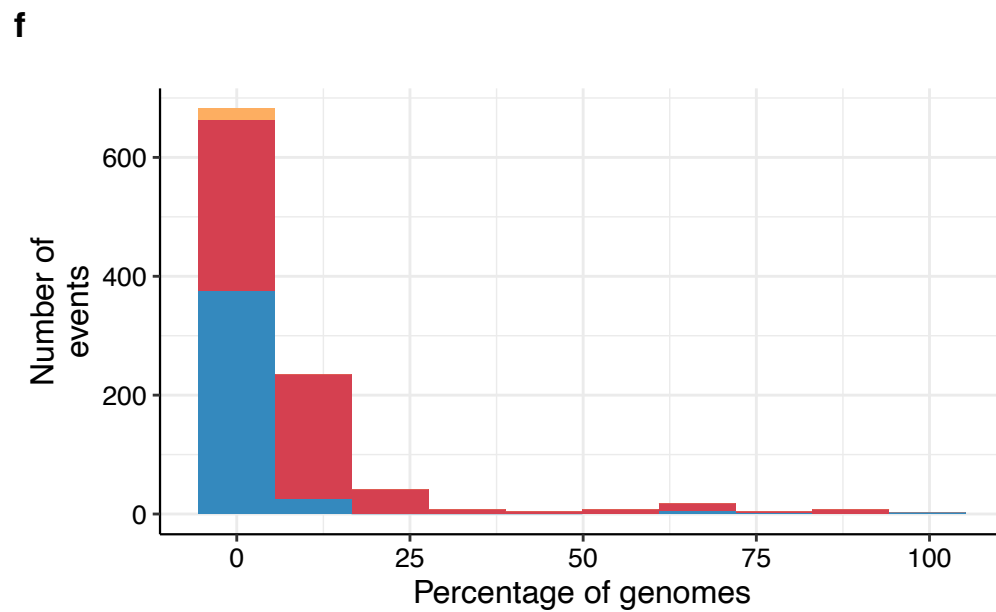

Supplement: S22 Fig — a/c/e, Total number of initial inactivation events by inactivation type (IS, mutation or tied for either). Each type has been broken down by the hypothesised age of the event–ancient events are conserved in ≥ 80% of genomes, intermediate events are present in >20% and <80% of genomes, recent events occur in ≤ 20% of genomes. b/d/f, Percentage of genomes carrying each initial inactivation event, broken down by mechanism of inactivation (as per legend) in each Shigella species. (PDF) [file pgen.1008931.s022.pdf]
