# Supplementary material for: Impact of insertion sequences on convergent evolution of Shigella species
Source: PLoS Genet. 2020 Jul 9;16(7):e1008931. doi: 10.1371/journal.pgen.1008931 (PMC7373316; doi:10.1371/journal.pgen.1008931)
Supplement: S7 Fig — All trees are midpoint rooted. Scale bars show number of substitutions per site. Arrows and labels in panel a indicate clade locations of known IS1 variants listed on ISFinder. (PDF) [file pgen.1008931.s007.pdf]

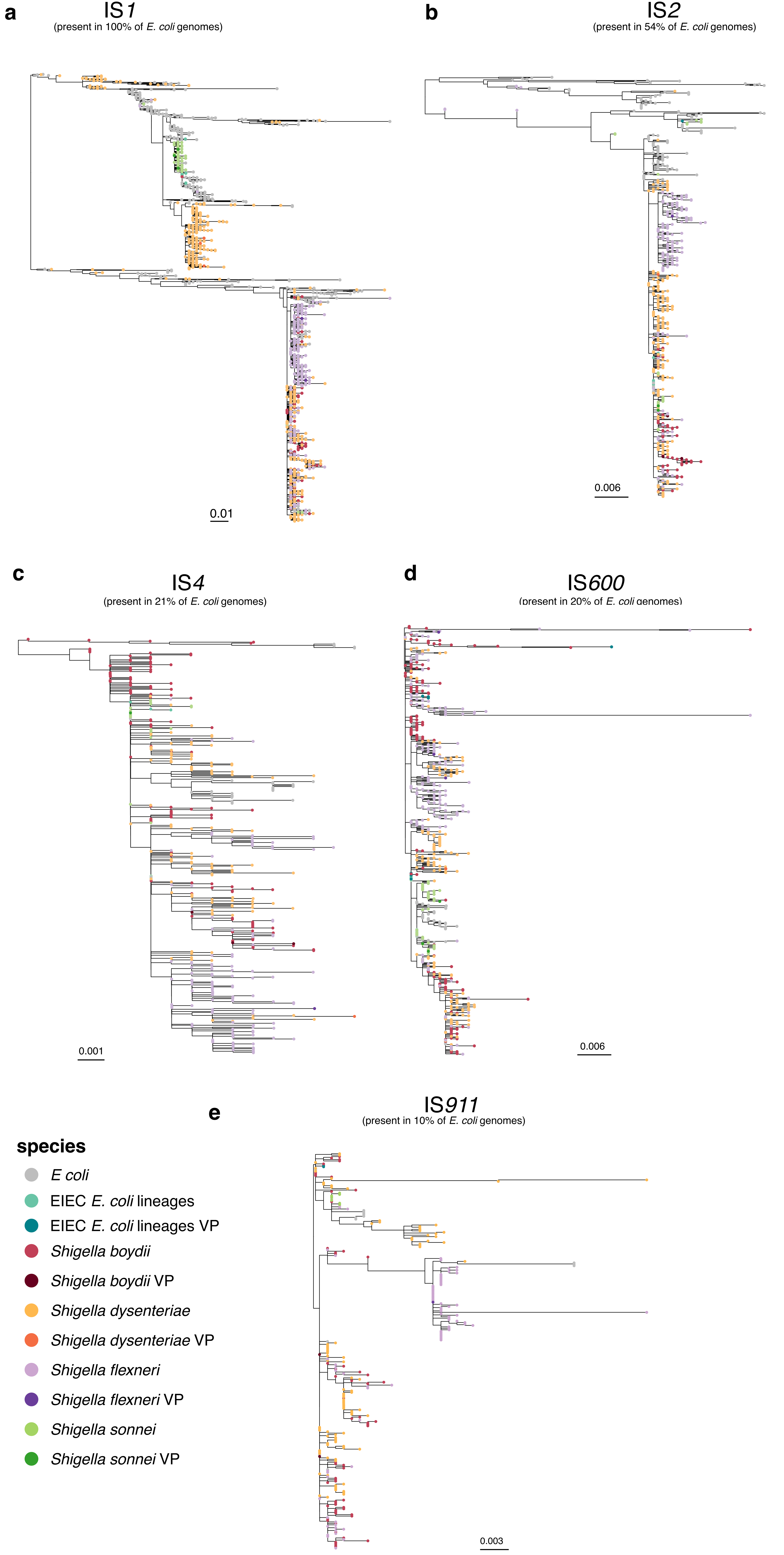
a
IS*1*
(present in 100% of *E. coli* genomes)
0.01
b
IS*2*
(present in 54% of *E. coli* genomes)
0.006
c
IS*4*
(present in 21% of *E. coli* genomes)
0.001
d
IS*600*
(present in 20% of *E. coli* genomes)
0.006
e
IS*911*
(present in 10% of *E. coli* genomes)
0.003
species
E coli
EIEC *E. coli* lineages
EIEC *E. coli* lineages VP
Shigella boydii
Shigella boydii VP
Shigella dysenteriae
Shigella dysenteriae VP
Shigella flexneri
Shigella flexneri VP
Shigella sonnei
Shigella sonnei VP
