# Supplementary material for: Impact of insertion sequences on convergent evolution of Shigella species
Source: PLoS Genet. 2020 Jul 9;16(7):e1008931. doi: 10.1371/journal.pgen.1008931 (PMC7373316; doi:10.1371/journal.pgen.1008931)
Supplement: S11 Fig — a, Null distributions (grey) of the number of random genes that overlap with observed fixed pseudogenes in S. dysenteriae, with the observed overlap shown as a red dot. b, Table summarising the observed overlap values, including the percentile and p-value of the real value (red dot in panel (a)) as compared to the null distribution. c, Null distributions (grey) of the number of random genes that overlap with observed fixed or missing pseudogenes in S. dysenteriae, with the observed overlap shown as a red dot. d, Table summarising the observed overlap values, including the percentile and p-value of the real value (red dot in panel (a)) as compared to the null distribution. (PDF) [file pgen.1008931.s011.pdf]

a

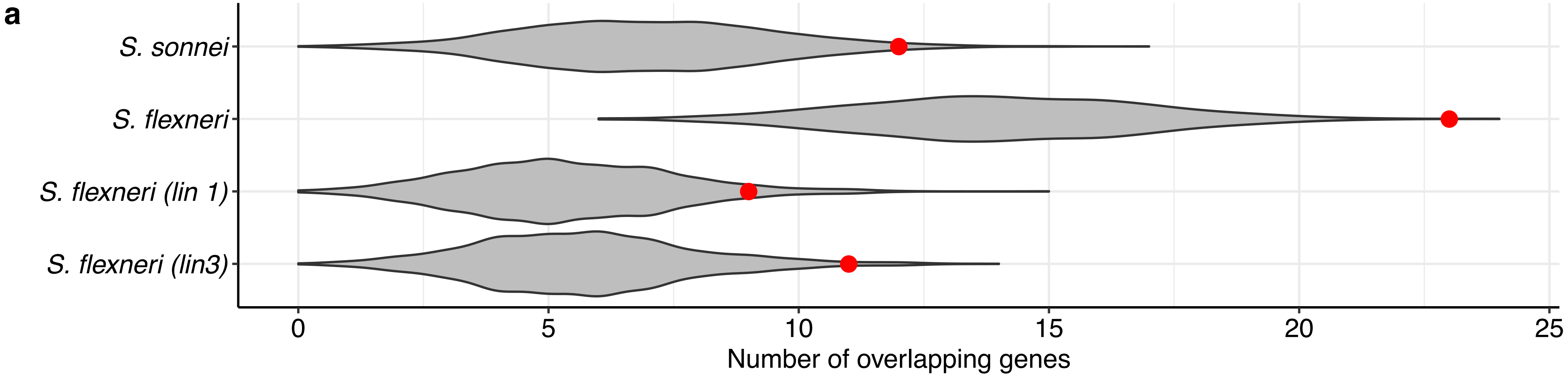

b

comparison	real overlap	percentile	pvalue
S. sonnei	12	0.987	0.013
S. flexneri	23	0.999	0.001
S. flexneri (lin 1)	9	0.967	0.033
S. flexneri (lin 3)	11	0.988	0.012

c

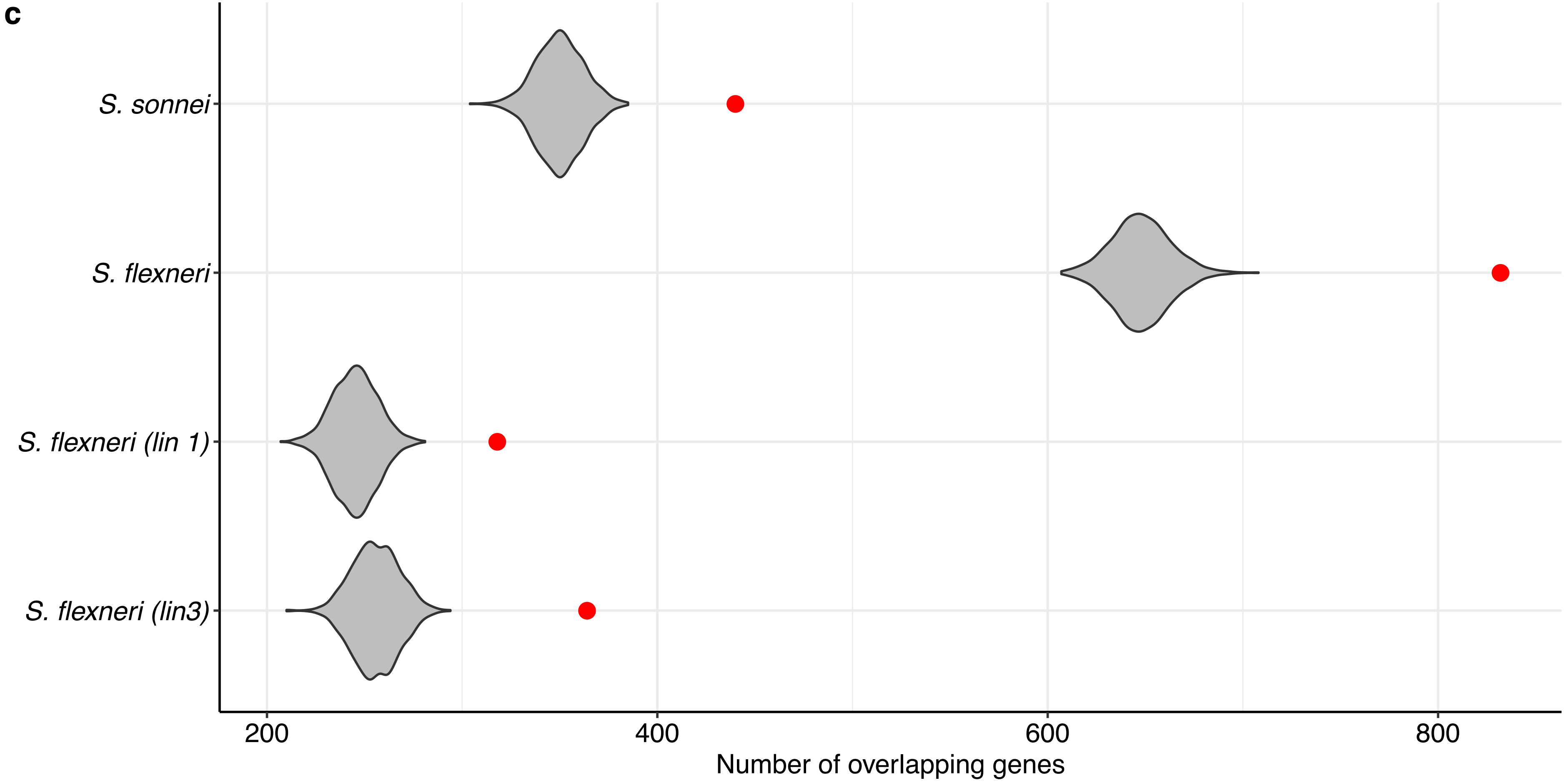

d

comparison	real overlap	percentile	pvalue
S. sonnei	440	1	<0.001
S. flexneri	832	1	<0.001
S. flexneri (lin 1)	318	1	<0.001
S. flexneri (lin 3)	364	1	<0.001
