# Supplementary material for: Impact of insertion sequences on convergent evolution of Shigella species
Source: PLoS Genet. 2020 Jul 9;16(7):e1008931. doi: 10.1371/journal.pgen.1008931 (PMC7373316; doi:10.1371/journal.pgen.1008931)
Supplement: S13 Fig — Phenotypes (columns) and genomes (rows) are ordered via hierarchical clustering of the data matrix, cluster dendrograms are shown. Rows are annotated to indicate which species and lineage each genome belongs to, according to inset legend. Heatmap cells are coloured by the substrate class as per inset legend, with the lightest colour indicating that the phenotype is absent in that genome. Corresponding data is provided in S10 Table. (PDF) [file pgen.1008931.s013.pdf]

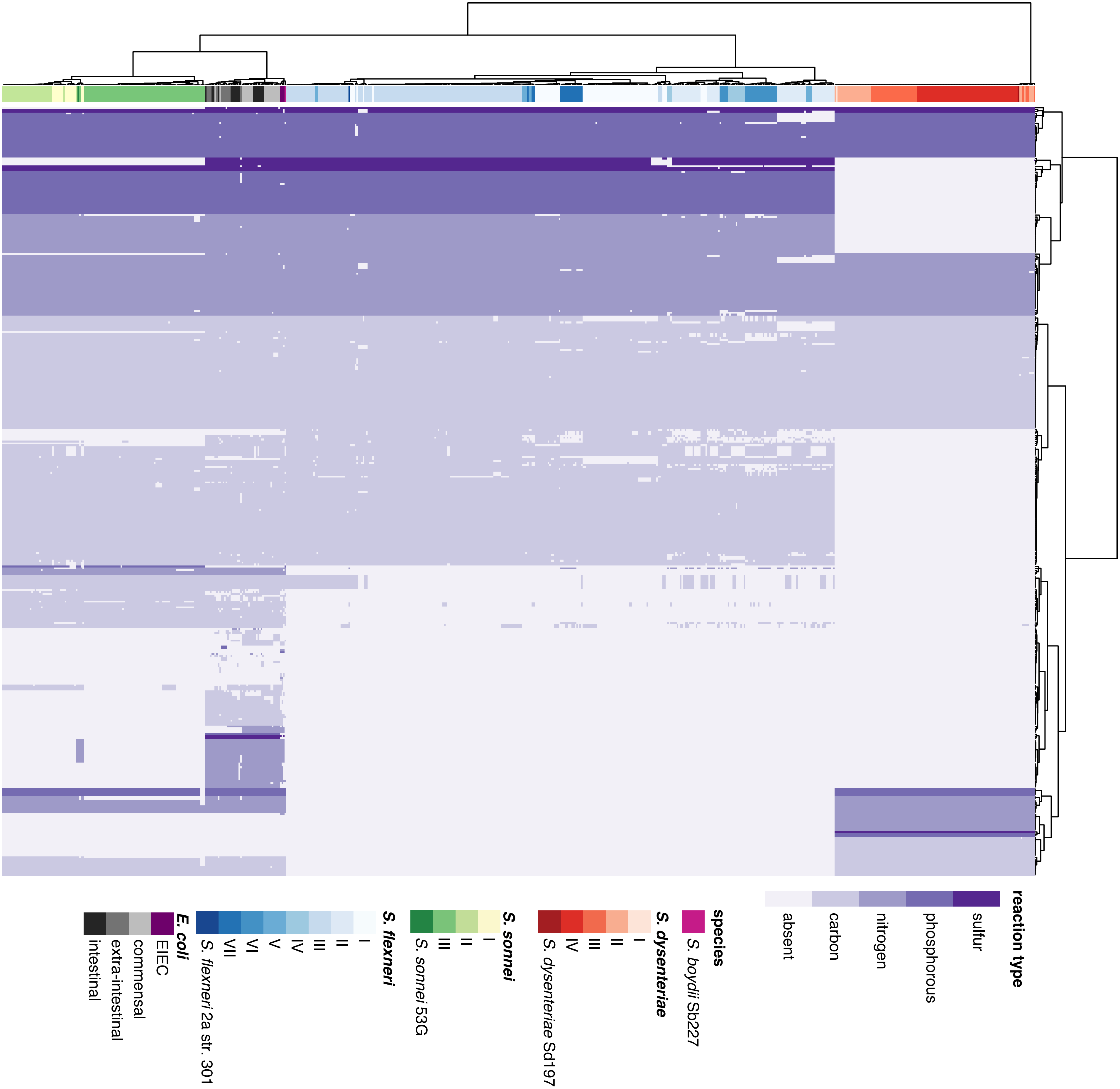
E. coli
EIEC
commensal
extra-intestinal
intestinal
S. flexneri
I
II
III
IV
V
VI
VII
S. flexneri 2a str. 301
S. sonnei
I
II
III
S. sonnei 53G
S. dysenteriae
I
II
III
IV
S. dysenteriae Sd197
species
S. boydii Sb227
reaction type
sulfur
phosphorous
nitrogen
carbon
absent
