# Supplementary material for: Impact of insertion sequences on convergent evolution of Shigella species
Source: PLoS Genet. 2020 Jul 9;16(7):e1008931. doi: 10.1371/journal.pgen.1008931 (PMC7373316; doi:10.1371/journal.pgen.1008931)
Supplement: S21 Fig — a, Null distributions (grey) of the number of random phenotypes that overlap with core E. coli phenotypes, with observed overlap shown as a red dot. b, Table summarising the observed overlap values, including the percentile and p-value of the real value (red dot in panel (a)) as compared to the null distribution. (PDF) [file pgen.1008931.s021.pdf]

a

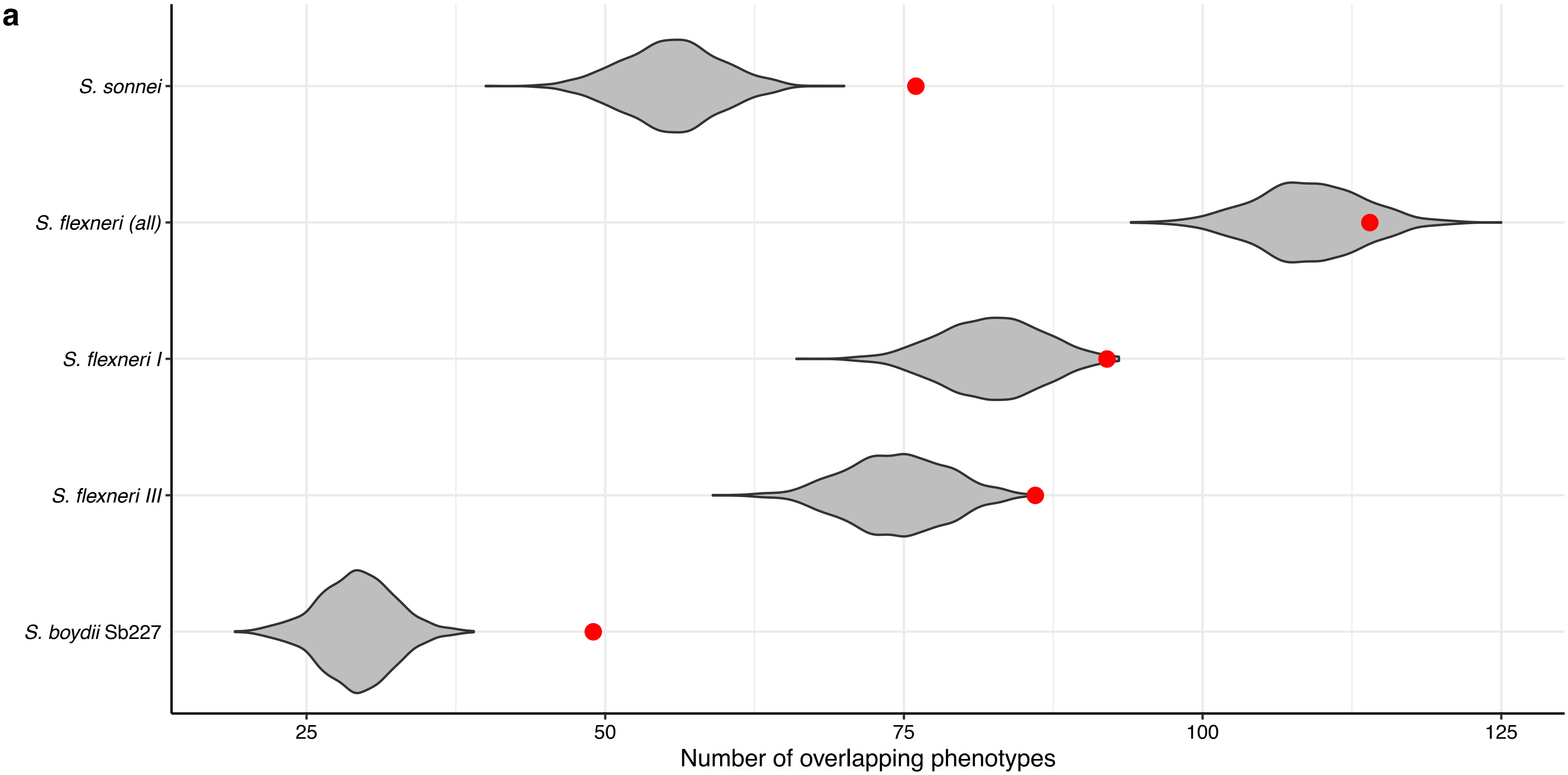

b

Species	# core E. coli phenotypes lost	# core phenotypes lost in common with Sd1	percentile	pvalue
S. dysenteriae	181	NA	NA	NA
S. sonnei	97	76 (78%)	1.0	<0.001
S. flexneri (all)	190	114 (60%)	0.89	0.11
S. flexneri I	144	92 (64%)	0.99	<0.001
S. flexneri III	130	86 (66%)	1.0	<0.001
S. boydii Sb227	51	49 (96%)	1.0	<0.001
