# Supplementary material for: Impact of insertion sequences on convergent evolution of Shigella species
Source: PLoS Genet. 2020 Jul 9;16(7):e1008931. doi: 10.1371/journal.pgen.1008931 (PMC7373316; doi:10.1371/journal.pgen.1008931)
Supplement: S1 Text — (DOCX) [file pgen.1008931.s023.docx]

**Supplementary Text**

As summarised in the main text, we used maximum parsimony ancestral state reconstruction to infer the presence of each IS insertion at internal nodes of the dated phylogeny (**Fig 2e**), and interpreted transitions between inferred states at linked nodes as IS gain/loss events (see **Methods**). The presence or absence of each IS site was determined on each internal node of the dated phylogeny produced by Holt *et. al,* 2012 using maximum parsimony ancestral state reconstruction, implemented in the *ancestral.pars* function in the R package phangorn v2.1.1^1^. For each IS site, the number of events inferred across the tree (either gain or loss) was calculated as follows. For nodes where the IS insertion was inferred to be absent, but inferred as present on its parent node, a loss event was recorded. For nodes where the IS insertion was inferred to be present, but inferred as absent on its parent node, a gain event was recorded. If there was no change in the inferred IS state between the current node and the parent node, then no event was recorded. These results were collated to determine the total number of gain and loss events occurring on each branch (excluding the deep branches leading to MRCA_L1_ and MRCA_L2,L3_, due to uncertainty in the ancestral state reconstruction at these nodes), across all IS insertions. **S5a Fig & S5b Fig** shows that, for each IS, the total number of gains across the species tree is correlated with the relative activity level of the IS as assessed by the number of strain-specific insertions in that species, which gives some confidence in the reconstruction.

To further assess the accuracy of the reconstruction, for each inferred loss event, we considered how frequently sequential gain and loss events were inferred at the same site. As the IS under study do not have site-specific target sequences, re-insertion at the same site following a loss is unlikely, hence we expect to see few instances of an inferred loss event followed by an inferred gain at the same site in descendant branches. However as an IS can be lost through a variety of mechanisms including excision and deletion, we do expect to see more instances of an inferred gain event followed by an inferred loss event at the same site in descendant branches. Of the 1656 loss events inferred on the *S. sonnei* phylogeny, 54 were followed by an inferred gain at the same site, implying re-insertion frequency of 3.2%. As expected, IS gain followed by loss was inferred significantly more frequently (OR 4.7, p<1x10^-16^): of 1501 inferred gain events in *S. sonnei*, 207 (13.8%) were followed by an inferred loss. If all inferred re-insertions represent errors in the reconstruction, this would imply <5% errors and thus accuracy of >95%.

From this analysis in *S. sonnei*, we estimate the MRCA of lineage I (MRCA_L1_, circa 1832) carried 220 IS insertions, whilst the MRCAs of lineages II (MRCA_L2_, circa 1817) and III (MRCA_L3_, circa 1883) carried 275 and 286, respectively – higher than the load reached by contemporary isolates of lineage I (median 243; **Fig 2e**). It is not possible to reliably reconstruct the status in the *S. sonnei* MRCA (MRCA_SS_) of IS insertions that were present in the MRCA of lineages II and III (MRCA_L2,L3_) but absent in MRCA_L1_ (55 sites, **S4 Table**), or present in MRCA_L1_ and absent from MRCA_L2,L3_ (12 sites, **S4 Table**), as we lack a relevant outgroup. We therefore considered two alternative explanations: (i) MRCA_SS_ carried only the 208 IS present in both MRCA_L1_ and MRCA_L2,L3_, followed by a rapid gain of IS on the branch leading to MRCA_L2,L3_ (55 IS over 35 years [1.6 per year, 82% IS*1*]), and a more typical rate of IS gain on the branch leading to MRCA_L1_ (15 IS over 164 years [0.1 per year, 53% IS*1*]) (black in **Fig 2e**); or (ii) MRCA_SS_ carried all 255 IS that were present in MRCA_L1_ and/or MRCA_L2,L3_, followed by a typical rate of IS gain in the branch leading to MRCA_L2,L3_ (12 IS over 35 years [0.3 per year, 67% IS*1*]) and substantial loss of IS on the branch leading to MRCA_L1_ (55 IS lost over a 164 year period [64% IS*1*]). We propose that substantial IS loss would most likely be driven by IS-mediated deletions, and thus be accompanied by a decrease in genome size in Lineage I; however we found no evidence of genome size difference between lineages (based on assembly size after removing low coverage contigs, or contigs smaller than 200 bp). Given the overrepresentation of IS*1* amongst sites present in MRCA_L2,L3_ but not MRCA_L1_, we therefore propose the most plausible explanation is accelerated expansion of IS*1* on the branch leading to MRCA_L2,L3_.

**References**
1. Schliep, K. phangorn: phylogenetic analysis in R. *Bioinformatics* **27,** 592 593 (2011).
