# Supplementary material for: Impact of insertion sequences on convergent evolution of Shigella species
Source: PLoS Genet. 2020 Jul 9;16(7):e1008931. doi: 10.1371/journal.pgen.1008931 (PMC7373316; doi:10.1371/journal.pgen.1008931)

Shigella sonnei alternate carbon sources

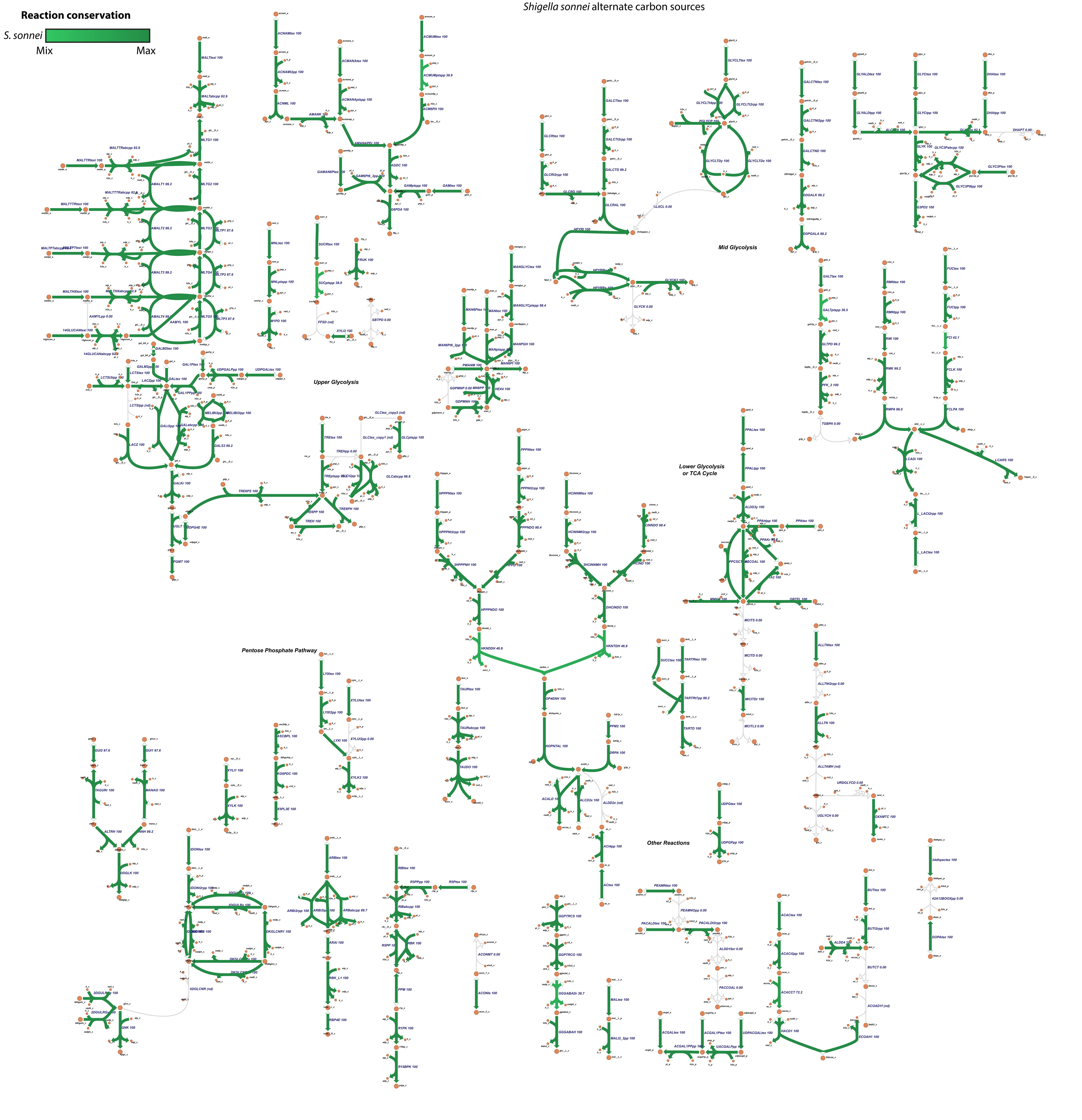

Supplement: S20 Fig — Arrows are coloured by the percentage of genomes within the species that can use that reaction (as per inset legend), based on GEMS predictions for n = 126 strains; grey indicates that the reaction is absent from all genomes. Each reaction is labelled with its name and the percentage of genomes where the reaction is present. Orange circles indicate intermediate metabolites. (PDF) [file pgen.1008931.s020.pdf]
